# Recent advances in proteomic strategies for target identification of traditional Chinese medicine

**DOI:** 10.1016/j.jpha.2025.101516

**Published:** 2025-12-10

**Authors:** Xueyan Zhen, Jingwen Liu, Yan Ren

**Affiliations:** Experiment Center for Science and Technology, Shanghai University of Traditional Chinese Medicine, Shanghai, 201203, China

**Keywords:** Proteomics strategies, Target protein identification, Traditional Chinese medicine

## Abstract

Traditional Chinese medicine (TCM) has played an indispensable role in health intervention and disease treatment. Identifying the target proteins of TCM is crucial to clarifying therapeutic mechanisms. One approach taken to enhance the breadth, depth, and precision of studies on the active cellular pathways induced by TCM is to use proteomics to reveal potential drug targets with direct interactions. Proteomic strategies facilitate identifying and characterizing target binding proteins relevant to metabolic pathways, which involves enriching the complex of small molecule and their targets based on the affinity, as well as utilizing the changes in physicochemical properties of target proteins that occur due to drug binding for proteomic identification and quantification. Probe labeling and enrichment technologies have accelerated the field of chemical proteomics. Technologies focused on measuring the changes in target proteins have been widely extended into several different approaches, and these now drive the establishment of further strategies. This review summarizes the advances in proteomics strategies mainly based on mass spectrometry (MS) for the identification of TCM targets to the present. Enhancing the application of proteomics would provide a new viewpoint on TCM treatment, while underscores the potential of TCM as biological probes and sources of novel drug candidates.

## Highlights

• Innovative proteomic techniques enhance drug-target identification in more sensitive ways.
• Recent advances in proteomics strategies for the identification of TCM targets were included.
• Integrating different approaches proves more conducive in unveiling potential targets of TCM and its active ingredients.

## Graphical abstract

**Figure:**
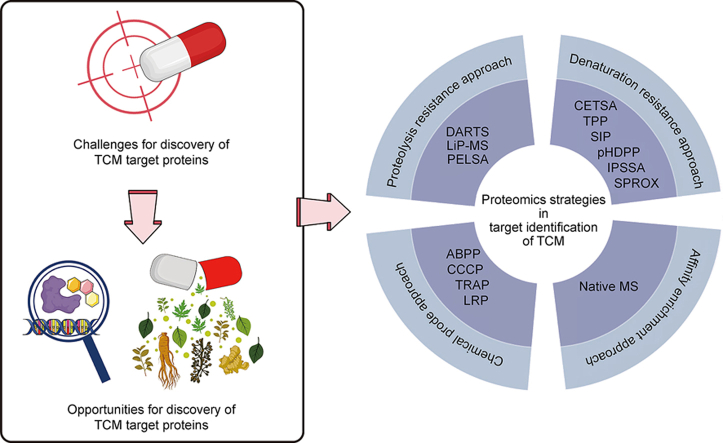

## 1 Introduction

Traditional Chinese medicine (TCM), with a history spanning thousands of years, generally refers to using a mixture of herbal plants or extracts that contain many bioactive components with varying physicochemical characteristics [1,2]. These bioactive components typically have multiple targets by which they exert their health effects and functions in disease intervention and treatment [3]. Recent clinical studies indicate that TCM components have significant therapeutic and preventative benefits, notably as treatments for coronavirus disease 2019 (COVID-19) [4]. Due to the unique pharmacological activity of herbal medicines, TCM presents valuable opportunities for discovery and research, especially those that can target cancer [5] and infectious diseases [6] or can prevent cardiovascular and metabolic disorders [7,8], rheumatoid arthritis [9,10], and multiple sclerosis [11]. Identifying the target proteins is essential for the molecular mechanisms of TCM; however, this deconvolution process is time-consuming [12,13]. Moreover, multiple bioactive compounds in TCM, along with their complementary functions, present major barriers to investigating the underlying molecular mechanisms.

**Fig. 1:**
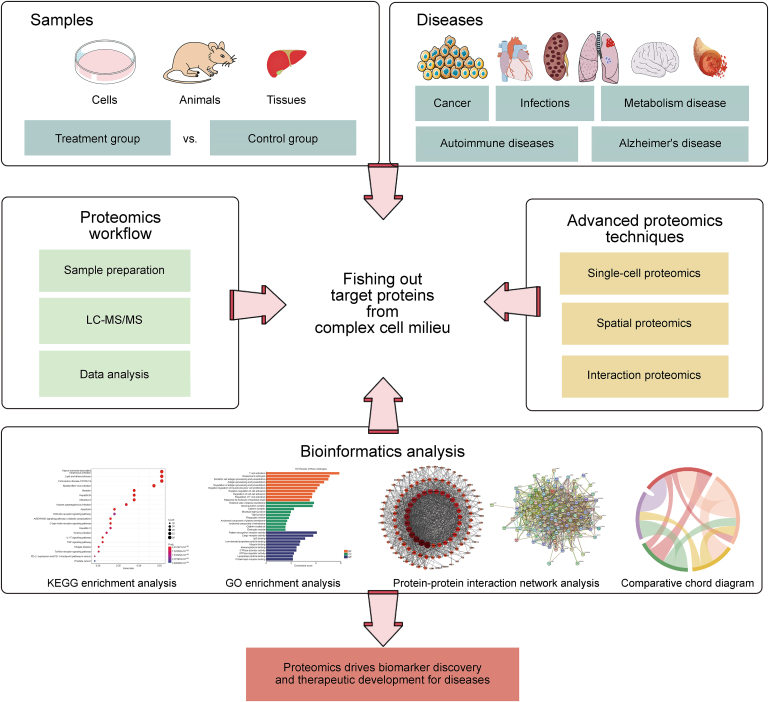
Fishing out target proteins from complex cell millieu. LC-MS/MS: liquid chromatography-tandem mass spectrometry; COVID-19: coronavirus disease 2019; NOD: nucleotide-binding oligomerization domain; ARE: advanced glycation end products; RAGE: receptor of advanced glycation end products; IL-17: interleukin-17; TNF: tumour necrosis factor; PD-L1: programmed death ligand 1; PD-1: programmed death 1; KEGG: kyoto encyclopedia of genes and genomes; GO: Gene Ontology; BP: biological process; CC: cellular component; MF: molecular function. Created by using BioRender.com.

Fishing out target proteins from the complex cell milieu is challenging, however, the proteomic approach could effectively address this issue [14,15]. The complex effects induced by bioactive compounds can be revealed by proteomic identification of their targets and network analysis of the pathways [16,17]. Proteomic technologies focus on screening significant changes in the abundance or modification of proteins, thereby facilitating biomarker discovery and revealing the functional proteins or pathways involved in biological events [18]. Due to its high sensitivity and resolution, proteomics is crucial and offers strong potential for characterizing global protein changes, diagnosing and treating major diseases, such as cancer [19], autoimmune disease [20], Alzheimer's disease [21], infections [22], and obesity [23]. Nowadays, advanced proteomics techniques for elucidating drug-target interaction mechanisms have been continually developed and introduced (Fig. 1).

Leveraging the specific properties of drugs and proteins is a logical consideration for identifying drug target proteins within the complexes formed between drugs and their binding proteins. These techniques monitor the changes in the stability, proteolysis, and chemical reactions of the target proteins after drug binding and have been developed and widely extended to different approaches, including the cellular thermal shift assay (CETSA), thermal proteome profiling (TPP), protein stability determinations from rates of oxidation (SPROX), the drug affinity responsive target stability (DARTS), limited proteolysis-mass spectrometry (LiP-MS), peptide-centric local stability assay (PELSA), solvent-induced protein precipitation (SIP), pH-dependent protein precipitation (pHDPP), and integrated protein solubility shift assay (IPSSA). For chemical proteomics, modified drugs or probes are usually needed to specifically enrich the conjugate of its target proteins, which have the advantages of increasing the sensitivity, reproducibility, and flux to overcome the limitations of classical gel separation in identifying low-abundance protein [24,25]. Chemical proteomics technologies consist of two key steps: i) probe design and synthesis and ii) target fishing and protein identification, including compound-centric chemical proteomics (CCCP), activity-based protein profiling (ABPP), target responsive accessibility profiling (TRAP), and lysine reactivity profiling (LRP), which are widely used for drug target identification [26]. The application of these methods for TCM target identification is summarized as follows.

Recently, a better understanding and more in-depth exploration of the mechanism of TCM has been attained by the application of proteomic strategies in a growing number of studies on biomolecule-target relationships. This review comprehensively summarizes emerging proteomic approaches for TCM target identification, critically evaluating their respective strengths, limitations, and applicability. By systematically analyzing these innovative methodologies, this study aims to construct a framework for addressing persistent technical challenges in the discovery of TCM target proteins, while offering strategic perspectives to advance precision in TCM.

## 2 Challenges and opportunities for the discovery of TCM target proteins

**Fig. 2:**
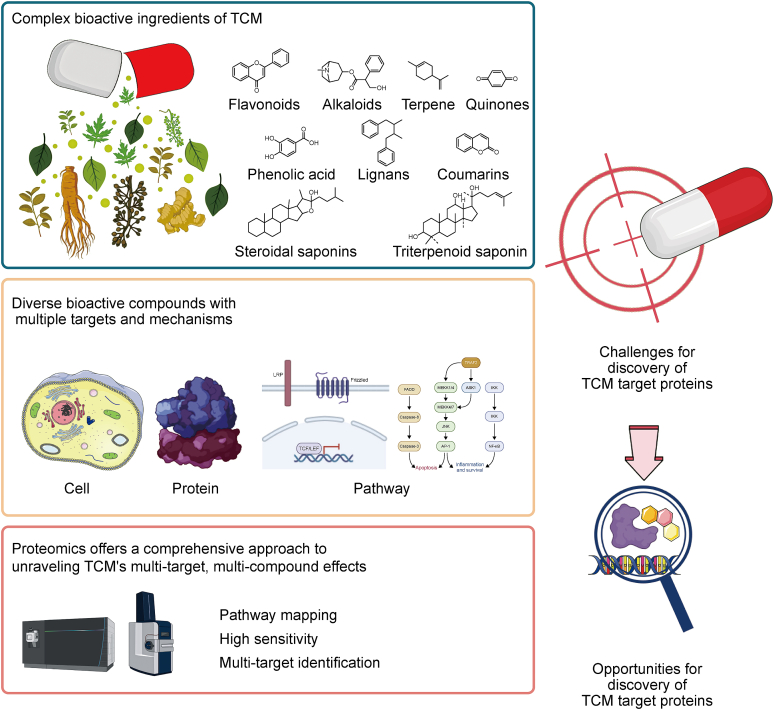
Challenges and opportunities for the discovery of traditional Chinese medicine (TCM) target proteins. LRP: low-density lipoprotein receptor-related protein; TCF/LEF: T-cell factor/lymphoid enhancer-binding factor; FADD: Fas-associating protein with a novel death domain; MEKK: mitogen-activated protein kinase (MAPK)/extracellular signal-regulated kinase kinase kinase; JNK: c-Jun N-terminal kinase 1; AP-1: activator protein-1; ASK1: apoptosis signal-regulating kinase 1; NIK: nuclear factor-kappaB (NF-κB)-inducing kinase; IKK: IκB kinase. Created by using BioRender.com.

In recent years, the recognized advantages and health benefits of TCM have gained increasing attention in studies focused on preventing and treating diseases, as a widely used source of core raw materials for natural drugs [27,28]. The bioactive chemical compounds of TCM include volatile oils, alkaloids, flavonoids, glycosides, terpenoids, phenylpropanoids, phenolic acids, phenols, quinones, lactones, and steroid compounds [[29], [30], [31]]; therefore, identifying and clarifying the targets of bioactive TCM components is the basis for elucidating the pharmacological action of TCM. However, the diversity and complexity of TCM components create challenges in studying TCM mechanisms, which often involve multiple processes and targets (Fig. 2). To address these challenges, proteomic strategies have become an indispensable tool in the TCM research arsenal, offering distinct advantages for each approach [32]. Extensive studies have confirmed that the functional mechanisms of TCM extracts or formulas can be characterized using proteomic techniques. Exploring the molecular mechanisms of TCM remains the main theme of ongoing research in this field.

The rapid development of proteomic strategies has significantly advanced the identification of molecular target in complex TCM extracts, eliminating the need for extensive fractionation of the raw material. For example, cryptotanshinone (CTS) and CTS-containing extracts from the roots of *Salvia miltiorrhiza* have been confirmed by DARTS as a promising treatment for keratinopathic ichthyosis by targeting the FKBP prolyl isomerase 1A (FKBP1A) protein [33]. Similarly, an ethanol extract of *Potentilla glabra* var. mandshurica (Maxim.) Hand.-Mazz (Pg-EE) has shown therapeutic potential for autoimmune disorders, arthritis, and diabetes, conditions characterized by excessive and persistent inflammatory responses [34]. Recent application of CETSA confirmed the specific interaction of Pg-EE with its target, the Src protein tyrosine kinase (Src) [35]. Moreover, another study on ethanol extract from *Cissus subtetragona* (Cs-EE) revealed its anti-inflammatory effects, including the inhibition of inflammatory cytokines and the reduction of luciferase activity in nuclear factor-kappaB (NF-κB) and activator protein-1 (AP-1). Further investigation using CETSA technology confirmed proto-oncogene tyrosine-protein kinase Src (Src) and transforming growth factor-β (TGF-β)-activated kinase 1 (TAK1) proteins as key targets of Cs-EE [36].

**Fig. 3:**
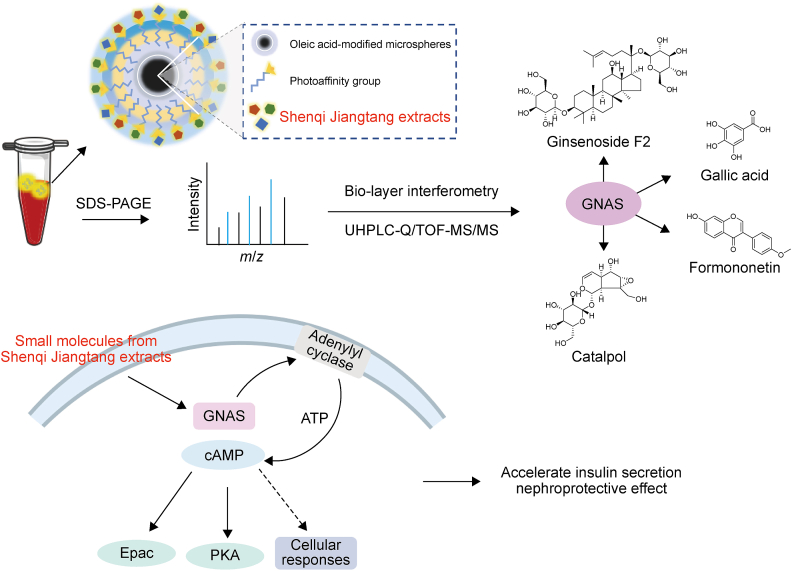
Traditional Chinese medicine microspheres (TCM-MPs) target fishing strategy combined with bio-layer interferometry (BLI) reverse fishing technology to screen out guanine nucleotide-binding protein, alpha stimulating (GNAS) key proteins that directly interact with active ingredients (formononetin, gallic acid, ginsenoside F2, and catalpol) from complex target protein systems. SDS-PAGE: sodium dodecyl sulfate-polyacrylamide gel electrophoresis; UHPLC-Q/TOF-MS/MS: ultra-high performance liquid chromatography coupled with quadrupole time-of-flight tandem mass spectrometry; ATP: adenosine 5′-triphosphate; cAMP: cyclic adenosine monophosphate; Epac: exchange protein activated by cAMP; PKA: protein kinase A.

Recently, Tu and colleagues [37] have developed a TCM microspheres (TCM-MPs) target fishing strategy that enables the self-assembly and fixation of drug-active small molecules with non-selective properties (Fig. 3). In their work, using Shenqi Jiangtang granules (a TCM preparations for type 2 diabetes) as an example, this innovative approach provides a new technique for drug target discovery-constructing TCM-MPs to fish for target proteins and combining this with bio-layer interferometry (BLI) to perform reverse screening of active molecules against the target proteins. The highlight lies in fixing different types of active small molecules in TCM to establish an effective target discovery technology, showing promising potential for identifying and investigating targets within complex systems.

## 3 Proteomic strategies for the identification of TCM targets

Proteomic strategies for target discovery focus on differentiating the whole complex of biomolecules and their target proteins from the non-binding proteins in the cell protein reservoir. Therefore, it is mainly based on biomolecules-protein binding interactions that lead to conformational and protein stability changes or that can be used as bait for fishing out a biomolecules-protein complex by affinity enrichment. The physicochemical property changes in target proteins after binding enable the division of the techniques into proteolysis resistance approach, denaturation resistance approach, and chemical probe approach. The details of these individual methods derived from the proteolysis resistance approach (DARTS, LiP-MS, and PELSA), the denaturation resistance approach (CETSA, TPP, SIP, pHDPP, IPSSA, and SPROX), the chemical probe approach (ABPP, CCCP, TRAP, and LRP), and the biomolecules-protein complex by affinity enrichment (Native mass) have been reviewed as well.

### 3.1 Proteolysis resistance approach

#### 3.1.1 DARTS

**Fig. 4:**
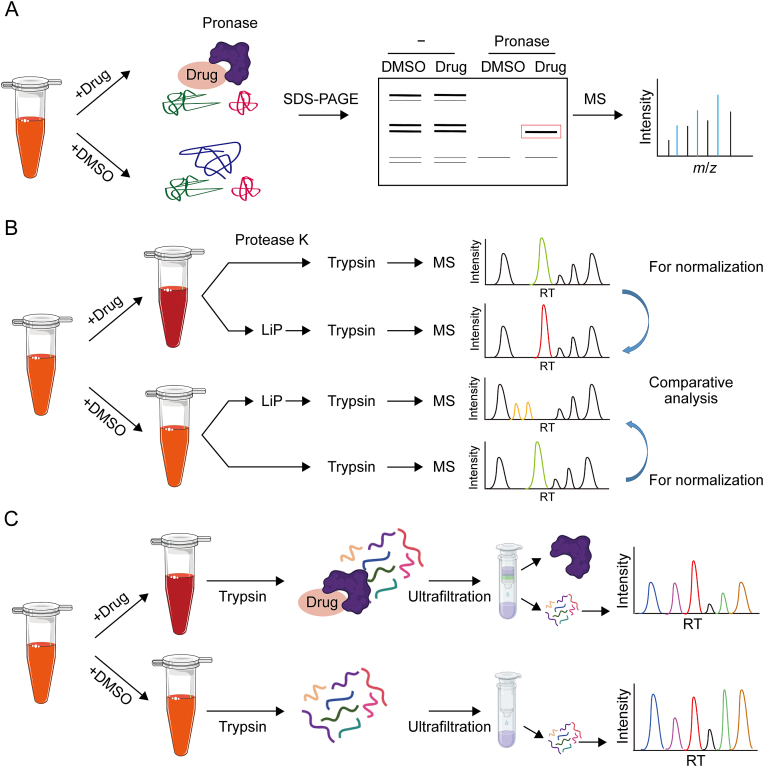
Workflow diagram of proteolysis resistance approach for target identification: (A) drug affinity responsive target stability (DARTS), (B) limited proteolysis-mass spectrometry (LiP-MS), and (C) peptide-centric local stability assay (PELSA). SDS-PAGE: sodium dodecyl sulfate-polyacrylamide gel electrophoresis; DMSO: dimethyl sulfoxide; LiP: limited proteolysis; RT: retention time.

DARTS is a well-established and powerful method for studying drug-binding proteins, based on the principle that drug-target protein complexes are more resistant to proteolysis than free proteins [38]. Drug binding induces the target proteins to assume a specific conformation that masks protease recognition sites, thereby imparting greater resistance to proteolysis. These changes are visualized on sodium dodecyl sulfate-polyacrylamide gel electrophoresis (SDS-PAGE) and identified by liquid chromatography-tandem MS (LC-MS/MS) [39] (Fig. 4A).

A key advantage of DARTS is its applicability without requiring chemical or structural modification of bioactive TCM molecules [40] (Table 1) [33,[41], [42], [43], [44], [45], [46], [47], [48], [49], [50], [51], [52], [53]]. For instance, it has revealed that shizukaol A exerts its anti-inflammatory effects by targeting high mobility group box 1 (HMGB1), thereby modulating the nuclear factor erythroid 2-related factor 2/heme oxygenase 1 (Nrf2/HO-1) signaling pathway [41]. Similary, based on the DARTS approach, tumor necrosis factor (TNF) receptor-associated factor 6 (TRAF6) was identified as a direct target of trishizukaol A (TSA), a bioactive candidate from *Sarcandra glabra*, which suppresses inflammatory fluxes through the mitogen-activated protein kinase (MAPK) pathway [42]. In addition, quantitative proteomics and DARTS assays have further shown that daurisoline, isolated from Menispermi Rhizoma, targets heat shock protein 90 (HSP90) in A549 and Hop62 lung cancer cell lines, suggesting its potential therapeutic target for fighting lung cancer [43]. Furthermore, salicin (SA), derived from willow bark (*Salix alba*), alleviates osteoarthritis by directly targeting the endoplasmic reticulum stress regulator inositol-requiring enzyme 1α (IRE1α) in primary articular chondrocytes [44]. Likewise, liquidambaric acid (LDA) has been identified as a direct target of TRAF2, inhibiting Wnt/β-catenin signaling and presenting new horizons in the treatment of colon cancer [45]. Moreover, dihydromyricetin (DHM), extracted from *Ampelopsis grossedentata*, has been identified as targeting the binding site of glucose regulated protein 78 (GRP78) using DARTS, with direct interactions confirmed by surface plasmon resonance (SPR), elucidating its mechanism in reducing lipid droplet formation in 3T3-L1 cells and highlighting its potential as an anti-obesity agent [46]. Using DARTS and validated by atomic force microscopy imaging, cytosolic phospholipase A2 (cPLA2) has been identified as a direct target of aconitine from the *Aconitum* species, inducing prostaglandin G/H synthase 2 (PTGS2)/cyclooxygenase-2 (COX2) expression and inflammatory factor release, ultimately contributing to myocardial injury and dysfunction [47]. Diosmin, screened as an aryl hydrocarbon receptor (AhR) agonist through luciferase assays and confirmed by DARTS, has been shown to directly bind to AhR, upregulating skin barrier proteins (filaggrin and loricrin) and restoring barrier function suppressed by T helper 2 cell (Th2) cytokines, highlighting its potential as a treatment for atopic dermatitis [48]. Additionally, deoxyelephantopin (DET), a sesquiterpene lactone isolated from *Elephantopus scaber* Linn., has been demonstrated significant cytotoxicity against HepG2 and Hep3B hepatocellular carcinoma (HCC) cells. Using DARTS assay, DET was identified as a direct binder of HSP90 and further studies have revealed that DET induced mitochondrial dysfunction, oxidative stress, and apoptosis, both alone and synergistically with sorafenib, making it a promising candidate for HCC therapy [49]. In addition, natural biomolecular, known for their multi-target characteristics, hold promising therapeutic potential. Glytabastan B, a coumestan isolated from *Glycine tabacina*, has been found to target the extracellular regulated protein kinase 2 (ERK2), c-Jun N-terminal kinase 1 (JNK1), and phosphatidylin-ositol-3-kinase (PI3K) catalytic subunit p110 (α and β), effectively inhibiting the MAPK and PI3K/protein kinase B (Akt) pathways, which suggests glytabastan B is a promising multiple-target candidate for the prevention of rheumatoid arthritis [50].

**Table 1:** Proteolysis resistance approach for target identification and validation of traditional Chinese medicines (TCMs).

Proteomic strategies	Compounds	Protein target	Cell model/animal model	Refs.
DARTS	CTS	FKBP1A	HaCaT cells	[33]
	Shizukaol A	HMGB1	RAW264.7 cells	[41]
	TSA	TRAF6	RAW264.7 cells	[42]
	Daurisoline	HSP90	A549 and Hop62 cells	[43]
	SA	IRE1α	Primary rat articular chondrocytes	[44]
	LDA	TRAF2	HCT116 cells	[45]
	DHM	GRP78	3T3-L1 cells	[46]
	Aconitine	cPLA2	H9c2 cells	[47]
	Diosmin	AhR	Normal human epidermal keratinocytes	[48]
	DET	HSP90α	HepG2 and Hep3B cells	[49]
	Glytabastan B	ERK2, JNK1, and PI3K catalytic subunit p110 (α and β)	SW982 cells	[50]
	Tubocapsenolide A	SHP-2	U2OS cells	[51]
	Crellastatin A	PARP-1	HeLa cells	[52]
	PPD	AK5	Brain tissues	[53]
LiP-MS	ISO	IQGAP2	HepG2 cells	[59]
	HPF	Dlat	C3H10T1/2 cells	[60]
	Farrerol	UCHL3	HEK293 cells	[61]
	SG	PDK2	SK-N-SH cells	[62]

DARTS: drug affinity responsive target stability; CTS: cryptotanshinone; FKBP1A: FK506-binding protein (FKBP) prolyl isomerase 1A 160; HMGB1: high mobility group box 1; TSA: trishizukaol A; TRAF6: tumor necrosis factor (TNF) receptor-associated factor 6; HSP90: heat shock protein 90; SA: salicin; IRE1α: inositol-requiring enzyme 1α; LDA: liquidambaric acid; DHM: dihydromyricetin; GRP78: glucose regulated protein 78; cPLA2: cytosolic phospholipase A2; AhR: aryl hydrocarbon receptor; DET: deoxyelephantopin; ERK2: extracellular regulated protein kinase 2; JNK1: c-Jun N-terminal kinase 1; PI3K: phosphoinositide-3-kinase; SHP-2: Src homology 2 phosphatase 2; PARP-1: poly-adenosine diphosphat (ADP)-ribose-polymerase-1; PPD: 20(S)-protopanaxadiol; AK5: adenylate kinase 5; LiP-MS: limited proteolysis-mass spectrometry; ISO: isoliquiritigenin; IQGAP2: isoleucine and glutamine (IQ) motif-containing GTPase activating protein 2; HPF: hyperforin; Dlat: dihydrolipoamide *S*-acetyltransferase; UCHL3: ubiquitin C-terminal hydrolase L3; SG: scutellarin; PDK2: pyruvate dehydrogenase kinase 2.

Recently, the integration of multiple proteomic strategies has been widely employed in target identification, enhancing reliability, uncovering dynamic interactions, and improving the efficiency of functional analysis. The combination of DARTS and CETSA methods has identified tubocapsenolide A has been shown to target the Src homology 2 phosphatase 2 (SHP-2) protein tyrosine phosphatase in U2OS cells [51]. Similarly, DARTS combined with targeted-limited proteolysis-multiple reaction monitoring identified poly-adenosine diphosphate (ADP)-ribose-polymerase-1 (PARP-1) as the target of crellastatin A in HeLa cells [52]. Ginsenoside metabolite 20(S)-protopanaxadiol (PPD), an active compound in ginseng, has been identified to target adenylate kinase 5 (AK5) in brain tissues through DARTS and CETSA. Subsequent BLI kinetic analysis and isothermal titration calorimetry (ITC) assays confirmed the specific binding and activation of AK5 by PPD, providing valuable insights into pharmacological effects in the central nervous system [53].

DARTS is a common method due to its simple operation and high throughput; however, it has limitations in accurately reflecting *in vivo* drug action based only on proteins extracted *in vitro* to study drug-target interaction [39]. Additionally, DARTS is less effective for certain proteins, as some stress-related target proteins are insensitive to proteolytic enzymes under native conditions, and others may be present at very low abundance in cell lysates, limiting their detectability [54].

#### 3.1.2 LiP-MS

LiP-MS enables the analysis of protein-small molecule interactions within complex samples and can be applied across native bacterial, yeast, and mammalian systems without chemical modification of proteins [55]. LiP-MS technique identifies binding sites by detecting local changes in protease accessibility caused by small molecule binding, producing condition-specific cleavage products. These fragments are further digested with trypsin, and the sequences and abundances are measured by LC-MS/MS to identify the target proteins and provide structural fingerprints [56]. As a structural proteomics approach, LiP-MS is valuable for detecting drug targets, identifying disease-associated protein structures, assessing protein aggregates, and capturing protein structural changes [57] (Fig. 4B). LiP-MS effectively identifies small-molecule binding sites and provides structural information without chemical modifications. Similar to DARTS as a proteolysis resistance approach, LiP-MS offers higher resolution, identifying ligand-protein binding site structural details with approximately 12 amino acid precision [58]. However, LiP-MS faces challenges with low-abundance proteins, those without MS-detectable peptides, and proteins undergoing conformational changes.

LiP-MS is now gradually being applied for the discovery of TCM targets (Table 1) [[59], [60], [61], [62]]. LiP-MS exploration of the mechanism by which isoliquiritigenin (ISO) effectively ameliorates nonalcoholic steatohepatitis (NASH) symptoms in mice identified IQ motif-containing GTPase activating protein 2 (IQGAP2) as the direct target protein of ISO in HepG2 cells [59]. The binding of IQGAP2 and ISO was found to activate the IQGAP2/cyclic adenosine monophosphate (cAMP)-response element binding protein (CREB)/silent information regulator sirtuin 1 (SIRT1) pathway to alleviate NASH. Chen et al. [60] confirmed that hyperforin (HPF), as a potential anti-obesity agent, promoted thermogenesis via an AMP-activated protein kinase (AMPK)/peroxisome proliferator-activated receptor (PPAR) gamma coactivator-1 alpha (PGC-1α)-dependent pathway. Using LiP-MS combined with microscale thermophoresis (MST) and molecular docking, dihydrolipoamide *S*-acetyltransferase (Dlat) was confirmed as a direct target of HPF, with Dlat ablation significantly reducing HPF-induced adipose tissue browning, highlighting its potential as a therapeutic lead for obesity treatment. Farrerol, identified through ligand-based proteomics (LiP-MS) and validated by additional biochemical assays, directly targets ubiquitin C-terminal hydrolase L3 (UCHL3), enhancing its activity to promote RAD51 recombinase deubiquitination and improve homologous recombination repair. This mechanism restores genomic stability and significantly improves somatic cell nuclear transfer (SCNT) embryo development, providing a novel approach to enhance SCNT efficiency [61]. Scutellarin (SG), a flavonoid glucuronide from *Erigeron breviscapus*, has shown potential therapeutic effects on neurological diseases. Sheng et al. [62], combined LiP-MS with other validation methods, including molecular docking, co-immunoprecipitation (co-IP), DARTS, and CETSA, revealed that SG selectively inhibits pyruvate dehydrogenase kinase 2 (PDK2) activity, a key regulator of mitochondrial glucose oxidation, thereby protecting mitochondria from damage and improving mitochondrial aerobic respiration in cerebral ischemia.

#### 3.1.3 PELSA

Similar to DARTS and LiP-MS, PELSA is a limited-proteolysis method that utilizes a large amount of trypsin to produce peptides directly from native proteins [63]. Unlike DARTS and LiP-MS, which emphasize analyzing partially digested proteins and typically involve a second digestion step, PELSA identifies changes in peptide abundance associated with ligand-binding regions (Fig. 4C). Notably, PELSA identified 12-fold more kinase targets than LiP-Quant using multiple drug doses, and 2.4-fold more kinase targets than TPP using multiple temperatures, respectively [63]. PELSA emerges as a cutting-edge method with high sensitivity, attributed to its ability to identify targets with lower sequence coverage requirements, generate strong fold changes, and detect kinase targets with extreme melting temperatures, thereby surpassing the limitations of LiP-Quant and TPP [64]. However, it has not yet been applied to the screening of TCM, but holds significant potential for further research.

### 3.2 Denaturation resistance approach

#### 3.2.1 CETSA

**Fig. 5:**
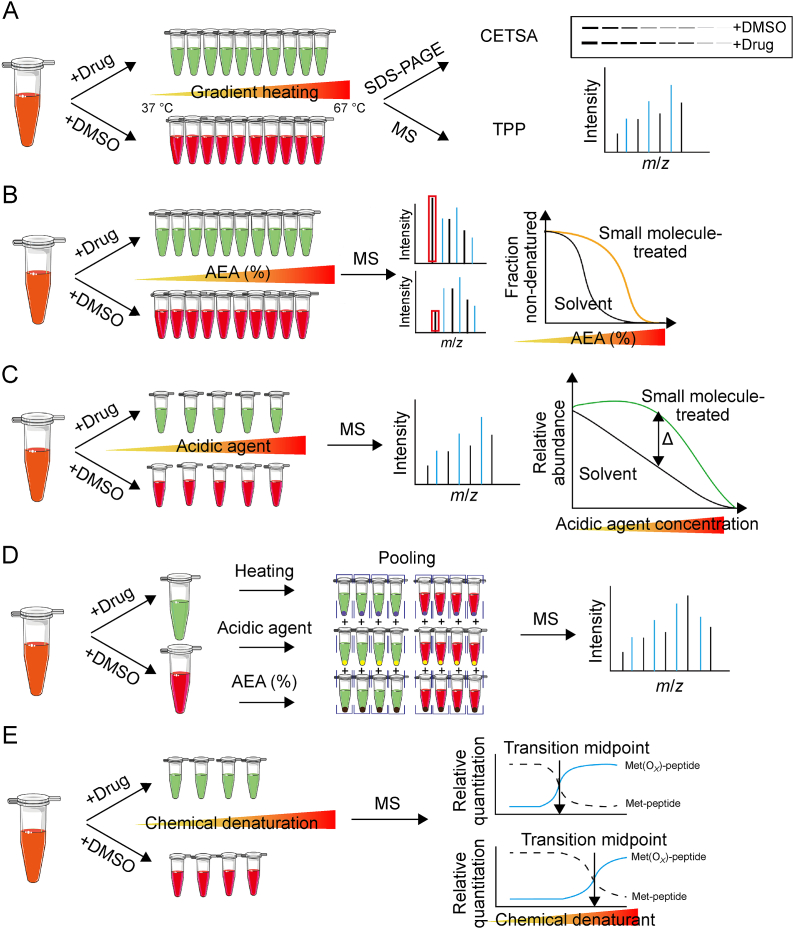
Workflow diagram of denaturation resistance approach for target identification: (A) thermal proteome profiling (TPP) and cellular thermal shift assay (CETSA), (B) solvent-induced protein precipitation (SIP), (C) pH-dependent protein precipitation (pHDPP), (D) integrated protein solubility shift assay (IPSSA), and (E) protein stability determinations from rates of oxidation (SPROX). SDS-PAGE: sodium dodecyl sulfate-polyacrylamide gel electrophoresis; MS: mass spectrometry; DMSO: dimethyl sulfoxide; AEA: acetone ethanol or acetic acid; Met: methionine.

The thermal stability of a protein can be altered by its interactions with biomolecules, forming the basis of the CETSA method [65] (Fig. 5A). The principle underlying CETSA technology is that the binding of a drug molecule changes the thermal stability of the target protein. In this assay, the cells treated with the target compounds of interest are collected, and proteins are extracted under native conditions, and then denatured by heating and precipitated. Monitoring the soluble protein fractions that remain after exposure to a range of temperatures generates melting profiles for each detected protein [66]. Unbound proteins undergo denaturation and precipitation in response to temperature increases, whereas more ligand-bound proteins remain in solution. CETSA is particularly valuable for confirming the target engagement of pharmacologically bioactive molecules [67]. Nevertheless, the usefulness of CETSA is limited to identifying unknown target proteins, and Western blot (WB)-based CETSA is generally used for target verification. The recent emergence of high-throughput CETSA, in combination with reporter luminescence and immunofluorescence detection, has increased the flux of CETSA data [68].

Currently, CETSA technology is extensively utilized in TCM due to its capability to provide quantitative data on target proteins in cell lysates, live cells, and tissues. A summary of applications in investigating therapeutic mechanisms is presented in Table 2 [[69], [70], [71], [72], [73], [74], [75], [76], [77], [78], [79], [80], [81], [82], [83], [84]]. For example, schisandrol A, derived from *Schisandra chinensis,* binds to the ATP6V0D1 subunit of the V-ATPase in PC12 cells, where it plays a role in preventing diabetic neuropathy [69]. Nordihydroguaiaretic acid inhibits histone scetyltransferase p300 and activates autophagy, extending lifespan in *Caenorhabditis elegans* (*C. elegans*) [70]. Wogonoside, shikonin, raddeanin A, and chamaejasmenin E exhibit effective anti-cancer properties [[71], [72], [73], [74]]. Calomembranone G and pentoxifylline target Toll-like receptor 4 (TLR4) to exert anti-inflammatory effects [75,76]. Moreover, eucalyptin C, derived from *Eucalyptus globulus* Labill., bings to phosphoinositide 3-kinases-γ (PI3Kγ), inhibiting the activation of primary spleen cells from allergic contact dermatitis mice [77].

**Table 2:** Denaturation resistance approach for target identification and validation of traditional Chinese medicines (TCMs).

Proteomic strategies	Compounds	Protein target	Cell model/animal model	Refs.
CETSA	Schisandrol A	ATP6V0D1	PC12 cells	[69]
	Nordihydroguaiaretic acid	P300	HEK293T cells	[70]
	Wogonoside	Gli1	MDA-MB-231 cells	[71]
	Shikonin	IMPDH2	MDA-MB-231 and 4T1 cells	[72]
	Raddeanin A	TDP-43	MC38 and B16 cells	[73]
	Chamaejasmenin E	Receptor tyrosine kinase c-Met	Hep3B cells	[74]
	Calomembranone G	TLR4	RAW264.7 cells	[75]
	Pentoxifylline	TLR4	RAW264.7 cells	[76]
	Eucalyptin C	PI3Kγ	Primary spleen cells	[77]
	Costunolide	CDK2	BV-2 cells	[78]
	Eupalinolide B	USP7	BV-2 cells	[79]
	Gentiopicroside	PAQR3	HEK 293T cells	[80]
	Ligustilide	CaMKII	VSMCs	[81]
	Proanthocyanidin A1	JAK2	Dami cells	[82]
	HDAC	RAN	AML12 cells	[83]
	Gambogic amide	WDR1	T2-4 and 293 cells	[84]
TPP	Artone	ASF1A	BV-2 cells	[87]
	Kurarinone	Soluble epoxide hydrolase enzyme	MPTP induced PD mice	[88]
	CNP	GPX4	U-2OS cells	[89]
	4,4′-Dimethoxychalcone	ALDH1A3	A549 cells	[90]
SIP	Sinomenine	GBP5	RAW264.7 cells	[92]
	Shikonin	NEMO/IKKβ	LoVo cells	[93]
pHDPP	Dihydroartemisinin	ALDH7A1 and HMGB1	HeLa cells	[94]

CETSA: cellular thermal shift assay; ATP6V0D1: a vacuolar-ATPase subunit; Gli1: glioma-associated oncogene 1; IMPDH2: inosine 5′-monophosphate dehydrogenase type II; TDP-43: transactive response (TAR)-DNA binding protein of 43 kDa; c-Met: mesenchymal-epithelial transition factor; TLR4: Toll-like receptor 4; PI3Kγ: phosphoinositide 3-kinases-γ; CDK2: cyclin-dependent kinase 2; USP7: ubiquitin-specific protease 7; PAQR3: progestin and adipoQ receptor 3; CaMKII: calcium-calmodulin-dependent protein kinase II; VSMCs: vascular smooth muscle cells; JAK2: Janus kinase 2; RAN: Ras-related nuclear protein; HDAC: hyodeoxycholic acid; WDR1: tryptophan-aspartic acid (WD) repeat domain 1; TPP: thermal proteome profiling; ASF1A: anti-silencing factor 1A; MPTP: 1-methyl-4-phenyl-1,2,3,6-tetrahydro pyridine; PD: Parkinson's disease; CNP: conophylline; GPX4: glutathione peroxidase 4; ALDH1A3: aldehyde dehydrogenase 1 family member A3; SIP: solvent-induced protein precipitation; GBP5: guanylate-binding protein 5; NEMO/IKKβ: nuclear factor-kappaB (NF-κB) essential modulator (NEMO)/IκB kinase β (IKKβ); pHDPP: pH-dependent protein precipitation; HMGB1: high mobility group box 1.

The combination of DARTS and CETSA has also been widely employed to validate biomolecules-protein interactions. For example, a combined DARTS and WB-CETSA strategy demonstrated costunolide, isolated from *Aucklandia lappa* Decne*,* targets cyclin-dependent kinase 2 (CDK2) in BV-2 cells [78]; whereas, eupalinolide B, extracted from *Eupatorium lindleyanum*, targets ubiquitin-specific protease 7 (USP7) in BV-2 cells [79]. Likewise, gentiopicroside, isolated from *Gentiana manshurica* Kitagawa, has been reported to directly bind to progestin and adipoQ receptor 3 (PAQR3), enhancing PAQR3 degradation via DNA-binding protein 2 (DDB2)-mediated ubiquitination, as confirmed by CETSA, SPR, and MST [80]. Ligustilide, a key active component of Suxiao Jiuxin pills widely used in cardiovascular disease treatment, covalently binds to calcium-calmodulin-dependent protein kinase II (CaMKII) at Cys116, inducing a long-lasting vasodilator effect. Through a combination of CETSA, proteomics, in-gel imaging, and molecular docking, it has been confirmed that the epoxidized metabolite of ligustilide effectively targets CaMKII in vascular smooth muscle cells, providing new insights into its potential for cardiovascular disease treatment [81]. In addition, proanthocyanidin A1 has been shown to directly bind to Janus kinase 2 (JAK2), as confirmed by isothermal dose-response fingerprint-CETSA, molecular docking, kinase activity, and SPR. This binding activates the JAK2/signal transducer and activator of transcription 3 (STAT3) pathway and ameliorates chemotherapy-induced thrombocytopenia [82]. Furthermore, hyodeoxycholic acid (HDAC), an active ingredient of TCM pig bile, has been emphasized its ability to ameliorate non-alcoholic fatty liver disease (NAFLD). In the study by Zhong et al. [83], a human protein microarray recognized Ras-related nuclear protein (RAN) as a direct target of HDAC, which was further validated through various techniques, including IP, proximity ligation assay (PLA), CETSA, and molecular docking. Bind of HDCA to RAN disrupts the RAN/chromosomal maintenance 1 (CRM1) shuttling complex, leading to increased nuclear accumulation of PPARα and enhanced fatty acid oxidation, providing a therapeutic mechanism for ameliorating NAFLD [83]. GA-amide, an analog of gambogic acid that constitutes a primary active constituent of the TCM, gamboge, has been found to directly bind WD repeat domain 1 (WDR1), by combining CETSA, DARTS, molecular docking simulation, and SPR, which inhibited glioma cell invasion and induced apoptosis [84].

CETSA enables direct biophysical studies in intact cells and validates target proteins identified by other methods but is unsuitable for unknown target discovery, affects cell membrane permeability, and has challenges with reproducibility and highly inhomogeneous proteins [[66], [67], [68]].

#### 3.2.2 TPP

TPP, an optimized CETSA, utilizes ligand-induced protein stabilization to deconvolute biomolecule targets in TCM (Fig. 5A). Specifically, ligand binding (e.g., biomolecule, nucleic acids, proteins, or post-translational modifications) alters the thermal stability of target protein, increasing its resistance to heat-induced unfolding and precipitation, and the ligand-protected stability shift is further quantified by MS to identify potential targets [85]. Both CETSA and TPP rely on the thermal stability of ligand-bound proteins; however, the MS-based TPP technology enables the high-throughput analysis of numerous proteins in profiling mode and has high efficiency for screening drug-binding target proteins. TPP requires no compound modification and can provide high-throughput identification of intracellular targets in living cells [86]. TPP has been successfully employed to identify drug targets and off-targets, as well as to study protein-metabolite and protein-protein interactions through its applicability *in vivo*, *in situ*, or *in vitro*. Consequently, TPP provides unique insights into protein states and interactions within their native context across the entire proteome, facilitating the investigation of fundamental biological processes and their underlying mechanisms.

Recently, TPP technology has been increasingly applied in TCM research, as depicted in Table 2 [[87], [88], [89], [90]]. TPP has confirmed that artone, isolated from the herb *Artemisia giraldii,* could directly target histone chaperone anti-silencing factor 1A (ASF1A) in BV-2 cells, significantly inhibiting neuroinflammation and inflammation-associated neurodegenerative diseases [87]. Kurarinone, derived from Sophorae Flavescentis Radix, has been demonstrated to target and inhibit the soluble epoxide hydrolase in Parkinson's disease mice [88]. Conophylline (CNP), a vinca alkaloid extracted from the *Tabernaemontana divaricata*, has been identified as a binding partner for glutathione peroxidase 4 (GPX4) using TPP, leading to lipid reactive oxygen species (ROS) accumulation and autophagy [89]. In addition, Yang et al. [90] employed an unbiased TPP method to identify multiple targets of flavonoid 4,4′-dimethoxychalcone, further validating aldehyde dehydrogenase 1 family member A3 (ALDH1A3) as a target for inhibiting A549 cells.

#### 3.2.3 SIP

The SIP approach is an energetics-based proteomics technique developed to investigate biomolecules-protein interactions in cell lysates by exploiting the increased resistance of biomolecules-binding protein to organic solvent-induced denaturation and precipitation (e.g., acetone, ethanol, or acetic acid) [91] (Fig. 5B). Further optimization is needed to do determine the appropriate organic solvent concentration (Table 2) [92,93]. For example, SIP screening has demonstrated that sinomenine directly targets guanylate-binding protein 5 (GBP5) to treat rheumatoid arthritis in lipopolysaccharide-stimulated RAW264.7 cells, a result further confirmed by CETSA validation [92]. Furthermore, combining SIP with TPP has revealed that shikonin targets the NF-κB essential modulator (NEMO)/IκB kinase β (IKKβ) complex, effectively inhibiting the growth of colorectal cancer cells [93]. SIP allows drug target identification without drug modification but lacks multiplexing for comprehensive proteome-wide analysis.

#### 3.2.4 pHDPP

pHDPP method assesses protein stability changes induced by ligand binding by treating proteins with an acidifying agent, causing them to gradually denature and precipitate, with ligand-binding proteins showing a shallower precipitation gradient compared to non-binding proteins, as measured by MS [94] (Fig. 5C). A critical factor is the controlling the concentration of the acidifying agent. The applications of this technique are shown in Table 2 [94]; for instance, pHDPP has identified dihydroartemisinin binding to aldehyde dehydrogenase aldehyde dehydrogenase 7 family member A1 (ALDH7A1) and HMGB1 in HeLa cells, and further validated by CETSA [94]. The combination of SIP and pHDPP technologies enhances high sensitivity in target protein screening. However, the cell membrane hinders the penetration of organic solvents, making this approach unsuitable for intact-cells experiments, as targets on the cell surface or structural changes after lysis are ignored. pHDPP works for multiple ligands with high sensitivity and complements other proteomic methods, though acidic agents may disrupt acid-base equilibrium in small molecule drugs.

#### 3.2.5 IPSSA

Nowadays, IPSSA has been developed to optimize workflows by integrating multiple assays, including pH shift assay, thermal shift assay, and solvent shift assay (Fig. 5D). The IPSSA approach analyzes ligand-induced proteins solubility changes, which improves sensitivity in target identification (up to 38%) compared to individual methods [95]. Staurosporine, a pan-kinase inhibitor, validated the IPSSA approach, highlighting its ability to detect drug targets with increased statistical power and fewer false positives [95]. Although, IPSSA technology has not yet been applied in TCM, it demonstrates strong potential in highly sensitive target exploration capabilities.

#### 3.2.6 SPROX

SPROX assesses peptide-level thermodynamic stability of proteins and their complexes. SPROX employs quantitative shotgun proteomics with hydrogen peroxide-mediated methionine labeling and measures the chemical denaturant dependence of this oxidation to reveal global protein stability [96,97] (Fig. 5E). However, SPROX is limited in detection methionine oxidation rate in peptide segments, which are relatively rare in protein sequences. Moreover, SPROX requires higher concentrations of drugs (μmol/L to mmol/L) [98,99], which poses challenges in obtaining sufficient amounts of TCM components. Therefore, the application of SPROX in investigating the active mechanisms of TCM has been limited. SPROX could detect temperature and enzyme-insensitive proteins but requires higher compound concentrations and only identifies proteins susceptible to selective methionine oxidation.

### 3.3 Chemical probe approach

#### 3.3.1 ABPP

**Fig. 6:**
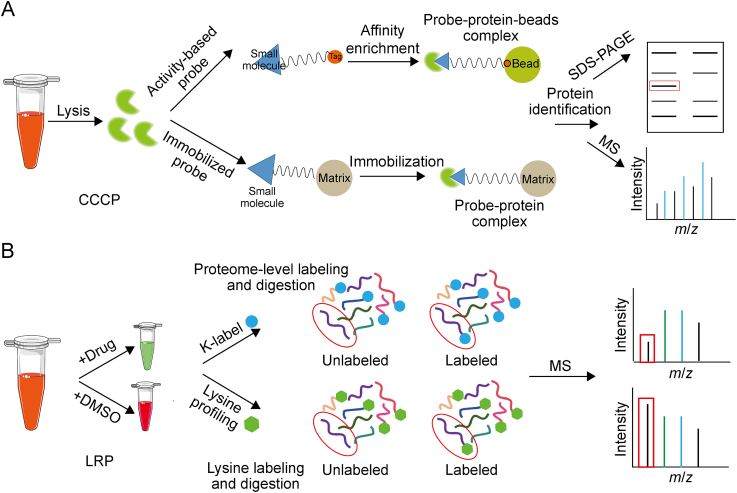
Workflow diagram of chemical probe approach for target identification: (A) activity-based protein profiling (ABPP) and compound-centric chemical proteomics (CCCP) and (B) target responsive accessibility profiling (TRAP) and lysine reactivity profiling (LRP). SDS-PAGE: sodium dodecyl sulfate-polyacrylamide gel electrophoresis; MS: mass spectrometry; DMSO: dimethyl sulfoxide.

ABPP utilizes activity-based probes (ABPs) to detect enzyme activity or amino acid reactivity in complex biological systems, where the pharmacological activity of ABPs allows for efficient enrichment and identification of binding protein targets, which is pivotal for robust proteome-wide target identification [100,101]. Moreover, ABPP has matured into a standard technology for the rapid, sensitive, and selective profiling of enzyme activity and inhibitors in proteomes, especially useful when compound-specific probes are unavailable or display weak target affinity, which is particularly suitable for TCM with low abundance of the active ingredient [102,103] (Fig. 6A). However, limitations of ABPP include the finite number of molecules that can be chemically modified without altering interactions with the target protein, potentially resulting in false positives.

Extensive evidence summarized in Table 3 [[104], [105], [106], [107], [108], [109], [110], [111], [112], [113]]. For instance, cucurbitacin B has been shown to bind to GRP78, inhibiting the GRP78/forkhead box M1 (FOXM1)/kinesin family member 20 A (KIF20A) pathway, thereby suppressing the proliferation of conjunctival melanoma cells [104]. Moreover, ABPP has demonstrated that daphnane diterpenoid, isolated from the *Daphne genkwa*, directly targets importin-β1 in prostate cancer cells. This interaction was subsequently confirmed by WB-CETSA, supporting the potential of daphnane diterpenoid as a promising therapeutic agent for castration-resistant prostate cancer [105]. In addition, a combination of ABPP and WB-CETSA technology revealed that celastrol directly targets peroxiredoxins (PRDX) to ameliorate hepatic fibrosis in LX-2 cells [106]. With affinity-based protein profiling, withangulatin A (WA) has been identified as a novel covalent inhibitor of phosphoglycerate dehydrogenase (PHGDH), directly binding to Cys295 and inactivating its enzyme activity, which was further validated by BIL and LC-MS/MS, demonstrating selective binding to PHGDH, blocking its substrate-binding domain, and inhibiting tumor proliferation [107]. Similarly, through ABPP, WA was identified as a novel covalent inhibitor of peroxiredoxin 6 (PRDX6). Further validation using CETSA, DARTS, and BLI assays confirmed that WA binds specifically to the cysteine 47 residue, inhibiting both its GPX and phospholipase A2 activities, suggesting WA as a potential anti-tumor agent [108]. Besides, ABPP revealed that parthenolide (PN) preferentially modified specific targets, with proteomics analysis further highlighting its role in the ubiquitin-mediated proteolysis pathway. Subsequent validation confirmed that PN covalently modified ubiquitin carboxyl-terminal hydrolase 10 (USP10) at Cys40, inhibiting breast cancer cell proliferation through LC-MS/MS, USP10 knockdown, and deubiquitinating enzyme activity assays, consistent with our ABPP findings [109]. Furthermore, diacylglycerol kinase family member DGKQ was identified as a direct target of the phytochemical atractylenolide II (AT II) through ABPP mapping, with further validation using CETSA, DARTS, SPR, and MST. AT II acts on a novel drug-binding pocket in the CRD and PH domains of DGKQ, allosterically regulating its kinase activity, which in turn improves obesity-induced insulin resistance and hyperlipidemia [110]. Chromatin assembly factor 1 subunit (CHAF1B) has been identified as key target of puerarin in protecting cardiomyocytes from apoptosis under high glucose through ABPP, CETSA, and knockdown [111]. Capsaicin (CAP) has been identified as a direct binder of pyruvate kinase M2 (PKM2) and lactate dehydrogenase A (LDHA) through streamlined cysteine-ABPP, CETSA-WB, SPR, and pull-down assays, offering promising therapeutic strategies for sepsis and inflammation [112]. ABPP identified proliferating cell nuclear antigen (PCNA) as a potential target of BVC, with the interaction confirmed by CETSA, DARTS, and SPR, which elucidates that bavachinin exert hepatoprotective effects in NAFLD [113]. Additionally, photo-affinity labeling-ABPP has demonstrated that 3-*O*-*trans*-p-coumaroyl maslinic acid (OCMA) isolated from *Ligustrum lucidum* Ait. binds to the S1 subsite of γ-secretase [114]. The advancements of ABPP underscore versatility in identifying targets and mechanisms in complex biological systems. ABPP enables rapid, sensitive, and selective detection of enzyme activity and inhibitors but struggles with low-abundance proteins, requires probe synthesis that can alter drug activity, and necessitates chemical modification of compounds.

**Table 3:** Chemical reactivity approach for target identification and validation of traditional Chinese medicines (TCMs).

Proteomic strategies	Compounds	Protein Target	Cell model/animal model	Refs.
ABPP	Cucurbitacin B	GRP78	Conjunctival melanoma cells	[104]
	Daphnane diterpenoid DD1	Importin-β1	CRPC cells	[105]
	Celastrol	PRDXs	LX-2 cells	[106]
	WA	PHGDH	HCT-116 cells	[107]
	WA	PRDX6	H1975 cells	[108]
	PN	USP10	MDA-MB-231 cells	[109]
	AT II	DGKQ	HepG2 cells	[110]
	Puerarin	CHAF1B	AC16 cell line	[111]
	CAP	PKM2 and LDHA	RAW264.7 cells	[112]
	Bavachinin	PCNA	HepG2 cells	[113]
CCCP	Artemisinin	Gephyrin protein	MIN6 cells	[118]
	Curcumol	Nucleolin	NPC cells	[119]
TRAP	Cycloastragenol	CTSB	MC38 cells	[121]
	Celastrol	CAP1	THP-1 cells	[122]
	Silibinin	ACSL4	HepG2 cells	[123]
	Lobeline	MAPK14	MC38 cells	[124]

ABPP: activity-based protein profiling; GRP78: glucose regulated protein 78; CRPC: castration-resistant prostate cancer; PRDXs: peroxiredoxins; WA: withangulatin A; PHGDH: phosphoglycerate dehydrogenase; PRDX6: peroxiredoxin 6; PN: parthenolide; USP10: ubiquitin carboxyl-terminal hydrolase 10; AT II: atractylenolide II; DGKQ: diacylglycerol kinase theta; CHAF1B: chromatin assembly factor 1 subunit; CAP: capsaicin; PKM2: pyruvate kinase M2; LDHA: lactate dehydrogenase A; PCNA: proliferating cell nuclear antigen; CCCP: compound-centered chemical proteomics; NPC: nasopharyngeal carcinoma; TRAP: target responsive accessibility profiling; CTSB: cathepsin B; CAP1: adenylyl cyclase associated protein 1; ACSL4: ayl-CoA synthetase long chain family member 4; MAPK14: mitogen-activated protein kinase 14.

#### 3.3.2 CCCP

The CCCP technique is a simple and chromatography based approach that involves covalent immobilization of small molecule (whose bioactivity is typically known) onto a solid matrix, such as agarose, followed by incubation with a protein lysate to capture the interacting proteins [115,116] (Fig. 6A). Compared to ABPP, CCCP disabled to identify the activation state of target proteins but offers a more unbiased approach, even enabling the identification of no enzymatic targets, thereby facilitating the discovery novel targets [117]. In recent years, CCCP technology has successfully identified small molecule targets in TCM, as summarized in Table 3 [118,119]. It has revealed that artemisinin, an active compound, targets the gephyrin protein in MIN6 cells, which is responsible for the regeneration of pancreatic β cell mass from α cells in the treatment of type 1 diabetes [118]. Curcumol, isolated from Rhizoma Curcumae, directly targets the nucleolin protein innasopharyngeal carcinoma cells [119]. CCCP enhances selectivity and identifies non-enzymatic targets, though nonspecific binding increases false positives and immobilizing bioactive compounds is challenging.

#### 3.3.3 TRAP

TRAP is an emerging chemoproteomics target discovery approach that measures protein changes induced by ligand binding to global lysines [120] (Fig. 6B). Unlike classical probe-based chemoproteomics, TRAP avoids the need for derivatization, allowing high-coverage and high-throughput identification of the targets of multiple drugs, making it particularly promising for TCM target discovery. Recent applications of TRAP in TCM target discovery are detailed in Table 3 [[121], [122], [123], [124]]. For instance, the combination of TRAP and CETSA has been used to show that the antitumor immunity and mechanism of cycloastragenol involve a reduction in the degradation of major histocompatibility complex class I (MHC-I) in MC38 cells via directly targeting cathepsin B (CTSB) [121]. Besides, TRAP has demonstrated that celastrol binds adenylyl cyclase associated protein 1 (CAP1) in THP-1 cells to inhibit resistin-induced inflammation, as confirmed by CETSA [122]. TRAP can also applied in living cells; for example, in combination with thermal shift assays and DARTS, TRAP has validated that silibinin targets acyl-CoA synthetase long chain family member 4 (ACSL4) in living HepG2 lysates to protect from ferroptosis [123]. Lobeline, an alkaloid from the herbal medicine lobelia, could remodel the immunosuppressive microenvironment. Using TRAP, 16 potential target proteins were identified, and further confirmed the binding lobeline and MAPK14 through ITC, molecular docking, and site-directed mutagenesis [124]. TRAP eliminates the need for synthesizing photo-affinity probes and allows target deconvolution of metabolites, but it requires protein lysine labeling and may produce false positives.

#### 3.3.4 LRP

The LRP strategy, based on active dimethyl labeling, has been developed to for mass spectrophotometric detection of the patterns of conformation modulation due to molecule ligand binding to protein targets [125,126] (Fig. 6B). This approach facilitates the discovery of biomolecule targets by tracking conformational changes, such as hydrogen bond formation or electrostatic interactions at lysines in protein-protein interaction interface [127]. However, the detection sensitivity of conformational changes at the proteome level is limited to the sensitivity. To date, few studies have been reported on the identification of TCM targets using LRP strategies.

### 3.4 Native MS

**Fig. 7:**
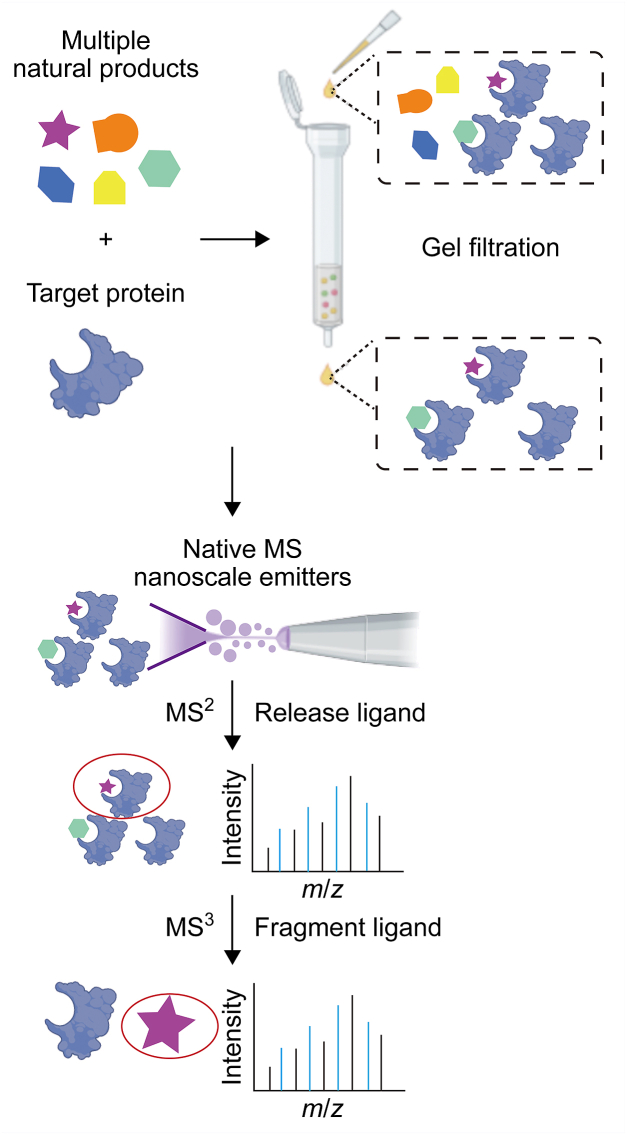
Workflow diagram of native-mass spectrometry (MS) for target identification. Created by using BioRender.com.

Native MS offers direct investigation of protein-ligand interactions under non-denaturing conditions to detect non-covalent and covalent complexes, enabling the identification of ligands through mass-to-charge ratio shifts and precise molecular weight calculations from the mass differences between unbound and bound proteins [16] (Fig. 7).

Native MS screened the binding ligands to severe acute respiratory syndrome coronavirus 2 non-structural protein 9 (SARS-CoV-2 Nsp9) in the 1614 natural product library, which oridonin has the strongest binding affinity to Nsp9 [128]. Quinn and colleagues [129] have developed a fragment-based approach to investigate potential targets for antimalarial. They have identified 96 natural products as binding partners of 32 putative malarial targets, with 79 of these compounds showing *in vitro* antimalarial activity [129]. Moreover, an integrated untargeted metabolomics-native MS workflow [130] has been applied to high-throughput screening against human carbonic anhydrase I (hCAI) from crude nature product extracts. Following low-volume gel filtration, native MS coupled with nanoscale ion emitters enabled direct screening of intact protein-biomolecule complexes from complex natural product extracts. Subsequently, biomolecules were further dissociated and identified via multistage MS (MS^*n*^). Screening against three targets (bovine carbonic anhydrase II, human carbonic anhydrase VII (CAVII), and lysozyme) revealed 14 distinct biomolecules. Native MS enables direct identification of binding sites in the crude extracts without requiring protein digestion or labeling.

## 4 Summary and prospects

**Fig. 8:**
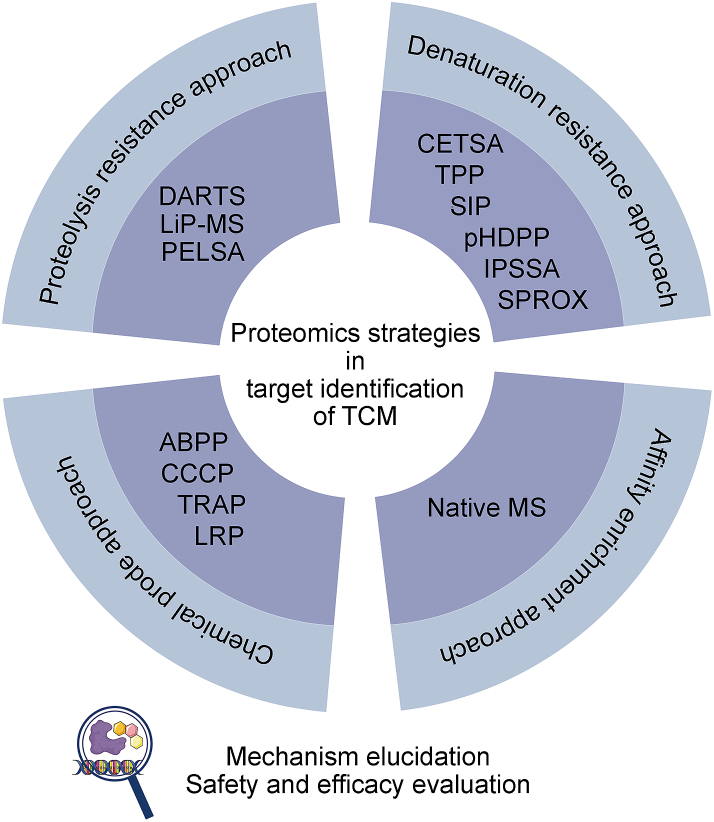
Proteomics techniques have emerged as a cornerstone in early-stage drug discovery. DARTS: drug affinity responsive target stability; LiP-MS: limited proteolysis-mass spectrometry; PELSA: peptide-centric local stability assay; CETSA: cellular thermal shift assay; TPP: thermal proteome profiling; SIP: solvent-induced protein precipitation; pHDPP: pH-dependent protein precipitation; IPSSA: integrated protein solubility shift assay; SPROX: protein stability determinations from rates of oxidation; TCM: traditional Chinese medicine; ABPP: activity-based protein profiling; CCCP: compound-centric chemical proteomics; TRAP: target responsive accessibility profiling; LRP: lysine reactivity profiling.

Proteomic techniques have emerged as a cornerstone in early-stage drug discovery (Fig. 8), offering powerful tools for target identification, validation, and evaluation of safety and efficacy. In the field of TCM, proteomic techniques are revolutionizing the discovery of molecular targets, shedding light on the complex interactions between bioactive compounds and their biological pathways. This review delves into recent advances in proteomics application for TCM target discovery and highlights the directions to harness their full potential.

Recent strides in proteomics have enabled significant progress in characterizing and identifying the molecular targets of TCM, which have illuminated the multifaceted and mechanisms underlying therapeutic effects and pharmacological basis of TCM. However, despite these achievements, current proteomics strategies still face challenges. Each technique has distinct strengths and limitations regarding sensitivity, resolution, and scalability (Table 4) [16,39,54,[56], [57], [58],^63^,[66], [67], [68],^85^,^86^,^91^,[94], [95], [96], [97], [98], [99],[101], [102], [103],[115], [116], [117],^120^,^126^,^130^]. More research technologies and better application of currently available proteomic strategies are needed to tap the full drug potential of TCM. Among the emerging technologies, hydrogen deuterium exchange MS (HDX-MS) stands out for its high sensitivity in probing the conformational dynamics of protein molecule interactions [131]. As HDX-MS gains traction, its application in TCM research is poised to bridge critical gaps in target validation and mechanism elucidation. Currently, no single technique provides a comprehensive, “one-stop shop” solution for target identification [132]. The different proteomic strategies available for identifying TCM targets are strongly complementary. Hence, integrating different proteomics approaches and platforms represents a critical opportunity to overcome individual limitations and achieves a more holistic understanding of molecular interactions.

**Table 4:** Advantages and limitations between different approaches for target identification.

Approach	Advantages	Limitations/disadvantages	Refs.
DARTS	i) Without chemical structural modification of biomolecule ii) Simpler operation and higher throughput iii) Suitable for those drugs causing target protein degradation	i) Not applicable for the drugs with structure change after entering cells ii) Limits on the detection of low abundance target protein due to the low recovery of peptides from gel digestion iii) Limits on those target proteins difficult to digest by protease	[39,54]
Lip-MS	Without chemical structural modification of the biomolecule	i) Limits on the detection of low abundance target proteins ii) Limits on the target proteins without proper MS detectable peptides in the binding sites iii) Limits on the target proteins with conformational changes that hamper binding site identification	[[56], [57], [58]]
PELSA	i) Higher sensitivity ii) Without chemical structural modification of the biomolecule iii) Direct ligand binding-site determination iv) Ligand-induced local stability shifts	i) Need amount of trypsin ii) Higher cost	[63]
CETSA	i) Direct biophysical studies in intact cells ii) Suitable for the validation of target proteins identified by other methods	i) Not applicable for unknown target protein discovery ii) Affect permeability of cell membrane iii) The melting curve cannot be reproduced iv) Not suitable for highly inhomogenous proteins v) Not recognize the binding site	[[66], [67], [68]]
TPP	i) Without chemical structural modification of biomolecule ii) Suitable for the drugs with structure change after entering cells	i) Higher cost ii) Low transmembrane protein extraction	[85,86]
SIP	Without chemical structural modification of the biomolecule	Not adopt sample multiplexing for quantification of complete melting curves on a proteome-wide scale	[91]
pHDPP	i) Work for multiple ligands ii) High sensitivity iii) High complementarity to other proteomic approaches	Acidic agent may shift the acid-base equilibrium for some small molecule drugs	[94]
IPSSA	i) Without chemical structural modification of biomolecule ii) Easy operationation	i) Limits on the detection of low abundance target protein ii) Unable to distinguish direct/indirect effects	[95]
SPROX	Detecting temperature and enzyme insensitive proteins	i) Needing higher concentration of compound treatment ii) Detect only the target proteins of highly selective methionine oxidation reaction	[[96], [97], [98], [99]]
ABPP	i) Identifying the activation state of target proteins ii) Achieving the fast, sensitive, and selective identification of enzyme activity and inhibitors	i) Low detection sensitivity for low abundance protein targets ii) Probe synthesis affects pharmacological activities of drugs iii) Requiring chemical modification of compounds	[[101], [102], [103]]
CCCP	i) Higher selectivity to reduce the diversity of target protein complex ii) Identifying targets with no enzymatic function	i) Nonspecifically binding proteins lead to potential false-positive results ii) Not easy to immobilize bioactive compounds onto the matrix	[[115], [116], [117]]
TRAP	i) Requiring no synthesizing photo-affinity probes ii) The metabolite of interest for target deconvolution is feasible	i) Protein lysine needs to be labeled ii) The presence of the false-positive results	[120]
LRP	Without chemical structural modification of biomolecules	The lysine reactivity based on the native microenvironments of lysine local structures	[126]
Native-MS	i) Non denaturing condition ii) Tracing sample and fast process	i) Low detection sensitivity for low abundance protein targets or weakly bound complexes ii) High sample purity, avoiding interference from salts or detergents on MS signals	[16,130]

DARTS: drug affinity responsive target stability; LiP-MS: limited proteolysis-mass spectrometry; PELSA: peptide-centric local stability assay; CETSA: cellular thermal shift assay; TPP: thermal proteome profiling; SIP: solvent-induced protein precipitation; pHDPP: pH-dependent protein precipitation; IPSSA: integrated protein solubility shift assay; SPROX: protein stability determination from rates of oxidation; ABPP: activity-based protein profiling; CCCP: compound-centered chemical proteomics; TRAP: target responsive accessibility profiling; LRP: lysine reactivity profiling.

The complexity of TCM components is primarily reflected in the synergistic effects of multiple components and the complexity of metabolites. Orally administered small molecules from TCM often undergo phase I/II metabolism, generating bioactive derivatives that may diverge mechanistically from their parent compounds, which may affect the identification of direct targets in proteomics. For instance, dihydrocaffeic acid (DA), a primary circulating metabolite of chlorogenic acid, demonstrates potent anti-inflammatory activity in systemic disorders. DA mediates protective effects against acute pneumonia by targeting transaldolase 1 (TALDO1) at Cys250 through multi-proteomic strategies (ABPP and CETSA), which elucidates its anti-inflammatory efficacy *in vivo* and *in vitro* [133]. On the other hand, multi-components of TCM may affect multiple target proteins and pathways through synergistic actions, making it difficult to elucidate the mechanism. Wang and co-workers [134] has demonstrated that the natural isomeric formulation of *Curcuma wenyujin* (85% β-elemene, 15% γ/δ-elemene) exhibits superior antitumor efficacy and safety compared to purified β-elemene alone. This finding validates the synergistic advantage in TCM, which multi-component compatibility outperforms single component paradigms [134]. To address the challenges, advanced proteomic strategies to deconvolute multi-component mixtures have enabled the discovery of promising therapeutic leads with diverse pharmacological activities. Native MS integrated with online fractionation enables high-throughput characterization of endogenous protein complexes, directly capturing ligand, particularly when combined with MS^*n*^, holds significant potential for mapping dynamic protein interactions, identifying unknown ligands, and elucidating functional relationships within metabolic pathways on a global scale [135].

This paper has reviewed and highlighted critical advances in proteomics-driven target discovery for TCM, critically evaluating their respective strengths, disadvantages, and applicability. While these approaches have been widely used *in vitro* target discovery, their translation to *in vivo* and clinical applications remains constrained by technical challenges. A primary limitation arises from the complexity of *in vivo* systems, where high-abundance proteins (e.g., albumin and immunoglobulins) obscure low-abundance targets, reducing detection sensitivity. Furthermore, small molecule pharmacokinetics introduce unpredictability *in vivo*. In addition, current target proteomics have predominantly relied on acute extracellular or intracellular drug exposure to identify direct targets. In clinical therapies, prolonged small molecules often involve dynamic remodeling of downstream signaling cascades and regulatory networks, obscuring relationships between target binding and clinical outcomes. Certainly, clinical trials remain pivotal for efficacy evaluation, and proteomics has demonstrated promise in identifying diagnostic and prognostic biomarkers. For instance, the analysis of serum proteome changes in psoriasis patients treated with Yinxieling revealed elevated ficolin2, macrophage migration inhibitor factor, and matrix metallopetidase 1 levels as potential biomarkers correlating with therapeutic response [136].

Proteomic strategies have provided feasible options for identifying active compounds that can serve as biological probes and new drug candidates. Looking ahead, the value of identifying and targeting active ingredients in TCM will drive more precise and evidence-based practices in its clinical use. The current analysis predominantly focuses on proteomic-based target identification methodologies, with insufficient attention to validation strategies such as Försters resonance energy transfer (FRET), ITC, SPR, SwitchSense, and MST. As proteomics evolves, its integration with complementary technologies such as single-cell proteomics, multi-omics integration (transcriptomics, metabolomics, and proteomics), artificial intelligence (AI)-driven target discovery, machine learning (ML)-driven prediction, and systems biology will further enhance its capacity to unravel the complexities of TCM.

In conclusion, while current proteomic approaches have transformed TCM target discovery, their potential remains largely untapped. Continued refinement of existing methods and the development of innovative technologies will be instrumental in addressing the challenges posed by TCM's complexity. By advancing proteomic strategies and fostering interdisciplinary collaborations, researchers can fully harness the therapeutic potential of TCM, ultimately enriching global healthcare with its time-honored wisdom and evidence-based efficacy.

## 5 Conclusion

Proteomics has emerged as a transformative force in elucidating the molecular targets of TCM. While current techniques provide powerful tools for target deconvolution and mechanistic insights, technical limitations persist. This review integrates pivotal advances and critically evaluates proteomics strategies for target deconvolution in TCM.

## CRediT authorship contribution statement

**Xueyan Zhen:** Writing – original draft, Investigation. **Jingwen Liu:** Writing – review & editing, Validation, Supervision, Funding acquisition, Conceptualization. **Yan Ren:** Validation, Supervision, Funding acquisition, Conceptualization.

## Declaration of competing interest

The authors declare that they have no known competing financial interests or personal relationships that could have appeared to influence the work reported in this paper.
