# Supplementary material for: Recent advances in proteomic strategies for target identification of traditional Chinese medicine
Source: J Pharm Anal. 2025 Dec 10;16(7):101516. doi: 10.1016/j.jpha.2025.101516 (PMC13377450; doi:10.1016/j.jpha.2025.101516)
Supplement: Multimedia component 1 [file mmc1.pdf]

PAPER NAME

Recent Advances in Proteomic Strategies for Drug Target Identification of Traditional Chinese Medicine

AUTHOR

-

WORD COUNT

15828 Words

CHARACTER COUNT

90476 Characters

PAGE COUNT

61 Pages

FILE SIZE

3.8MB

SUBMISSION DATE

May 17, 2025 12:56 PM GMT+8

REPORT DATE

May 17, 2025 12:58 PM GMT+8

17% Overall Similarity

The combined total of all matches, including overlapping sources, for each database.

- 11% Internet database
- 16% Publications database
- Crossref database
- Crossref Posted Content database
- 0% Submitted Works database

Excluded from Similarity Report

- Bibliographic material

Journal of Pharmaceutical Analysis

Recent Advances in Proteomic Strategies for Drug Target Identification of Traditional Chinese Medicine

--Manuscript Draft--

**Manuscript Number:**	JPA-D-25-00412R1
**Article Type:**	Review paper
**Section/Category:**	Natural products and traditional Chinese medicine
**Keywords:**	Proteomics Strategies; Target Protein Identification; traditional chinese medicine
**Abstract:**	Traditional Chinese medicine (TCM) has played an indispensable role in health intervention and disease treatment. Identifying the target proteins of TCM is crucial to clarifying therapeutic mechanisms. One approach taken to enhance the breadth, depth, and precision of studies on the active cellular pathways induced by TCM has been to use proteomics to reveal potential drug targets with direct interactions. Proteomic strategies facilitate identifying and characterizing target binding proteins relevant metabolism pathways, which involves enriching the complex of small molecule and their targets based on the affinity, as well as utilizing the changes in physicochemical properties of target proteins that occur due to drug binding for proteomic identification and quantification. Probe labeling and enrichment technologies have accelerated the field of chemical proteomics. Technologies focused on measuring the changes in target proteins have been widely extended into several different approaches, and these now drive the establishment of further strategies. This review summarizes the advances in proteomics strategies mainly based on mass spectrometry for the identification of TCM targets to the present. Enhancing the application of proteomics would provide a new viewpoint on TCM treatment, while underscores the potential of TCM as biological probes and sources of novel drug candidates.

Powered by Editorial Manager® and ProduXion Manager® from Aries Systems Corporation

Dear Editors and Reviewers:

Thanks for your information and the comments concerning our manuscript entitled “**Recent Advances in Proteomic Strategies for Target Identification of Traditional Chinese Medicine**” **(Manuscript Number: JPA-D-25-00412)**. We are grateful to your constructive comments and suggestions and have meticulously addressed each comment and accordingly revised the manuscript. Modifications in the manuscript are marked in red and yellow, and responses to review comments are highlighted with “answer” in green. We would greatly appreciate your favorable consideration of our revised submission. Enclosed below this letter are our point-by-point responses to the reviewers' comments.

Sincerely yours,

Yan Ren Ph.D

Experiment Center for Science and Technology, Shanghai University of Traditional Chinese Medicine, Shanghai, P. R. China.

Reviewer #1:

This review focuses on the recent advances in the application of proteomic strategies for protein target identification and validation of TCM components. It is a relatively comprehensive review. Followings are some suggestions for revision.

1. The Highlights should be revised according to the requirement of Elsevier. https://www.elsevier.com/researcher/author/tools-and-resources/highlights.

Answer: Thanks for the good reminding. We have revised the Highlights to summary the key points of this review for audience reference and labeled it with red and yellow.

2. The improvement of English language is highly desirable. Please avoid using the first-person "we" narratives.

Answer: Thank you for the carefully reviewing. The whole manuscript has been polished by a professional English editor and the certificate is attached as following. The using of the first-person "we" has been revised in Line 17 of Page 3, which is labeled in red and yellow.

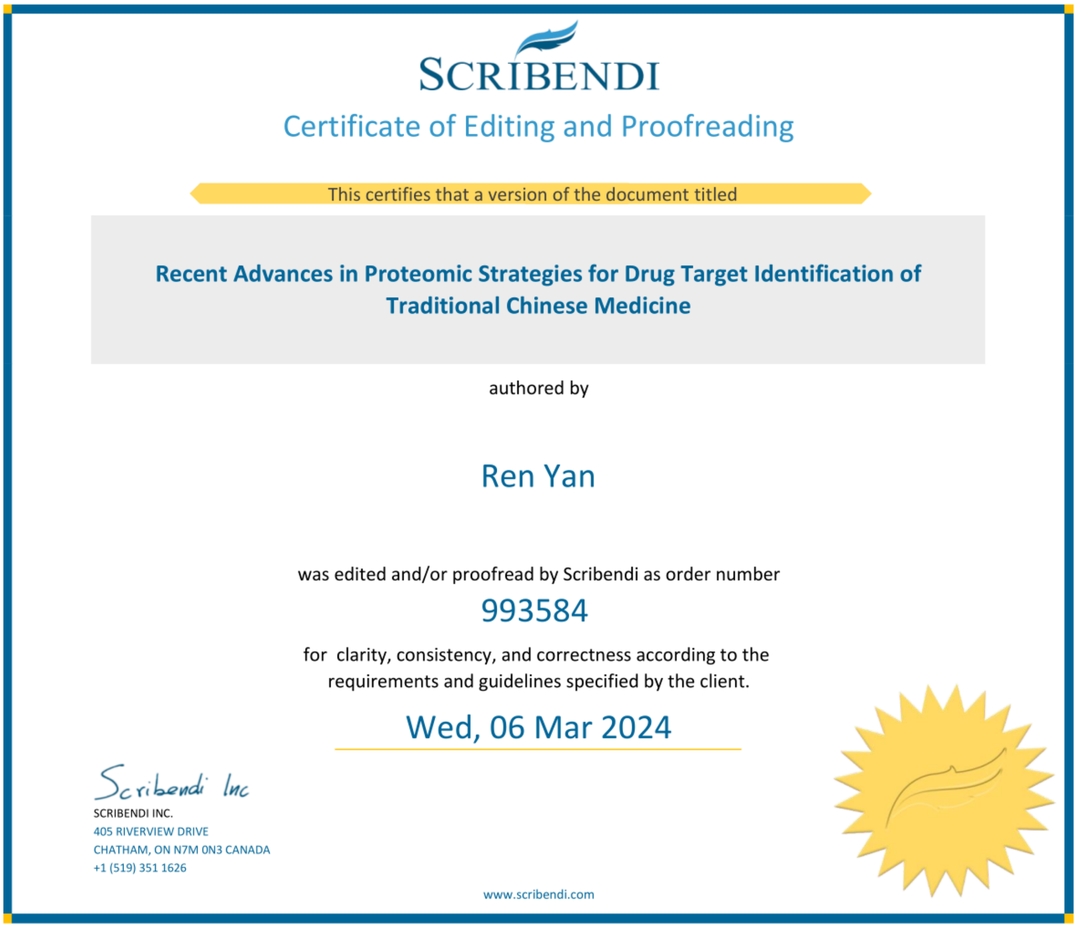

SCRIBENDI

Certificate of Editing and Proofreading

This certifies that a version of the document titled

Recent Advances in Proteomic Strategies for Drug Target Identification of Traditional Chinese Medicine

authored by

Ren Yan

was edited and/or proofread by Scribendi as order number

993584

for clarity, consistency, and correctness according to the requirements and guidelines specified by the client.

Wed, 06 Mar 2024

Scribendi Inc

SCRIBENDI INC.
405 RIVERVIEW DRIVE
CHATHAM, ON N7M 0N3 CANADA
+1 (519) 351 1626

www.scribendi.com

3. The Graphical Abstract (GA) can be improved. The authors can modify the GA according to the suggestions of Elsevier. https://www.elsevier.com/researcher/author/tools-and-resources/graphical-abstract.

Answer: Thank you for the careful attention. We have revised the Graphical Abstract according to

the suggestions of Elsevier.

4. In the last paragraph of introduction section, the significance, content, and purpose of present review can be emphasized.
Answer: Thanks for your wonderful suggestions, which are important to improve our manuscript. We have emphasized the significance, content, and purpose of this review on Page 3 Line 17-23 as you suggested.

5. It is suggested to provide more figures about the research results from the research papers, as most of the currently provided figures are the "Workflow diagram".
Answer: Thanks for this wonderful suggestion. We have supplemented Figure 3 in the section of “Challenges and opportunities for the discovery of TCM target proteins” to show the research result of Tu's team on Page 4 Line 22-32. This figure illustrates their innovative TCM-MPs strategy for self-assembly of active small molecules and bio-layer interferometry-based reverse screening, as applied to Shenqi Jiangtang Granules, highlighting its utility in target discovery within complex systems.

6. Please check the abbreviations, the abbreviations that have been defined in the introduction section, do not need to be re-defined in the following text.
Answer: Thanks for the carefully reviewing and nice suggestion. We have checked the whole manuscript and revised those redundant defining.

7. The abbreviations should be removed from the subtitle, such as "(TPP)".
Answer: Thanks for the carefully reviewing and good suggestion. We have checked the whole manuscript and removed the abbreviations which are shown in the subtitles.

8. Please carefully check the Latin names of plant and crude drug, for example, the crude drug names should not be in Italics font, such as "Rhizoma Menispermi".
Answer: Sorry for the mistake and thanks for your good suggestion. We have checked the entire manuscript and revised the Latin names of crude drugs on Page 11 Line 29, Page 15 Line 16, Page 15 Line 34.

9. It is suggested to provide a separated conclusion section, and the limitations of this review can be emphasized in the conclusion section.
Answer: Thanks for the insightful suggestion which is very helpful for the manuscript. The limitations of this review have been emphasized in the conclusion section from Page 19 Line 30 to Page 20 Line 6.

Reviewer #2:

This manuscript provides a systematic review of recent advances in proteomics strategies for target identification in traditional Chinese medicine (TCM), and the selected topic has significant academic value and application potential. The manuscript innovatively organizes the application of multiple proteomics technologies (e.g., DARTS, LiP-MS, CETSA, TPP, etc.) in the multi-component and multi-target research of TCM, and emphasizes the importance of technological integration and complementation. The authors propose the prospective view of combining proteomics with artificial intelligence and systems biology, which provides new ideas for modernizing TCM research. In addition, several TCM component target validation cases cited in the manuscript demonstrate the unique advantages of proteomics in analyzing the complex TCM mechanisms

Here are some suggestions and comments

1. Some sections, such as IPSSA, SPROX, LRP are slightly abbreviated, and the logical connection would be tighter if the discussion of the relevance of technical principles to TCM applications could be added. As well as in the use cases in TCM, some of the technologies lack examples or discussion of their application in TCM.

Answer: Thanks for the carefully reviewing and good suggestions. While IPSSA, SPROX, and LRP have seen limited direct application in TCM to date, their recent successes in characterizing small molecule-protein interactions demonstrate strong translational potential.

2. Insufficient analysis of how TCM component complexity affects proteomics results, not just the compounds themselves, such as synergistic effects, metabolites, etc., suggests adding relevant applications and discussion.

Answer: Thanks for your insightful suggestion and careful review. We have added it in the conclusion section from Page 18 Line 20 to Page 19 Line 12 and labeled it with red and yellow. Indeed, the complexity of TCM components is primarily reflected in the synergistic effects of multiple components and the complexity of metabolites. Orally administered small molecules from TCM often undergo phase I/II metabolism, generating bioactive derivatives that may diverge mechanistically from their parent compounds, which may affect the identification of direct targets in proteomics. For instance, Ginsenosides and their metabolites are absorbed into the systemic circulation after oral administration and could pass through the blood-brain barrier and exert pharmacological activities in the central nervous system. 20(S)-protopanaxadiol, a ginseng saponin metabolite, exerts neuropharmacological effects in the central nervous system by targeting adenylate kinase 5, as identified through multi-proteomic strategies (DARTS/CETSA) and validated via biophysical assays (BLI/ITC) and molecular docking, providing mechanistic insights into ginseng's neuropharmacological actions (Chen et al., 2022, doi: 10.1021/acs.jafc.1c07819). In the other hand, multi-components of TCM may affect multiple target proteins and pathways through synergistic actions, making it difficult to elucidate the mechanism of a single component. Dr. Tian Xie's team demonstrated that the natural isomeric formulation of Curcuma wenyujin (85% β-elemene, 15%

γ/δ-elemene) exhibits superior antitumor efficacy and safety compared to purified β-elemene alone. This finding validates the synergistic advantage in TCM, which multi-component compatibility outperforms single component paradigms (Luo et al., 2024, doi: 10.1016/j.jare.2024.08.005).

3. The clinical research section is weak, is there relevant data from TCM clinical trials to explore specific scenarios for the use of proteomics in efficacy evaluation?

Answer: Thanks for the insightful suggestion. Following the reviewer suggestion, we have revised it in the conclusion section from Page 19 Line 13-29. This manuscript has reviewed and highlighted critical advances in proteomics-driven target discovery for TCM, critically evaluating their respective strengths, disadvantages, and applicability. While these approaches have been widely used *in vitro* target discovery at cell line level, their translation to *in vivo* and clinical applications at systematic level remains constrained by technical challenges. A primary limitation arises from the complexity of *in vivo* systems, where high-abundance proteins (e.g., albumin, immunoglobulins) obscure low-abundance targets, reducing detection sensitivity. Furthermore, small molecule pharmacokinetic introduce unpredictability *in vivo*. In addition, current target proteomics have predominantly relied on acute extracellular or intracellular drug exposure to identify direct targets. In clinical therapies, prolonged small molecules often involve dynamic remodeling of downstream signaling cascades and regulatory networks, obscuring relationships between target binding and clinical outcomes. Certainly, clinical trials remain pivotal for efficacy evaluation, and proteomics has demonstrated promise in identifying diagnostic and prognostic biomarkers. For instance, the analysis of serum proteome changes in psoriasis patients treated with YinXieLing revealed elevated FCN2, MIF, and MMP1 levels as potential biomarkers correlating with therapeutic response (Xu et al., 2019, doi: 10.7150/thno.31144.).

4. The authors are invited to propose research ideas, strategies and outlooks for future research in traditional Chinese medicine based on the existing reviews, combined with practical applications, and in response to the current problems in the research related to the application of proteomics to the identification of targets in TCM.

Answer: Thanks a lot for the perspective suggestion. We have revised it in the conclusion section from Page 19 Line 30 to Page 20 Line 6. Proteomic strategies have provided feasible options for identifying active compounds that can serve as biological probes and new drug candidates. The progress of identifying and targeting active ingredients in TCM will drive more precise and evidence-based practices in its clinical use. The current analysis predominantly focuses on proteomic-based target identification methodologies, with insufficient attention to validation strategies such as Försters resonance energy transfer (FRET), ITC, SPR, SwitchSense, and MST. As proteomics evolves, its integration with complementary technologies such as single-cell proteomics, multi-omics integration (transcriptomics, metabolomics, and proteomics), artificial intelligence-driven target discovery, machine learning-driven prediction, and systems biology will further enhance its capacity to unravel the complexities of TCM.

Highlights

- Innovative proteomic techniques enhance drug-target identification by providing more precision and sensitivity for detecting low-abundance target proteins.
- Recent advances in proteomics strategies for the identification of TCM targets were included.
- Integrating different approaches and platforms proves more conducive in unveiling potential targets of TCM and its active ingredients.

Graphical abstract

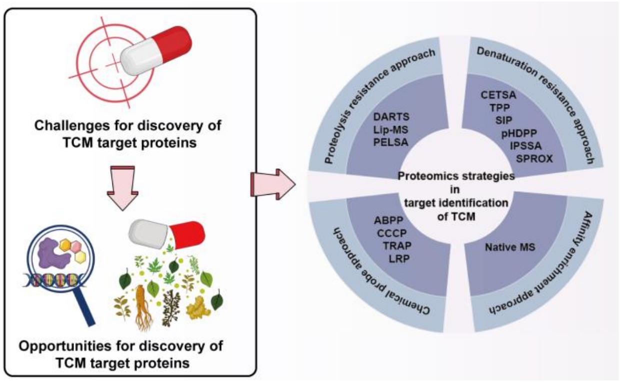

This review highlights proteomic strategies in target identification of Traditional Chinese medicine, elucidating therapeutic mechanisms and evaluting safety and efficacy.

Recent Advances in Proteomic Strategies for Target Identification of Traditional Chinese Medicine

Abstract

Traditional Chinese medicine (TCM) has played an indispensable role in health intervention and disease treatment. Identifying the target proteins of TCM is crucial to clarifying therapeutic mechanisms. One approach taken to enhance the breadth, depth, and precision of studies on the active cellular pathways induced by TCM has been to use proteomics to reveal potential drug targets with direct interactions. Proteomic strategies facilitate identifying and characterizing target binding proteins relevant metabolism pathways, which involves enriching the complex of small molecule and their targets based on the affinity, as well as utilizing the changes in physicochemical properties of target proteins that occur due to drug binding for proteomic identification and quantification. Probe labeling and enrichment technologies have accelerated the field of chemical proteomics. Technologies focused on measuring the changes in target proteins have been widely extended into several different approaches, and these now drive the establishment of further strategies. This review summarizes the advances in proteomics strategies mainly based on mass spectrometry for the identification of TCM targets to the present. Enhancing the application of proteomics would provide a new viewpoint on TCM treatment, while underscores the potential of TCM as biological probes and sources of novel drug candidates.

Keywords: Proteomics Strategies; Target Protein Identification; Traditional Chinese Medicine;

1. Introduction

Traditional Chinese medicine (TCM), with a history spanning thousands of years, generally refers to using a mixture of herbal plants or extracts that contain many bioactive components with varying physicochemical characteristics[1,2]. These bioactive components typically have multiple targets by which they exert their health effects and functions in disease intervention and treatment[3]. Recent clinical studies indicate that TCM components have significant therapeutic and preventative benefits, notably as treatments for COVID-19[4]. Due to the unique pharmacological activity of herbal medicines, TCM presents valuable opportunities for discovery and research, especially those that can target cancer[5,6] and infectious diseases[7]or can prevent cardiovascular and metabolic disorders[8-10], rheumatoid arthritis[11-13], and multiple sclerosis[14,15]. Identifying the target proteins is essential for understanding the molecular mechanisms of TCM; however, this deconvolution process is time-consuming[16,17]. Moreover, multiple bioactive compounds in TCM, along with their complementary functions, present major barriers to investigating the underlying molecular mechanisms.

Fishing out target proteins from the complex cell milieu is challenging, however, the proteomic approach could effectively address this issue[18,19].The complex effects induced by bioactive compounds can be revealed by proteomic identification of their targets and network analysis of the pathways[20,21]. Proteomic technologies focus on screening significant changes in the abundance or modification of proteins, thereby facilitating biomarker discovery and revealing the functional proteins or pathways involved in biological events[22,23]. Due to its high sensitivity and resolution, proteomics strategy is crucial and offers strong potential for characterizing global protein changes, diagnosing and treating major diseases, such as cancer[24,25], autoimmune disease[26,27], Alzheimer's disease[28], infections[29,30] and obesity[31]. Nowadays, advanced proteomics techniques for elucidating drug-target interaction mechanisms have been continually developed and introduced (Figure 1).

Leveraging the specific properties of drugs and proteins is a logical consideration for identifying drug target proteins within the complexes formed between drugs and their binding proteins. These techniques monitor the changes in the stability, proteolysis, and chemical reactions of the target proteins after drug binding and have been developed and widely extended to different approaches, including the cellular thermal shift assay (CETSA), thermal proteome profiling (TPP), protein stability

determinations from rates of oxidation (SPROX), the drug affinity responsive target stability (DARTS), limited proteolysis-mass spectrometry (LiP-MS), peptide-centric local stability assay (PELSA), solvent-induced protein precipitation (SIP), pH-dependent protein precipitation (pHDPP), integrated protein solubility shift assay (IPSSA)[32]. For chemical proteomics, modified drugs or probes are usually needed to specifically enrich the conjugate of its target proteins, which have the advantages of increasing the sensitivity, reproducibility, and flux to overcome the limitations of classical gel separation in identifying low-abundance protein[33-34]. Chemical proteomics technologies consist of two key steps: (1) probe design and synthesis, and (2) target fishing and protein identification, including compound-centric chemical proteomics (CCCP), activity-based protein profiling (ABPP), target responsive accessibility profiling (TRAP) and lysine reactivity profiling (LRP), which are widely used for drug target identification[35,36]. The application of these methods for TCM target identification is summarized as follows.

Recently, a better understanding and more in-depth exploration of the mechanism of TCM has been attained by the application of proteomic strategies in a growing number of studies on biomolecule–target relationships. This review comprehensively summarizes emerging proteomic approaches for TCM target identification, critically evaluating their respective strengths, limitations, and applicability. By systematically analyzing these innovative methodologies, this study aims to construct a framework for addressing persistent technical challenges in the discovery of TCM target proteins, while offering strategic perspectives to advance precision in TCM.

2. Challenges and opportunities for the discovery of TCM target proteins

In recent years, the recognized advantages and health benefits of TCM have gained increasing attention in studies focused on preventing and treating diseases, as a widely used source of core raw materials for natural drugs[37-39]. The bioactive chemical compounds of TCM include volatile oils, alkaloids, flavonoids, glycosides, terpenoids, phenylpropanoids, phenolic acids, phenols, quinones, lactones, and steroid compounds[40-42]; therefore, identifying and clarifying the targets of bioactive TCM components is the basis for elucidating the pharmacological action of TCM. However, the diversity and complexity of TCM components create challenges in studying TCM mechanisms, which often involve multiple processes and targets (Figure 2). To address these challenges, proteomics strategies have become an indispensable tool in the TCM

research arsenal, offering distinct advantages for each approach[43]. Extensive studies have confirmed that the functional mechanisms of TCM extracts or formulas can be characterized using proteomic techniques. Exploring the molecular mechanisms of TCM remains the main theme of ongoing research in this field.

The rapid development of proteomic strategies has significantly advanced the identification of molecular target in complex TCM extracts, eliminating the need for extensive fractionation of the raw material. For example, cryptotanshinone (CTS) and CTS-containing extracts from the roots of *Salvia miltiorrhiza* have been confirmed by DARTS as a promising treatment for keratinopathic ichthyosis by targeting the FKBP prolyl isomerase 1A 160 (FKBP1A) protein[44]. Similarly, an ethanol extract of *Potentilla glabra* var. mandshurica (Maxim.) Hand.-Mazz (Pg-EE) has shown therapeutic potential for autoimmune disorders, arthritis, and diabetes, conditions characterized by excessive and persistent inflammatory responses[45]. Recent application of CESTA confirmed the specific interaction of Pg-EE with its target, the Src protein tyrosine kinase (Src)[46]. Moreover, another study on ethanol extract from *Cissus subtetragona* (Cs-EE) revealed its anti-inflammatory effects, including the inhibition of inflammatory cytokines and the reduction of luciferase activity in Nuclear factor-κB (NF-κB) and Activator protein-1 (AP-1). Further investigation using CETSA technology confirmed Src and TAK1 proteins as key targets of Cs-EE[47].

Recently, Tu's team has developed a traditional Chinese medicine microspheres (TCM-MPs) target fishing strategy that enables the self-assembly and fixation of drug-active small molecules with non-selective properties (Figure 3) [48]. In their work, using Shenqi Jiangtang Granules (a traditional chinese medicine preparations for type 2 diabetes) as an example, this innovative approach provides a new technique for drug target discovery-constructing TCM-MPs to fish for target proteins and combining this with bio-layer interferometry (BLI) to perform reverse screening of active molecules against the target proteins. The highlight lies in fixing different types of active small molecules in TCM to establish an effective target discovery technology, showing promising potential for identifying and investigating targets within complex systems.

3. Proteomic strategies for the identification of TCM targets

Proteomic strategies for target discovery focus on differentiating the whole complex of biomecules and their target proteins from the non-binding proteins in the cell protein reservoir. Therefore, it is mainly based on biomecules-protein binding

interactions that lead to conformational and protein stability changes or that can be used as bait for fishing out a biomecules-protein complex by affinity enrichment. The physicochemical property changes in target proteins after binding enable the division of the techniques into proteolysis resistance approach, denaturation resistance approach and chemical probe approach. The details of these individual methods derived from the proteolysis resistance approach (DARTS, LiP-MS, and PELSA), the denaturation resistance approach (CESTA, TPP, SIP, pHDPP, IPSSA, and SPROX), the chemical probe approach (ABPP, CCCP, TRAP, and LRP), the biomecules-protein complex by affinity enrichment (Native mass) has been reviewed as well.

3.1 Proteolysis resistance approach

3.1.1 Drug affinity responsive target stability

DARTS is a well-established and powerful method for studying drug-binding proteins, based on the principle that drug-target protein complexes are more resistant to proteolysis than free proteins[49]. Drug binding induces the target proteins to assume a specific conformation that masks protease recognition sites, thereby imparting greater resistance to proteolysis. These changes are visualized on sodium dodecyl sulfate-polyacrylamide gel electrophoresis (SDS-PAGE) and identified by liquid chromatography-tandem mass spectrometry (LC-MS/MS)[50] (Figure 4A).

Notably, DARTS is universally applied as it requires no chemical or structural modification of bioactive TCM molecular[51]. The latest advances in TCM target protein discovery supported by DARTS are summarized in Table 1. For instance, it has revealed that shizukaol A exerts its anti-inflammatory effects by targeting high mobility group box 1 (HMGB1), thereby modulating the Nrf2/HO-1 signaling pathway[52]. Similary, based on the DARTS approach, Tumor necrosis factor receptor-associated factor 6 (TRAF6) was identified as a direct target of Trishizukaol A (TSA), a bioactive candidate from *Sarcandra glabra*, which suppressess inflammatory fluxes through the TRAF6/MAPK pathway[53]. In addition, quantitative proteomics and DARTS assays have further shown that daurisoline, isolated from Menispermi Rhizoma, targets heat shock protein 90 (HSP90) in A549 and Hop62 lung cancer cell lines, suggesting its potential therapeutic target for fighting lung cancer[54]. Furthermore, salicin (SA), derived from willow bark (*Salix alba*), alleviates osteoarthritis by directly targeting the endoplasmic reticulum stress regulator inositol-requiring enzyme 1α (IRE1α) in primary articular chondrocytes[55]. Likewise, liquidambaric acid (LDA) has been

identified as a direct target of tumor necrosis factor receptor-associated factor 2 (TRAF2), inhibiting Wnt/β-catenin signaling and presenting new horizons in the treatment of colon cancer[56]. Moreover, dihydromyricetin (DHM), extracted from *Ampelopsis grossedentata*, has been identified as targeting the binding site of glucose regulated protein 78 (GRP78) using DARTS, with direct interactions confirmed by surface plasmon resonance (SPR), elucidating its mechanism in reducing lipid droplet formation in 3T3-L1 cells and highlighting its potential as an anti-obesity agent[57]. Using DARTS and validated by atomic force microscopy imaging, cytosolic phospholipase A2 (cPLA2) has been identified as a direct target of aconitine from the *Aconitum* species, inducing PTGS2/COX-2 expression and inflammatory factor release, ultimately contributing to myocardial injury and dysfunction[58]. Diosmin, screened as an aryl hydrocarbon receptor (AhR) agonist through luciferase assays and confirmed by DARTS, has been shown to directly bind to AhR, upregulating skin barrier proteins (filaggrin and loricrin) and restoring barrier function suppressed by Th2 cytokines, highlighting its potential as a treatment for atopic dermatitis[59]. Additionally, deoxyelephantopin (DET), a sesquiterpene lactone isolated from *Elephantopus scaber* Linn., has been demonstrated significant cytotoxicity against HepG2 and Hep3B hepatocellular carcinoma (HCC) cells. Using DARTS assay, DET was identified as a direct binder of Hsp90α and further studies has revealed that DET induced mitochondrial dysfunction, oxidative stress, and apoptosis, both alone and synergistically with sorafenib, making it a promising candidate for HCC therapy[60]. In addition, natural biomolecular, known for their multi-target characteristics, hold promising therapeutic potential. Glytabastan B, a coumestan isolated from *Glycine tabacina*, has been found to target the ERK2, JNK1 and PI3K catalytic subunit p110 (α and β), effectively inhibiting the MAPK and PI3K/AKT pathways, which suggests GlyB is a promising multiple-target candidate for the prevention of rheumatoid arthritis[61].

Recently, the integration of multiple proteomic strategies has been widely employed in target identification, enhancing reliability, uncovering dynamic interactions, and improving the efficiency of functional analysis. The combination of DARTS and CETSA methods has identified tubocapsenolide A has been shown to target the SHP-2 protein tyrosine phosphatase in U2OS cells[62]. Simiarly, DARTS combined with targeted-limited proteolysis-multiple reaction monitoring identified Poly-ADP-ribose-polymerase-1 (PARP-1) as the target of crellastatin A in HeLa

cells[63]. Ginsenoside metabolite 20(S)-protopanaxadiol (PPD), an active compound in ginseng, has been identified to target adenylate kinase (AK5) in brain tissues through DARTS and CETSA. Subsequent BLI kinetic analysis and isothermal titration calorimetry (ITC) assays confirmed the specific binding and activation of AK5 by PPD, providing valuable insights into pharmacological effects in the central nervous system[64].

DARTS is a common method due to its simple operation and high throughput; however, it has limitations in accurately reflecting *in vivo* drug action based only on proteins extracted *in vitro* to study drug-target interaction[50-51]. Additionally, DARTS is less effective for certain proteins, as some stress-related target proteins are insensitive to proteolytic enzymes under native conditions, and others may be present at very low abundance in cell lysates, limiting their detectability[65].

[illegible].1.2 Limited proteolysis-mass spectrometry

LiP-MS enables the analysis of protein-small molecule interactions within complex samples and can be applied across native bacterial, yeast, and mammalian systems without chemical modification of proteins[66]. LiP-MS technique identifies binding sites by detecting local changes in protease accessibility caused by small molecule binding, producing condition-specific cleavage products. These fragments are further digested with trypsin, and the sequences and abundances are measured by LC-MS/MS to identify the target proteins and provide structural fingerprints[67]. As a structural proteomics approach, LiP-MS is valuable for detecting drug targets, identifying disease-associated protein structures, assessing protein aggregates, and capturing protein structural changes[68] (Figure 4B). Lip-MS effectively identifies small-molecule binding sites and provides structural information without chemical modifications. Similar to DARTS as a proteolysis resistance approach, LiP-MS offers higher resolution, identifying ligand-protein binding site structural details with approximately 12 amino acid precision[69]. However, Lip-MS faces challenges with low-abundance proteins, those without MS-detectable peptides, and proteins undergoing conformational changes[67-68].

LiP-MS is now gradually being applied for the discovery of TCM targets (Table 1). LiP-MS exploration of the mechanism by which isoliquiritigenin (ISO) effectively ameliorates nonalcoholic steatohepatitis (NASH) symptoms in mice identified IQ motif containing GTPase activating protein 2 (IQGAP2) as the direct target protein of ISO in

HepG2 cells[70]. The binding of IQGAP2 and ISO was found to activate the IQGAP2/CREB/SIRT1 pathway to alleviate NASH. Chen et al[71] have confirmed that Hyperforin (HPF) was identified as a potential anti-obesity agent, promoting thermogenesis via an AMPK and PGC-1α-dependent pathway. Using LiP-MS combined with microscale thermophoresis and molecular docking, dihydrolipoamide S-acetyltransferase (Dlat) was confirmed as a direct target of HPF, with Dlat ablation significantly reducing HPF-induced adipose tissue browning, highlighting its potential as a therapeutic lead for obesity treatment. Farrerol, identified through ligand-based proteomics (LiP-MS) and validated by additional biochemical assays, directly targets Ubiquitin C-Terminal Hydrolase L3 (UCHL3), enhancing its activity to promote RAD51 deubiquitination and improve homologous recombination repair. This mechanism restores genomic stability and significantly improves somatic cell nuclear transfer (SCNT) embryo development, providing a novel approach to enhance SCNT efficiency[72]. Scutellarin (SG), a flavonoid glucuronide from *Erigeron breviscapus*, has shown potential therapeutic effects on neurological diseases. Sheng et al., combined Lip-MS with other validation methods, including molecular docking, co-IP, DARTS, and CETSA, revealed that SG selectively inhibits PDK2 activity, a key regulator of mitochondrial glucose oxidation, thereby protecting mitochondria from damage and improving mitochondrial aerobic respiration in cerebral ischemia[73].

3.1.3 Peptide-centric local stability assay

Like DARTS and LiP-MS, PELSA is a limited-proteolysis method that utilizes a large amount of trypsin to profuce peptides directly from native proteins[74]. Unlike DARTS and LiP-MS, which emphasize analyzing partially digested proteins and typically involve a second digestion step, PELSA identifies changes in peptide abundance associated with ligand-binding regions (Figure 4C). Notably, PELSA identified 12-fold more kinase targets than LiP-Quant using multiple drug doses, and 2.4-fold more kinase targets than TPP using multiple temperatures, respectively[74,99]. PELSA emerges as a cutting-edge method with high sensitivity, attributed to its ability to identify targets with lower sequence coverage requirements, generate strong fold changes, and detect kinase targets with extreme melting temperatures, thereby surpassing the limitations of LiP-Quant and TPP[75]. However, it has not yet been applied to the screening of TCM, but holds significant potential for further research.

3.2 Denaturation resistance approach

3.2.1 Cellular thermal shift assay

The thermal stability of a protein can be altered by its interactions with biomolecules, forming the basis of the CETSA method[76] (Figure 4A). The principle underlying CETSA technology is that the binding of a drug molecule changes the thermal stability of the target protein[77]. In this assay, the cells treated with the target compounds of interest are collected, and proteins are extracted under native conditions, and then denatured by heating and precipitated. Monitoring the soluble protein fractions that remain after exposure to a range of temperatures generates melting profiles for each detected protein[78]. Unbound proteins undergo denaturation and precipitation in response to temperature increases, whereas more ligand-bound proteins remain in solution. CETSA is particularly valuable for confirming the target engagement of pharmacologically bioactive molecules[79]. Nevertheless, the usefulness of CETSA is limited to identifying unknown target proteins, and WB-based CETSA is generally used for target verification. The recent emergence of high-throughput CETSA, in combination with reporter luminescence and immunofluorescence detection, has increased the flux of CETSA data[80].

Currently, CETSA technology is extensively utilized in TCM due to its capability to provide quantitative data on target proteins in cell lysates, live cells, and tissues. A summary of applications in investigating therapeutic mechanisms is presented in Table 2. For example, schisandrol A, derived from *Schisandra chinensis*, binds to the ATP6V0D1 subunit of the V-ATPase in PC12 cells, where it plays a role in preventing diabetic neuropathy[81]. Nordihydroguaiaretic acid inhibits Histone Acetyltransferase p300 and activates autophagy, extending lipespan in C. elegans[82]. Wogonoside, shikonin, raddeanin A, and chamaejasmenin E exhibit effective anti-cancer properties[83-86]. Calomembranone G and pentoxifylline target Toll-like receptor 4 (TLR4) to exert anti-inflammatory effects[87,88]. Moreover, eucalyptin C, derived from *Eucalyptus globulus* Labill., bings to Phosphoinositide 3-kinases-γ (PI3Kγ), inhibiting the activation of primary spleen cells from allergic contact dermatitis mice[89].

The combination of DARTS and CETSA has also been widely employed to validate biomolecules-protein interactions. For example, a combined DARTS and WB-CETSA strategy demonstrated costunolide, isolated from *Aucklandia lappa* Decne, targets cyclin-dependent kinase 2 (CDK2) in BV-2 cells[90]; whereas, eupalinolide B,

extracted from *Eupatorium lindleyanum*, targets ubiquitin-specific protease 7 (USP7) in BV-2 cells[91]. Likewise, gentiopicroside, isolated from *Gentiana manshurica* Kitagawa, has been reported to directly bind to progestin and adipoQ receptor 3 (PAQR3), enhanceing PAQR3 degradation via DNA-binding protein 2 (DDB2)-mediated ubiquitination, as confirmed by CETSA, SPR, and microscale thermophoresis (MST)[92]. Ligustilide, a key active component of Suxiao Jiuxin pills widely used in cardiovascular disease treatment, covalently binds to Calcium-calmodulin-dependent protein kinase II (CaMKII) at Cys116, inducing a long-lasting vasodilator effect. Through a combination of CETSA, iTRAQ-based proteomics, in-gel imaging, and molecular docking, it has been confirmed that the epoxidized metabolite of ligustilide effectively targets CaMKII in vascular smooth muscle cells, providing new insights into its potential for cardiovascular disease treatment[93]. In addition, proanthocyanidin A1 has been shown to directly bind to Janus kinase 2 (JAK2), as confirmed by isothermal dose-response fingerprint-CETSA, molecular docking, kinase activity, and SPR. This binding activates the JAK2/STAT3 pathway and ameliorates chemotherapy-induced thrombocytopenia[94]. Furthermore, hyodeoxycholic acid (HDAC), an active ingredient of traditional Chinese medicine pig bile, has been emphasized its ability to ameliorate NAFLD. In this study, a human protein microarray recognized ras-related nuclear protein (RAN) as a direct target of HDAC, which was further validated through various techniques, including immunoprecipitation (IP), proximity ligation assay (PLA), CETSA, and molecular docking. Bind of HDCA to RAN disrupts the RAN/CRM1/PPARα shuttling complex, leading to increased nuclear accumulation of PPARα and enhanced fatty acid oxidation, providing a therapeutic mechanism for ameliorating NAFLD[95]. GA-amide, an analog of gambogic acid that constitutes a primary active constituent of the traditional Chinese medicine, gamboge, has been found to directly bind WD repeat domain 1 (WDR1), by combining CETSA, DARTS, molecular docking simulation, and SPR, which inhibited glioma cell invasion and induced apoptosis[96].

CESTA enables direct biophysical studies in intact cells and validates target proteins identified by other methods but is unsuitable for unknown target discovery, affects cell membrane permeability, and has challenges with reproducibility and highly inhomogeneous proteins[77-80].

3.2.2 Thermal proteome profiling

TPP, an optimized version of CETSA, is a target-deconvolution technique used to discover small molecule targets in TCM (Figure 5A). The core principle of TPP is that proteins become more resistant to heat-induced unfolding when combined with a ligand[97]. Proteins alter their thermal stability because of interacting with small molecule drugs, nucleic acids, or other proteins, or by undergoing post-translational modifications. The reduced susceptibility to protein precipitation conferred by ligand protection during the gradient heating process can be accurately quantified by MS analysis[98]. Both CETSA and TPP rely on the thermal stability of ligand-bound proteins; however, the MS-based TPP technology enables the high-throughput analysis of numerous proteins in profiling mode and has high efficiency for screening drug-binding target proteins[99]. TPP requires no compound modification and can provide high-throughput identification of intracellular targets in living cells[100]. Distinctively, TPP is distinguished by its potential for *in vivo*, in situ, or *in vitro* applications, and has been successfully applied to identify targets and off-targets of drugs or to study protein-metabolite and protein-protein interactions. Therefore, TPP provides unique insights into protein states and interactions in their native context and at a proteome-wide level, thereby facilitating the study of basic biological processes and their underlying mechanisms.

Recently, TPP technology has been increasingly applied in TCM research, as depicted in Table 2. TPP has confirmed that artone, isolated from the herb *Artemisia giraldii*, could directly target Histone chaperone ASF1A (ASF1α) in BV-2 cells, significantly inhibiting neuroinflammation and inflammation-associated neurodegenerative diseases[101]. Kurarinone, derived from Sophorae Flavescentis Radix, has been demonstrated to target and inhibit the soluble epoxide hydrolase in MPTP-induced Parkinson's disease mice[102]. Conophylline (CNP), a vinca alkaloid extracted from the *Tabernaemontana divaricata*, has been identified as a binding partner for glutathione peroxidase 4 (GPX4) using TPP, leading to lipid reactive oxygen species accumulation and autophagy[103]. In addtion, Yang et al., employed an unbiased TPP method to identify multiple targets of flavonoid 4,4′-dimethoxychalcone, further valiadating Aldehyde Dehydrogenase 1 Family Member A3 (ALDH1A3) as a target for inhibiting A549 cells[104].

3.2.3 Solvent-induced protein precipitation

The SIP approach is an energetics-based proteomics technique developed to

investigate biomolecules-protein interactions in cell lysates by exploiting the increased resistance of biomolecules-binding protein to organic solvent-induced denaturation and precipitation (e.g., acetone, ethanol, or acetic acid) (Figure 5B and Table 2). Further optimization is needed to do determine the appropriate organic solvent concentration[105]. For example, SIP screening has demonstrated that sinomenine directly targets guanylate-binding protein 5 (GBP5) to treat rheumatoid arthritis in lipopolysaccharide-stimulated RAW264.7 cells, a result further comfirmed by CETSA validation[106]. Furthermore, combining SIP with TPP has revealed that shikonin targets the NF-κB essential modulator NEMO/IKKβ complex, effectively inhibting the growth of colorectal cancer cells[107]. SIP allows drug target identification without drug modification but lacks multiplexing for comprehensive proteome-wide analysis[152].

3.2.4 pH-dependent protein precipitation

pHDPP method assesses protein stability changes induced by ligand binding by treating proteins with an acidifying agent, causing them to gradually denature and precipitate, with ligand-binding proteins showing a shallower precipitation gradient compared to non-binding proteins, as measured by mass spectrometry (Figure 5C)[108]. A critical factor is the controlling the concentration of the acidifying agent. The applications of this technique are shown in Table 2; for instance, pHDPP has identified dihydroartemisinin binding to aldehyde dehydrogenase ALDH7A1 and HMGB1 in Hela cells, and further validated by CETSA[108]. The combination of SIP and pHDPP technologies enhances high sensitivity in target protein screening. However, the cell membrane hinders the penetration of organic solvents, making this approach unsuitable for intact-cells experiments, as targets on the cell surface or structural changes after lysis are ignored. pHDPP works for multiple ligands with high sensitivity and complements other proteomic methods, though acidic agents may disrupt acid-base equilibrium in small molecule drugs.

3.2.5 Integrated protein solubility shift assay

Nowadays, the integrated protein solubility shift assay (IPSSA) has been developed to optimize workflows by integrating multiple assays, including pH shift assay, thermal shift assay and solvent shift assay (Figure 5D). The IPSSA approach analyzes ligand-induced protein solubility changes, which improves sensitivity in target

identification (up to 38%) compared to individual methods[109]. Using staurosporine, a pan-kinase inhibitor, the study validated the IPSSA approach, highlighting its ability to detect drug targets with increased statistical power and fewer false positives[109]. Although, IPSSA technology has not yet been applied in TCM, it demonstrates strong potential in highly sensitive target exploration capabilities.

3.2.6 Stability of proteins from rates of oxidation

SPROX assesses the thermodynamic stability of proteins and protein-ligand complexes at the peptide level, utilizing covalent labeling through quantitative, bottom-up, shotgun proteomics[110]. SPROX assays utilize the chemical denaturant dependence of a hydrogen peroxide-mediated oxidation reaction with methionine side chains to generate information about the global thermodynamic stability of proteins[111,112] (Figure 5E). However, a key limitation of this approach is that it relies on detection and quantification of methionine oxidation rate in peptide segments, which are relatively rare in protein sequences. Moreover, SPROX requires higher concentrations of drugs (μmol/L to mmol/L)[113,114], which poses challenges in obtaining sufficient amounts of TCM components. Therefore, the application of SPROX in investigating the active mechanisms of TCM has been limited. SPROX could detect temperature and enzyme-insensitive proteins but requires higher compound concentrations and only identifies proteins susceptible to selective methionine oxidation.

3.3 Chemical probe approach

3.3.1 Activity-based protein profiling

ABPP is a chemical proteomics approach widely used to identify the protein targets of small molecules and even the active sites of target proteins[115,116]. The core principle involves activity-based probes (ABPs) to report the activity of specific enzymes or the reactivity of amino acid types in complex biological systems[117]. A critical aspect is the design and synthesis of the ABPs, as they must retain the pharmacological activity of the original molecule and allow for efficient enrichment and identification of binding protein targets. ABPP has matured into a standard technology for the rapid, sensitive, and selective profiling of enzyme activity and inhibitors in proteomes, especially useful when compound-specific probes are unavailable or display weak target affinity[118,119] (Figure 6A). ABPP technology is

particularly suitable for TCM with low abundance of the active ingredient. However, limitations of ABPP include the finite number of molecules that can be chemically modified without altering interactions with the target protein, potentially resulting in false positives.

Extensive evidence summarized in Table 3. For instance, cucurbitacin B has been shown to bind to GRP78, inhibiting the GRP78-FOXM1-KIF20A pathway, thereby supressing the proliferation of conjunctival melanoma cells[120]. Moreover, ABPP has demonstrated that daphnane diterpenoid, isolated from the *Daphne genkwa*, directly targets importin-β1 in prostate cancer cells. This interaction was subsequently confirmed by WB-CETSA, supporting the potential of daphnane diterpenoid as a promising therapeutic agent for castration-resistant prostate cancer[121]. In addition, a combination of ABPP and WB-CETSA technology revealed that celastrol directly targets peroxiredoxins (PRDX) to ameliorate hepatic fibrosis in LX-2 cells[122]. With affinity-based protein profiling, withangulatin A (WA) has been identified as a novel covalent inhibitor of phosphoglycerate dehydrogenase (PHGDH), directly binding to Cys295 and inactivating its enzyme activity, which was further validated by BIL and LC-MS/MS, demonstrating selective binding to PHGDH, blocking its substrate-binding domain, and inhibiting tumor proliferation[123]. Similarly, through SILAC-ABPP, WA was identified as a novel covalent inhibitor of Peroxiredoxin 6 (PRDX6) . Further validation using CETSA, DARTS, and BLI assays confirmed that WA binds specifically to the cysteine 47 residue, inhibiting both its glutathione peroxidase and phospholipase A2 activities, suggesting WA as a potential anti-tumor agent[124]. Besides, ABPP revealed that Parthenolide (PN) preferentially modified specific targets, with proteomics analysis further highlighting its role in the ubiquitin-mediated proteolysis pathway. Subsequent validation confirmed that PN covalently modified Ubiquitin carboxyl-terminal hydrolase 10 (USP10) at Cys40, inhibiting breast cancer cell proliferation through LC-MS/MS, USP10 knockdown, and deubiquitinating enzyme activity assays, consistent with our ABPP findings[125]. Furthermore, diacylglycerol kinase family member DGKQ was identified as a direct target of the phytochemical atractylenolide II (AT II) through ABPP mapping, with further validation using CETSA, DARTS, SPR, and microscale thermophoresis. AT II acts on a novel drug-binding pocket in the CRD and PH domains of DGKQ, allosterically regulating its kinase activity, which in turn improves obesity-induced insulin resistance and hyperlipidemia[126]. Chromatin assembly factor 1 subunit (CHAF1B) has been

identified as key target of puerarin in protecting cardiomyocytes from apoptosis under high glucose through ABPP, CETSA, and knockdown[127]. capsaicin (CAP) has been identified as a direct binder of pyruvate kinase (PKM2) and lactate dehydrogenase A (LDHA) through streamlined cysteine-ABPP, CETSA-WB, SPR, and pull-down assays, offering promising therapeutic strategies for sepsis and inflammation[128]. ABPP identified Proliferating cell nuclear antigen (PCNA) as a potential target of BVC, with the interation confirmed by CETSA, DARTS, and SPR, which elucidates that Bavachinin exert hepatoprotective effects in non-alcoholic fatty liver disease (NAFLD)[129]. Additionally, photo-affinity labeling-ABPP has demonstrated that 3-O-trans-p-coumaroyl maslinic acid (OCMA) isolated from *Ligustrum lucidum* Ait. binds to the S1 subsite of γ-secretase[130]. The advancements of ABPP underscore versatility in identifying targets and mechanisms in complex biological systems. ABPP enables rapid, sensitive, and selective detection of enzyme activity and inhibitors but struggles with low-abundance proteins, requires probe synthesis that can alter drug activity, and necessitates chemical modification of compounds.

3.3.2 Compound-centered chemical proteomics

The CCCP technique is a simple and chromatography based approach that involves covalent immobilization of small molecule (whose bioactivity is typically known) onto a solid matrix, such as agarose, followed by incubation with a protein lysate to capture the interacting proteins[131,132] (Figure 6A). Compared to ABPP, CCCP disabled to identify the activation state of target proteins but offers a more unbiased approach, even enabling the identification of no enzymatic targets, thereby facilitating the discovery novel targets[133]. In recent years, CCCP technology has successfully identified small molecule targets in TCM, as summarized in Table 3. It has revealed that artemisinin, an active compound, targets the gephyrin protein in MIN6 cells, which is responsible for the regeneration of pancreatic β cell mass from α cells in the treatment of Type 1 diabetes[134]. Curcumol, isolated from Rhizoma Curcumae, directly targets the nucleolin protein innasopharyngeal carcinoma cells[135]. CCCP enhances selectivity and identifies non-enzymatic targets, though nonspecific binding increases false positives and immobilizing bioactive compounds is challenging.

3.3.3 Target responsive accessibility profiling

TRAP is an emerging chemoproteomics target discovery approach that measures protein changes induced by ligand binding to global lysines[136] (Figure 6B). Unlike classical probe-based chemoproteomics, TRAP avoids the need for derivatization, allowing high-coverage and high-throughput identification of the targets of multiple drugs, making it particalularly promising for TCM target discovery. Recent applications of TRAP in TCM target discovery are detailed in Table 3. For instance, the combination of TRAP and CETSA has been used to show that the antitumor immunity and mechanism of cycloastragenol involve a reduction in the degradation of MHC-I in MC38 cells via directly targeting cathepsin B (CTSB)[137]. Besides, TRAP has demonstrated that celastrol binds adenylyl cyclase associated protein 1 (CAP1) in THP-1 cells to inhibit resistin-induced inflammation, as confirmed by CETSA[138]. TRAP can also applied in living cells; for example, in combination with thermal shift assays and DARTS, TRAP has validated that silibinin targets ACSL4 in living HepG2 lysates to protect from ferroptosis[139]. Lobeline, an alkaloid from the herbal medicine lobelia, could remodel the immunosuppressive microenvironment. Using TRAP,16 potential target proteins were identified, and further confirmed the binding lobeline and MAPK14 through ITC, molecular docking, and site-directed mutagenesis[140]. TRAP eliminates the need for synthesizing photo-affinity probes and allows target deconvolution of metabolites, but it requires protein lysine labeling and may produce false positives.

3.3.4 Lysine reactivity profiling

The LRP strategy, based on active dimethyl labeling, has been developed to for mass spectrophotometric detection of the patterns of conformation modulation due to molecule ligand binding to protein targets[141-144] (Figure 6B). This approach facilitates the discovery of biomolecule targets by tracking conformational changes, such as hydrogen bond formation or electrostatic interactions at lysines in protein-protein interaction interface[145]. However, the detection sensitivity of conformational changes at the proteome level is limited to the sensitivity. To date, few studies on the identification of TCM targets using LRP strategies.

3.4 Native mass spectrometry

Native MS offers the distinct advantage of allowing direct investigation of protein-ligand interactions under non-denaturing conditions, which could detect both non-

covalent and covalent complexes, enabling the identification of ligands through mass-to-charge ratio shifts and precise molecular weight calculations from the mass differences between unbound and bound proteins[146] (Figure 7).

Littler et al. have developed native MS based on high-resolution ESI-FTICR-MS to screen the binding ligands to SARS-CoV-2 Nsp9 protein in the 1614 natural product library, which oridonin has the strongest binding affinity to Nsp9[147]. Quinn and colleagues have developed a fragment-based approach using native MS to investigate potential protein targets for antimalarial. They have identified 96 low-molecular-weight natural products as binding partners of 32 putative malarial targets, with 79 of these compounds showing in vitro antimalarial activity[148]. Moreover, an integrated untargeted metabolomics-native MS workflow[149] has been applied to high-throughput screening against human carbonic anhydrase I (hCAI) from crude nature product extracts. Following low-volume gel filtration, native MS coupled with nanoscale ion emitters enabled direct screening of intact protein-biomolecule complexes from complex natural product extracts. Subsequently, biomolecules were further dissociated and identified via multistage mass spectrometry (MS^n). Screening against three targets (bovine carbonic anhydrase II, human CAVII, and lysozyme) revealed 14 distinct biomolecules. Native MS has become a powerful tool for identifying native binding sites without the need for protein digestion and labeling to screen the crude extracts.

4. Summary and prospects

Proteomics techniques have emerged as a cornerstone in early-stage drug discovery (Figure 8), offering powerful tools for target identification, validation, and evaluation of safety and efficacy. In the field of TCM, proteomics techniques are revolutionizing the discovery of molecular targets, shedding light on the complex interactions between bioactive compounds and their biological pathways. This review delves into recent advances in proteomics application for TCM target discovery and highlights the directions to harness their full potential.

Recent strides in proteomics have enabled significant progress in characterizing and identifying the molecular targets of TCM, which have illuminated the multifaceted and mechanisms underlying therapeutic effects and pharmacological basis of TCM. However, despite these achievements, current proteomics strategies still face challenges (Table 4). Each technique has distinct strengths and limitations regarding sensitivity,

resolution and scalability. More research technologies and better application of currently available proteomic strategies are needed to tap the full drug potential of TCM. Among the emerging technologies, hydrogen deuterium exchange mass spectrometry (HDX-MS) stands out for its high sensitivity in probing the conformational dynamics of protein molecule interactions[150]. As HDX-MS gains traction, its application in TCM research is poised to bridge critical gaps in target validation and mechanism elucidation. Currently, no single technique provides a comprehensive, “one-stop shop” solution for target identification[151]. The different proteomic strategies available for identifying TCM targets are strongly complementary. Hence, integrating different proteomics approaches and platforms represents a critical opportunity to overcome individual limitations and achieve a more holistic understanding of molecular interactions.

The complexity of TCM components is primarily reflected in the synergistic effects of multiple components and the complexity of metabolites. Orally administered small molecules from TCM often undergo phase I/II metabolism, generating bioactive derivatives that may diverge mechanistically from their parent compounds, which may affect the identification of direct targets in proteomics. For instance, Ginsenosides and their metabolites are absorbed into the systemic circulation after oral administration and could pass through the blood-brain barrier and exert pharmacological activities in the central nervous system. 20(S)-protopanaxadiol, a ginseng saponin metabolite, exerts neuropharmacological effects in the central nervous system by targeting adenylate kinase 5, as identified through multi-proteomic strategies (DARTS/CETSA) and validated via biophysical assays (BLI/ITC) and molecular docking, providing mechanistic insights into ginseng's neuropharmacological actions[153]. In the other hand, multi-components of TCM may affect multiple target proteins and pathways through synergistic actions, making it difficult to elucidate the mechanism. Xie's team has demonstrated that the natural isomeric formulation of Curcuma wenyujin (85% β-elemene, 15% γ/δ-elemene) exhibits superior antitumor efficacy and safety compared to purified β-elemene alone. This finding validates the synergistic advantage in TCM, which multi-component compatibility outperforms single component paradigms[154]. To address the challenges, advanced proteomic strategies to deconvolute multi-component mixtures have enabled the discovery of promising therapeutic leads with diverse pharmacological activities. Native MS integrated with online fractionation enables high-throughput characterization of endogenous protein complexes, directly

capturing ligand, particularly when combined with multistage MS, holds significant potential for mapping dynamic protein interactions, identifying unknown ligands, and elucidating functional relationships within metabolic pathways on a global scale[155].

This paper has reviewed and highlighted critical advances in proteomics-driven target discovery for TCM, critically evaluating their respective strengths, disadvantages, and applicability. While these approaches have been widely used in vitro target discovery, their translation to *in vivo* and clinical applications remains constrained by technical challenges. A primary limitation arises from the complexity of in vivo systems, where high-abundance proteins (e.g., albumin, immunoglobulins) obscure low-abundance targets, reducing detection sensitivity. Furthermore, small molecule pharmacokinetics introduce unpredictability *in vivo*. In addition, current target proteomics have predominantly relied on acute extracellular or intracellular drug exposure to identify direct targets. In clinical therapies, prolonged small molecules often involve dynamic remodeling of downstream signaling cascades and regulatory networks, obscuring relationships between target binding and clinical outcomes. Certainly, clinical trials remain pivotal for efficacy evaluation, and proteomics has demonstrated promise in identifying diagnostic and prognostic biomarkers. For instance, the analysis of serum proteome changes in psoriasis patients treated with YinXieLing revealed elevated FCN2, MIF, and MMP1 levels as potential biomarkers correlating with therapeutic response[156].

Proteomic strategies have provided feasible options for identifying active compounds that can serve as biological probes and new drug candidates. Looking ahead, the value of identifying and targeting active ingredients in TCM will drive more precise and evidence-based practices in its clinical use. The current analysis predominantly focuses on proteomic-based target identification methodologies, with insufficient attention to validation strategies such as Försters resonance energy transfer (FRET), ITC, SPR, SwitchSense, and MST. As proteomics evolves, its integration with complementary technologies such as single-cell proteomics, multi-omics integration (transcriptomics, metabolomics, and proteomics), artificial intelligence-driven target discovery, machine learning-driven prediction, and systems biology will further enhance its capacity to unravel the complexities of TCM.

In conclusion, while current proteomic approaches have transformed TCM target discovery, their potential remains largely untapped. Continued refinement of existing methods and the development of innovative technologies will be instrumental in

addressing the challenges posed by TCM's complexity. By advancing proteomic strategies and fostering interdisciplinary collaborations, researchers can fully harness the therapeutic potential of TCM, ultimately enriching global healthcare with its time-honored wisdom and evidence-based efficacy.

References

[1] K. Chan, Progress in traditional Chinese medicine, Trends Pharmacol. Sci. 16 (1995) 182-187.

[2] F.S. Li, J.K. Weng, Demystifying traditional herbal medicine with modern approaches, Nat. Plants, 3 (2017) 17109.

[3] G. Nestler, Traditional Chinese medicine, Med. Clin. North. Am. 86 (2002) 63-73.

[4] K. Huang, P. Zhang, Z. Zhang, J.Y. Youn, C. Wang, H. Zhang, et al., Traditional Chinese medicine (TCM) in the treatment of COVID-19 and other viral infections: efficacies and mechanisms, Pharmacol. Ther. 225 (2021) 107843.

[5] X.Y. Zhang, H. Qiu, C.S. Li, P.P. Cai, F.H. Qi, The positive role of traditional Chinese medicine as an adjunctive therapy for cancer, Biosci. Trends. 15 (2021) 283-298.

[6] L. Dong, D. Lu, R. Chen, Y. Lin, H. Zhu, Z. Zhang, et al., Proteogenomic characterization identifies clinically relevant subgroups of intrahepatic cholangiocarcinoma, Cancer Cell. 40 (2022) 70-87.

[7] L. Luo, J. Yang, C. Wang, J. Wu, Y. Li, X. Zhang, et al., Natural products for infectious microbes and diseases: an overview of sources, compounds, and chemical diversities, Sci. China Life Sci. 65 (2022) 1123-1145.

[8] D. Bonnefont-Rousselot, Resveratrol and cardiovascular diseases, Nutrients. 8 (2016) 250.

[9] L. Gong, S. Guo, Z. Zou, Resveratrol ameliorates metabolic disorders and insulin resistance in high-fat diet-fed mice, Life Sci. 242 (2020) 117212.

[10] T. Meng, X. Li, C. Li, J. Liu, H. Chang, N. Jiang, et al., Natural products of traditional Chinese medicine treat atherosclerosis by regulating inflammatory and oxidative stress pathways, Front Pharmacol. 13 (2022) 997598.

[11] S. Dudics, D. Langan, R.R. Meka, S.H. Venkatesha, B.M. Berman, C.T. Che, et al., Natural products for the treatment of autoimmune arthritis: their mechanisms of action, targeted delivery, and interplay with the host microbiome, Int. J. Mol. Sci. 19 (2018) 2508.

[12] L. An, Z. Li, L. Shi, L. Wang, Y. Wang, L. Jin, et al., Inflammation-targeted celastrol nanodrug attenuates collagen-induced arthritis through NF-κB and Notch1 pathways, Nano Lett. 20 (2020) 7728-7736.
[13] M. Jing, J. Yang, L. Zhang, J. Liu, S. Xu, M. Wang, et al., Celastrol inhibits rheumatoid arthritis through the ROS-NF-κB-NLRP3 inflammasome axis, Int. Immunopharmacol. 98 (2021) 107879.
[14] S. Yu, M. Liu, K. Hu, Natural products: potential therapeutic agents in multiple sclerosis, Int Immunopharmacol. 67 (2019) 87-97.
[15] M. Dou, X. Zhou, L. Li, M. Zhang, W. Wang, M. Wang, et al., Illumination of molecular pathways in multiple sclerosis lesions and the immune mechanism of matrine treatment in EAE, a mouse model of MS, Front Immunol. 12 (2021) 640778.
[16] T.A. Tolvanen, Current Advances in CETSA, Front Mol. Biosci. 9 (2022) 866764.
[17] D.V. Titov, J.O. Liu, Identification and validation of protein targets of bioactive small molecules, Bioorg. Med. Chem. 20 (2012) 1902-1909.
[18] A.G. Atanasov, S.B. Zotchev, V.M. Dirsch, T. International Natural Product Sciences, C.T. Supuran, Natural products in drug discovery: advances and opportunities, Nat. Rev. Drug Discov. 20 (2021) 200-216.
[19] A. Rasul, A. Riaz, I. Sarfraz, S.G. Khan, G. Hussain, R. Zara, et al., Target identification approaches in drug discovery. Scotti, M.T., Bellera, C.L, Drug Target Selection and Validation. Computer-Aided Drug Discovery and Design, Vol. 1, Springer, Cham, 2022, pp. 41-59.
[20] M.M. Liu, W.C. Van Voorhis, R.J. Quinn, Development of a target identification approach using native mass spectrometry, Sci. Rep. 11 (2021) 2387.
[21] H.W. Zhang, C. Lv, L.J. Zhang, X. Guo, Y.W. Shen, D.G. Nagle, et al., Application of omics- and multi-omics-based techniques for natural product target discovery, Biomed. Pharmacother. 141 (2021) 111833.
[22] M. Cui, C. Cheng, L.J. Zhang, High-throughput proteomics: a methodological mini-review, Lab Invest. 102 (2022) 1170-1181.
[23] Y.W. Kwon, H.-S. Jo, S. Bae, Y. Seo, P. Song, M., et al., Application of proteomics in cancer: recent trends and approaches for biomarkers discovery, Fron. Med. 8 (2021) 747333.
[24] H.T. Tan, Y.H. Lee, M.C. Chung, Cancer proteomics, Mass Spectrom. Rev. 31 (2012) 583-605.

[25] C.H.Y. Cheung, H.F. Juan, Quantitative proteomics in lung cancer, J. Biomed. Sci. 24 (2017) 37.
[26] A. Häggmark, C. Hamsten, E. Wiklundh, C. Lindskog, C. Mattsson, E. Andersson, et al., Proteomic profiling reveals autoimmune targets in sarcoidosis, Am. J. Respir. Crit. Care Med. 191 (2015) 574-583.
[27] R.M. Berbers, J. Drylewicz, P.M. Ellerbroek, J.M. van Montfrans, V. Dalm, P.M. van Hagen, et al., Targeted proteomics reveals inflammatory pathways that classify immune dysregulation in common variable immunodeficiency, J. Clin. Immunol. 41 (2021) 362-373.
[28] B. Bai, D. Vanderwall, Y. Li, X. Wang, S. Poudel, H. Wang, et al., Proteomic landscape of alzheimer's disease: novel insights into pathogenesis and biomarker discovery, Mol. Neurodegener. 16 (2021) 55.
[29] L.J. Zhang, X.F. Jia, J.O. Jin, H.Z. Lu, Z.M. Tan, Recent 5-year findings and technological advances in the proteomic study of HIV-associated disorders, Genom. Proteom. Bioinf. 15 (2017) 110-120.
[30] T.M. Greco, I.M. Cristea, Proteomics tracing the footsteps of infectious disease, Mol. Cell Proteomics. 16 (2017) S5-S14.
[31] F. He, H. Jiang, C. Peng, T. Wang, R. Xiao, M. Chen, et al., Hepatic glucuronyl C5-epimerase combats obesity by stabilizing GDF15, J. Hepatol. 79 (2023) 605-617.
[32] G. Li, X. Peng, Y. Guo, S. Gong, S. Cao, F. Qiu, Currently available strategies for target identification of bioactive natural products, Front Chem. 9 (2021) 761609.
[33] T. Böttcher, M. Pitscheider, S.A. Sieber, Natural products and their biological targets: proteomic and metabolomic labeling strategies, Angew. Chem. Int. Ed. Engl. 49 (2010) 2680-2698.
[34] M.H. Wright, S.A. Sieber, Chemical proteomics approaches for identifying the cellular targets of natural products, Nat. Prod. Rep. 33 (2016) 681-708.
[35] X. Chen, Y. Wang, N. Ma, J. Tian, Y. Shao, B. Zhu, et al., Target identification of natural medicine with chemical proteomics approach: probe synthesis, target fishing and protein identification, Signal Transduct. Target Ther. 5 (2020) 72.
[36] F. Huang, B. Zhang, S. Zhou, X. Zhao, C. Bian, Y. Wei, Chemical proteomics: terra incognita for novel drug target profiling, Chin J. Cancer. 31 (2012) 507-518.
[37] P.P. Hao, F. Jiang, J. Cheng, L.Y. Ma, Y. Zhang, Y.X. Zhao, Traditional Chinese medicine for cardiovascular disease evidence and potential mechanisms, J. Am.

Coll. Cardiol. 69 (2017) 2952-2966.
[38] X.H. Shen, F.G. Yin, The mechanisms and clinical application of traditional Chinese medicine Lianhua-Qingwen capsule, Biomed. Pharmacother. 142 (2021) 111998.
[39] C.Q. Li, H.M. Lei, Q.Y. Hu, G.H. Li, P.J. Zhao, Recent advances in the synthetic biology of natural drugs, Front Bioeng. Biotechnol. 9 (2021) 691152.
[40] X. Luan, L.J. Zhang, X.Q. Li, K. Rahman, H. Zhang, H.Z. Chen, et al., Compound-based Chinese medicine formula: From discovery to compatibility mechanism, J. Ethnopharmacol. 254 (2020) 112687.
[41] L. Bu, O. Dai, F. Zhou, F. Liu, J.F. Chen, C. Peng, et al., Traditional Chinese medicine formulas, extracts, and compounds promote angiogenesis, Biomed. Pharmacother. 132 (2020) 110855.
[42] o. Neda, P. Vlazan, R. Oana, P. Sfarloaga, I. Grozescu, A.E. Segneanu, Peptide and amino acids separation and identification from natural products, Anal Chem. (2012).
[43] Y. Lao, X. Wang, N. Xu, H. Zhang, H. Xu, Application of proteomics to determine the mechanism of action of traditional Chinese medicine remedies, J. Ethnopharmacol. 155 (2014) 1-8.
[44] S. Esch, S. Konig, B. Bopp, J. Jose, S. Brandt, A. Hensel, Cryptotanshinone from Salvia miltiorrhiza roots reduces cytokeratin CK1/10 expression in keratinocytes by activation of peptidyl-prolyl-cis-trans-isomerase FKBP1A, Planta Med. 85 (2019) 552-562.
[45] MS. Lee, Role of innate immunity in diabetes and metabolism: recent progress in the study of inflammasomes, Immune. Netw. 11 (2011) 95-99.
[46] H. Kim, K.K. Shin, H.G. Kim, M. Jo, J.K. Kim, J.S. Lee, et al., Src/NF-κB-targeted anti-inflammatory effects of Potentilla glabra var. Mandshurica (Maxim.) Hand.-Mazz. Ethanol Extract, Biomolecules. 10 (2020) 648.
[47] L. Rahmawati, N. Aziz, J. Oh, Y.H. Hong, B.Y. Woo, Y.D. Hong, et al., Cissus subtetragona Planch. ameliorates inflammatory responses in LPS-induced macrophages, HCl/EtOH-induced gastritis, and LPS-induced lung injury via attenuation of Src and TAK1, Molecules. 26 (2021) 6073.
[48] H. Zhang, J.Y. Yao, G.Y. Xiao, J.H. Xie, S.Y. Mao, C.H. Sun, J.C. Yao, J.Z. Yan, P.F Tu, Discovery of drug targets based on traditional Chinese medicine microspheres (TCM-MPs) fishing strategy combined with bio-layer

interferometry (BLI) technology, Anal Chim. Acta. 29 (2024) 342542.

[49] B. Lomenick, G. Jung, J.A. Wohlschlegel, J. Huang, Target identification using drug affinity responsive target stability (DARTS), Curr. Protoc. Chem. Biol. 3 (2011) 163-180.

[50] Y.S. Ren, H.L. Li, X.H. Piao, Z.Y. Yang, S.M. Wang, Y.W. Ge, Drug affinity responsive target stability (DARTS) accelerated small molecules target discovery: principles and application, Biochem. Pharmacol. 194 (2021) 114798.

[51] B. Lomenick, R. Hao, N. Jonai, R.M. Chin, M. Aghajan, S. Warburton, et al., Target identification using drug affinity responsive target stability (DARTS), Proc. Natl. Acad. Sci U S A. 106 (2009) 21984-21989.

[52] P. Tang, Q. Li, S. Liao, S. Wei, L. Cui, W. Xu, et al., Shizukaol A exerts anti-inflammatory effect by regulating HMGB1/Nrf2/HO-1 pathway, Phytomedicine. 82 (2021) 153472.

[53] R. Tao, P. Tang, J. Gao, J. Li, Y. Sun, J. Luo, Y. Li, The anti-inflammatory activity by suppressing the TRAF6/MAPKs pathway of trishizukaol a from Sarcandra glabra, Phytomedicine. 98 (2022) 153952.

[54] X.H. Huang, X. Yan, Q.H. Zhang, P. Hong, W.X. Zhang, Y.P. Liu, et al., Direct targeting of HSP90 with daurisoline destabilizes β-catenin to suppress lung cancer tumorigenesis, Cancer Lett. 489 (2020) 66-78.

[55] Z. Zhu, S. Gao, C. Chen, W. Xu, P. Xiao, Z. Chen, et al., The natural product salicin alleviates osteoarthritis progression by binding to IRE1α and inhibiting endoplasmic reticulum stress through the IRE1α-IκBα-p65 signaling pathway, Exp. Mol. Med. 54 (2022) 1927-1939.

[56] R. Yan, H. Zhu, P. Huang, M. Yang, M. Shen, Y. Pan, et al., Liquidambaric acid inhibits Wnt/beta-catenin signaling and colon cancer via targeting TNF receptor-associated factor 2, Cell Rep. 38 (2022) 110319.

[57] B. Sun, D. Tan, D. Pan, M.R. Baker, Z. Liang, Z. Wang, et al., Dihydromyricetin imbues antiadipogenic effects on 3T3-L1 cells via direct interactions with 78-kDa glucose-regulated protein, J. Nutr.151 (2021) 1717-1725.

[58] J. Wei, S. Fan, H. Yu, L., Shu, Y. Li, A new strategy for the rapid identification and validation of the direct targets of aconitine-induced cardiotoxicity, Drug Des. Devel. Ther. 15 (2021) 4649-4664.

[59] J. Lee, K.M. Song, C.H. Jung, Diosmin restores the skin barrier by targeting the aryl hydrocarbon receptor in atopic dermatitis, Phytomedicine. 81 (2021) 153418.

[60] J.J. Chen, Q.L. Yan, M. Bai, Q. Liu, S.J. Song, G.D. Yao, Deoxyelephantopin, a germacrane-type sesquiterpene lactone from Elephantopus scaber, induces mitochondrial apoptosis of hepatocarcinoma cells by targeting Hsp90alpha in vitro and in vivo, Phytother. Res. 37 (2023) 702-716.

[61] Y. Tu, L. Tan, T. Lu, K. Wang, H. Wang, B. Han, et al., Glytabastan B, a coumestan isolated from Glycine tabacina, alleviated synovial inflammation, osteoclastogenesis and collagen-induced arthritis through inhibiting MAPK and PI3K/AKT pathways, Biochem Pharmacol. 197 (2022) 114912.

[62] D. Zhu, C. Chen, X. Liu, S. Wang, J. Zhu, H. Zhang,et al., Osteosarcoma cell proliferation suppression via SHP-2-mediated inactivation of the JAK/STAT3 pathway by tubocapsenolide A, J. Adv. Res. 34 (2021) 79-91.

[63] E. Morretta, A. Tosco, C. Festa, M. Mozzicafreddo, M.C. Monti, A. Casapullo, Crellastatin A, a PARP-1 Inhibitor Discovered by Complementary Proteomic Approaches, Chem. Med. Chem. 15 (2020) 317-323.

[64] F. Chen, C. Li, H. Cao, H. Zhang, C. Lu, R. Li, et al., Identification of adenylate kinase 5 as a protein target of ginsenosides in brain tissues using mass spectrometry-based drug affinity responsive target stability (DARTS) and cellular thermal shift assay (CETSA) techniques, J. Agric. Food Chem. 70 (2022) 2741-2751.

[65] C. Park, S. Zhou, J. Gilmore, S. Marqusee, Energetics-based protein profiling on a proteomic scale: identification of proteins resistant to proteolysis, J. Mol. Biol. 368 (2007) 1426-1437.

[66] A. Holfeld, J.P. Quast, R. Bruderer, L. Reiter, N. de Souza, P. Picotti, Limited proteolysis-mass spectrometry to identify metabolite-protein interactions, Methods Mol. Biol. 2554 (2023) 69-89.

[67] M. Pepelnjak, N. de Souza, P. Picotti, Detecting protein-small molecule interactions using limited proteolysis-mass spectrometry (LiP-MS). Trends Biochem. Sci. 45 (2020) 919-920.

[68] S. Schopper, A. Kahraman, P. Leuenberger, Y. Feng, I. Piazza, O. Müller, et al., Measuring protein structural changes on a proteome-wide scale using limited proteolysis-coupled mass spectrometry, Nat. Protoc. 12 (2017) 2391-2410.

[69] F. Feng, W. Zhang, Y. Chai, D. Guo, X. Chen, Label-free target protein characterization for small molecule drugs: recent advances in methods and applications, J. Pharm. Biomed. Anal. 223 (2023) 115107.

[70] L. Zhang, S.Y. Yang, F.R. Qi-Li, X.X. Liu, W.T. Zhang, C. Peng, et al., Administration of isoliquiritigenin prevents nonalcoholic fatty liver disease through a novel IQGAP2-CREB-SIRT1 axis, Phytother. Res. 35 (2021) 3898-3915.
[71] S. Chen, X. Liu, C. Peng, C. Tan, H. Sun, H. Liu, et al. The phytochemical hyperforin triggers thermogenesis in adipose tissue via a Dlat-AMPK signaling axis to curb obesity, Cell Metab. 33 (2021) 565-580.
[72] W. Zhang, M. Wang, Z. Song, Q. Fu, J. Chen, W. Zhang, et al. Farrerol directly activates the deubiqutinase UCHL3 to promote DNA repair and reprogramming when mediated by somatic cell nuclear transfer, Nat. Commun. 14 (2023) 1838.
[73] N. Sheng, Z.H. Zhang, H. Zheng, C.Y. Ma, M.L. Li, Z. Wang, et al., Scutellarin rescued mitochondrial damage through ameliorating mitochondrial glucose oxidation via the Pdk-Pdc axis, Adv. Sci. 10 (2023) e2303584..
[74] K. Li, S. Chen, K. Wang, Y. Wang, L. Xue, Y. Ye, et al., A peptide-centric local stability assay enables proteome-scale identification of the protein targets and binding regions of diverse ligands, Nat. Methods. 22(2025) 278-282.
[75] I. Piazza, N. Beaton, R. Bruderer, T. Knobloch, C. Barbisan, et al., A machine learning-based chemoproteomic approach to identify drug targets and binding sites in complex proteomes, Nat. Commun. 11 (2020) 4200.
[76] D.M. Martinez, R. Jafari, M. Ignatushchenko, T. Seki, E.A. Larsson, C. Dan, et al., Monitoring drug target engagement in cells and tissues using the cellular thermal shift assay, Science. 341 (2013) 84-87.
[77] R. Jafari, H. Almqvist, H. Axelsson, M. Ignatushchenko, T. Lundback, P. Nordlund, et al., The cellular thermal shift assay for evaluating drug target interactions in cells, Nat. Protoc. 9 (2014) 2100-2122.
[78] A. Mateus, N. Kurzawa, I. Becher, S. Sridharan, D. Helm, F. Stein, et al., Thermal proteome profiling for interrogating protein interactions, Mol. Syst. Biol. 16 (2020) e9232.
[79] T.W. Sanchez, M.H. Ronzetti, A.E. Owens, M. Antony, T. Voss, E. Wallgren, et al., Real-time cellular thermal shift assay to monitor target engagement, ACS Chem. Biol. 17 (2022) 2471-2482.
[80]Y. Tu, L. Tan, H. Tao, Y. Li, H. Liu, CETSA and thermal proteome profiling strategies for target identification and drug discovery of natural products. Phytomedicine. (2023) 116.
[81] X. Zhou, S. Zhao, T. Liu, L. Yao, M. Zhao, X. Ye, et al. Schisandrol A protects

AGEs-induced neuronal cells death by allosterically targeting ATP6V0d1 subunit of V-ATPase, Acta. Pharm. Sin. B. 12 (2022) 3843-3860.
[82] T. Tezil, M. Chamoli, C.P. Ng, R.P. Simon, V.J. Butler, M. Jung, et al., Lifespan-increasing drug nordihydroguaiaretic acid inhibits p300 and activates autophagy, NPJ Aging Mech. Dis. 5 (2019) 7.
[83] Y. Huang, J. Fang, W. Lu, Z. Wang, Q. Wang, Y. Hou, et al. A systems pharmacology approach uncovers wogonoside as an angiogenesis inhibitor of triple-negative breast cancer by targeting hedgehog signaling, Cell Chem. Biol. 26 (2019) 1143-1158.
[84] W. Wang, Y. Wu, S. Chen, X. Liu, J. He, S. Wang, et al., Shikonin is a novel and selective IMPDH2 inhibitor that target triple-negative breast cancer. Phytother. Res. 35 (2021) 463-476.
[85] M. Yin, J. Dong, C. Sun, X. Liu, Z. Liu, L. Liu, et al., Raddeanin A enhances mitochondrial DNA-cGAS/STING axis-mediated antitumor immunity by targeting transactive responsive DNA-binding protein 43, Adv. Sci. 10 (2023) e2206737.
[86] X.Q. Yu, Q.L. Yan, S. Shen, Z.Y. Cheng, X.X. Huang, G.D. Yao, et al., Chamaejasmenin E from Stellera chamaejasme induces apoptosis of hepatocellular carcinoma cells by targeting c-Met in vitro and in vivo, Bioorg. Chem. 119 (2022) 105509.
[87] W. Shen, X.L. Hu, S.Y. Li, L. Li, X.W. Dong, H. Liu, et al., Pyranochromones with anti-inflammatory activities in arthritis from Calophyllum membranaceum, J. Nat. Prod. 85 (2022) 1374-1387.
[88] D. Xu, W. Zhao, Y. Feng, X. Wen, H. Liu, J. Ping, Pentoxifylline attenuates nonalcoholic fatty liver by inhibiting hepatic macrophage polarization to the M1 phenotype, Phytomedicine. 106 (2022) 154368.
[89] X.L. Hu, W. Shen, R. Wang, H. Long, Q. Wang, J.H. Feng, et al., Discovery of Eucalyptin C, derived from the fruits of Eucalyptus globulus Labill., as a novel selective PI3Kγ inhibitor for immunosuppressive treatment, Chin. J. Nat. Med. 19 (2021) 844-855.
[90] Y.C. Liu, N. Feng, W.W. Li, P.F. Tu, J.P. Chen, J.Y. Han, et al., Costunolide plays an anti-neuroinflammation role in lipopolysaccharide-induced BV2 microglial activation by targeting cyclin-dependent kinase 2, Molecules. 25 (2020) 2840.
[91] X.W. Zhang, N. Feng, Y.C. Liu, Q. Guo, J.K. Wang, Y.Z. Bai, et al.,

Neuroinflammation inhibition by small-molecule targeting USP7 noncatalytic domain for neurodegenerative disease therapy, Sci Adv. 8 (2022) 0789.

[92] H. Xiao, X. Sun, Z. Lin, Y. Yang, M. Zhang, Z. Xu, et al., Gentiopicroside targets PAQR3 to activate the PI3K/AKT signaling pathway and ameliorate disordered glucose and lipid metabolism, Acta Pharm. Sin. B. 12 (2022) 2887-2904.

[93] Y. Lu, J. Ji, S. Chu, F. Shen, W. Yang, W. Lei, M. et al., CaMKII, that binds with ligustilide, as a potential drug target of Suxiao jiuxin pill, a traditional Chinese medicine to dilate thoracic aorta, Clin. Transl. Med. 12 (2022) e907.

[94] R. Wang, X. Hu, J. Wang, L. Zhou, Y. Hong, Y. Zhang, et al., Proanthocyanidin A1 promotes the production of platelets to ameliorate chemotherapy-induced thrombocytopenia through activating JAK2/STAT3 pathway, Phytomedicine. 95 (2022) 153880.

[95] J. Zhong, X.F. He, X.X. Gao, Q.H. Liu, Y. Zhao, Y. Hong, et al. Hyodeoxycholic acid ameliorates nonalcoholic fatty liver disease by inhibiting RAN-mediated PPARa nucleus-cytoplasm shuttling, Nat. Commun. 14 (2023) 1275.

[96] J. Qu, B. Qiu, Y. Zhang, Y. Hu, Z. Wang, Z. Guan, et al., The tumor-enriched small molecule gambogic amide suppresses glioma by targeting WDR1-dependent cytoskeleton remodeling, Signal Transduct Target Ther. 8 (2023) 424.

[97] A. Mateus, T.A. Määttä, M.M. Savitski, Thermal proteome profiling: unbiased assessment of protein state through heat-induced stability changes, Proteome Sci. 15 (2016) 13.

[98] C. Le Sueur, H.M. Hammaren, S. Sridharan, M.M. Savitski, Thermal proteome profiling: insights into protein modifications, associations, and functions, Curr. Opin. Chem. Biol. 71 (2022) 102225.

[99] M.M. Savitski, F.B. Reinhard, H. Franken, T. Werner, M.F. Savitski, D. Eberhard, et al., Tracking cancer drugs in living cells by thermal profiling of the proteomem, Science. 346 (2014) 1255784.

[100] A. Mateus, N. Kurzawa, J. Perrin, G. Bergamini, M.M. Savitski, Drug target identification in tissues by thermal proteome profiling, Annu. Rev. Pharmacol. Toxicol. 62 (2022) 465-482.

[101] X.W. Zhang, N. Feng, L.C. Wang, D. Liu, Y.M. Hua, C. Zhang, et al., Small-molecule arone protects from neuroinflammation in LPS-activated microglia BV-2 cells by targeting histone-remodeling chaperone ASF1a, Biochem. Pharmacol. 177 (2020) 113932.

[102] C.P. Sun, J.J. Zhou, Z.L. Yu, X.K. Huo, J. Zhang, C. Morisseau, et al., Kurarinone alleviated Parkinson's disease via stabilization of epoxyeicosatrienoic acids in animal model, Proc. Natl. Acad. Sci U S A. 119 (2022) e2118818119.
[103] J. Kakegawa, S. Ohtsuka, M. Yokoyama, T. Hosoi, K. Ozawa, T. Hatanaka, Thermal proteome profiling reveals glutathione peroxidase 4 as the target of the autophagy inducer conophylline, Mol. Pharmacol. 100 (2021) 181-192.
[104] C. Yang, S. Zhu, Y. Chen, Z. Liu, W. Zhang, C. Zhao, et al., Flavonoid 4,4'-dimethoxychalcone suppresses cell proliferation via dehydrogenase inhibition and oxidative stress aggravation, Free Radic. Biol. Med. 175 (2021) 206-215.
[105] X.L. Zhang, Q. Wang, Y.N. Li, C.F. Ruan, S.Y. Wang, L.H. Hu, et al., Solvent-Induced protein precipitation for drug target discovery on the proteomic scale, Anal Chem. 92 (2020) 1363-1371.
[106] J.M. Li, H.S. Deng, Y.D. Yao, W.T. Wang, J.Q. Hu, Y. Dong, et al., Sinomenine ameliorates collagen-induced arthritis in mice by targeting GBP5 and regulating the P2X7 receptor to suppress NLRP3-related signaling pathways, Acta Pharmacologica Sinica. 44 (2023) 2504-2524.
[107] Z. Yu, J. Gao, X. Zhang, Y. Peng, W. Wei, J. Xu, et al., Characterization of a small-molecule inhibitor targeting NEMO/IKKβ to suppress colorectal cancer growth, Signal Transduct Target Ther. 7 (2022) 71.
[108] X.L. Zhang, K.Y. Wang, S.J. Wu, C.F. Ruan, K.J. Li, Y. Wang, et al., Highly effective identification of drug targets at the proteome level by pH-dependent protein precipitation, Chem Sci. 13 (2022) 12403-12418.
[109] X. Zhang, C. Ruan, Y. Wang, K. Wang, X. Liu, J. Lyu, M. Ye, Integrated Protein Solubility Shift Assays for Comprehensive Drug Target Identification on a Proteome-Wide Scale, Anal Chem. 95 (2023) 13779-13787.
[110] G.M. West, L. Tang, M.C. Fitzgerald, Thermodynamic analysis of protein stability and ligand binding using a chemical modification- and mass spectrometry-based strategy, Anal Chem. 80 (2008) 4175-4185.
[111] E.C. Strickland, M.A. Geer, D.T. Tran, J. Adhikari, G.M. West, P.D. DeArmond, et al., Thermodynamic analysis of protein-ligand binding interactions in complex biological mixtures using the stability of proteins from rates of oxidation, Nat. Protoc, 8 (2013) 148-161.
[112] H. Meng, M.C. Fitzgerald. Proteome-wide characterization of phosphorylation-induced conformational changes in breast cancer, J. Proteome Res. 17 (2018)

1129-1137.
[113] F. Baud, S. Karlin, Measures of residue density in protein structures, Proc. Natl. Acad. Sci U S A. 96 (1999) 12494-12499.
[114] U. Kaur, H. Meng, F. Lui, R. Ma, R.N. Ogburn, J.H.R. Johnson, et al., Proteome-wide structural biology: an emerging field for the structural analysis of proteins on the proteomic scale, J. Proteome. Res. 17 (2018) 3614-3627.
[115] B.F. Cravatt, A.T. Wright, J.W. Kozarich, Activity-based protein profiling: from enzyme chemistry to proteomic chemistry, Annu. Rev. Biochem. 77 (2008) 383-414.
[116] S. Wang, Y. Tian, M. Wang, M. Wang, G.B. Sun, X.B. Sun, Advanced activity-based protein profiling application strategies for drug development. Front Pharmacol. 9 (2018) 353.
[117] H.J. Benns, C.J. Wincott, E.W. Tate, M.A. Child, Activity- and reactivity-based proteomics: recent technological advances and applications in drug discovery, Curr. Opin. Chem. Biol. 60 (2021) 20-29.
[118] X. Chen, Y.K. Wong, J. Wang, J. Zhang, Y.M. Lee, H.M. Shen, et al., Target identification with quantitative activity based protein profiling (ABPP). Proteomics. 17 (2017) 3-4.
[119] L.I. Willems, H.S. Overkleeft, S.I. van Kasteren, Current developments in activity-based protein profiling, Bioconjug. Chem. 25 (2014) 1181-1191.
[120] J. Wei, X. Chen, Y. Li, R. Li, K. Bao, L. Liao, et al., Cucurbitacin B-induced G2/M cell cycle arrest of conjunctival melanoma cells mediated by GRP78-FOXM1-KIF20A pathway, Acta. Pharm. Sin. B. 12 (2022) 3861-3876.
[121] J.L. Huang, X.L. Yan, W. Li, R.Z. Fan, S. Li, J. Chen, et al., Discovery of highly potent daphnane diterpenoids uncovers importin-β1 as a druggable vulnerability in castration-resistant prostate cancer, J. Am. Chem. Soc. 144 (2022) 17522-17532.
[122] P. Luo, D. Liu, Q. Zhang, F. Yang, Y.K. Wong, F. Xia, et al., Celastrol induces ferroptosis in activated HSCs to ameliorate hepatic fibrosis via targeting peroxiredoxins and HO-1, Acta. Pharm. Sin. B. 12 (2022) 2300-2314.
[123] C. Chen, T. Zhu, X. Liu, D. Zhu, Y. Zhang, S. Wu, et al., Identification of a novel PHGDH covalent inhibitor by chemical proteomics and phenotypic profiling, Acta Pharm Sin B. 12 (2022) 246-261.
[124] C. Chen, L. Gong, X. Liu, T. Zhu, W. Zhou, L. Kong, et al., Identification of peroxiredoxin 6 as a direct target of withangulatin A by quantitative chemical

proteomics in non-small cell lung cancer, Redox Biol. 46 (2021) 102130.
[125] W.S. Zhao, K.F. Chen, M. Liu, X.L. Jia, Y.Q. Huang, B.B. Hao, et al., Investigation of targets and anticancer mechanisms of covalently acting natural products by functional proteomics, Acta. Pharmacol. Sin. 44 (2023) 1701-1711.
[126] Z.G. Zheng, Y.Y. Xu, W.P. Liu, Y. Zhang, C. Zhang, H.L. Liu, et al., Discovery of a potent allosteric activator of DGKQ that ameliorates obesity-induced insulin resistance via the sn-1,2-DAG-PKCepsilon signaling axis, Cell Metab. 35 (2023) 101-117.
[127] S. Huang, F.J. Wang, H. Lin, T. Liu, C.X. Zhao, L.G. Chen,Affinity-based protein profiling to reveal targets of puerarin involved in its protective effect on cardiomyocytes, Biomed. Pharmacother. 134 (2021) 111160.
[128] Q. Zhang, P. Luo, F. Xia, H. Tang, J. Chen, J. Zhang, et al., Capsaicin ameliorates inflammation in a TRPV1-independent mechanism by inhibiting PKM2-LDHA-mediated Warburg effect in sepsis, Cell Chem. Biol. 29 (2022) 1248-1259.
[129] X. Dong, S. Lu, Y. Tian, H. Ma, Y. Wang, X. Zhang, et al., Bavachinin protects the liver in NAFLD by promoting regeneration via targeting PCNA, J Adv. Res. 55 (2023) 131-144.
[130] W.J. Luo, F.C.F. Ip, G.M. Fu, K. Cheung, Y. Tian, Y.Q. Hu, et al., A pentacyclic triterpene from targets γ-Secretase, Acs. Chem. Neurosci. 11 (2020) 2827-2835.
[131] M.W. Harding, A. Galat, D.E. Uehling, S.L. Schreiber, A receptor for the immunosuppressant FK506 is a cis-trans peptidyl-prolyl isomerase, Nature. 341 (1989) 758-760.
[132] U. Rix, G. Superti-Furga, Target profiling of small molecules by chemical proteomics, Nat Chem Biol. 5 (2009) 616-624.
[133] Y. Liu, M. Guo, Chemical proteomic strategies for the discovery and development of anticancer drugs, Proteomics. 14 (2014) 399-411.
[134] J. Li, T. Casteels, T. Frogne, C. Ingvorsen, C. Honore, M. Courtney, et al., Artemisinins target GABAA receptor signaling and impair α cell identity, Cell. 168 (2017) 86-100.
[135] J. Wang, J. Wu, X. Li, H. Liu, J. Qin, Z. Bai, et al., Identification and validation nucleolin as a target of curcumol in nasopharyngeal carcinoma cells, J. Proteomics. 182 (2018) 1-11.
[136] Y. Tian, N. Wan, H.Q. Zhang, C. Shao, M. Ding, Q.Y. Bao, et al., Chemoproteomic mapping of the glycolytic targetome in cancer cells, Nat. Chem.

Biol. 19 (2023) 1480-1491.
[137] G.L. Deng, L.S. Zhou, B.L. Wang, X.F. Sun, Q.C. Zhang, H.Q. Chen, et al., Targeting cathepsin B by cycloastragenol enhances antitumor immunity of CD8 T cells via inhibiting MHC-I degradation, J. Immunother. Cancer. 10 (2022) e004874.
[138] Y. Zhu, N. Wan, X. Shan, G. Deng, Q. Xu, H. Ye, et al., Celastrol targets adenylyl cyclase-associated protein 1 to reduce macrophages-mediated inflammation and ameliorates high fat diet-induced metabolic syndrome in mice, Acta. Pharm. Sin. B. 11 (2021) 1200-1212.
[139] W.C. Yan, D.X. Wang, N. Wan, S. Wang, C. Shao, H.Q. Zhang, et al., Living cell-target responsive accessibility profiling reveals silibinin targeting ACSL4 for combating ferroptosis, Anal. Chem. 94 (2022) 14820-14826.
[140] M. Zhao, L. Zhou, Q. Zhang, M. Wang, Y. Dong, Y. Wang, et al. Targeting MAPK14 by Lobeline Upregulates Slurp1-Mediated Inhibition of Alternative Activation of TAM and Retards Colorectal Cancer Growth, Adv. Sci. 22 (2025) e2407900.
[141] Z.Y. Liu, S.R. Yang, L.Q. Zhou, M. He, Y. Bai, S. Zhao, et al., Structural characterization of protein-material interfacial interactions using lysine reactivity profiling-mass spectrometry, Nat. Protoc. 18 (2023) 2600-2623.
[142] Z.Y. Liu, Y. Zhou, J. Liu, J. Chen, A.J.R. Heck, F.J. Wang, Reductive methylation labeling, from quantitative to structural proteomics, Trac-Trend Anal Chem. 118 (2019) 771-778.
[143] S.R. Yang, W.X. Zhang, Z.Y. Liu, Z.Y. Zhai, X.D. Hou, P. Wang, et al., Lysine reactivity profiling reveals molecular insights into human serum albumin-small-molecule drug interactions, Anal Bioanal Chem. 413 (2021) 7431-7440.
[144] Y. Zhou, Z. Liu, J. Zhang, T. Dou, J. Chen, G. Ge, et al., Prediction of ligand modulation patterns on membrane receptors via lysine reactivity profiling, Chem. Comm. 55 (2019) 4311-4314.
[145] Y. Zhou, Y. Wu, M.D. Yao, Z.Y. Liu, J. Chen, J. Chen, et al., Probing the lysine proximal microenvironments within membrane protein complexes by active dimethyl labeling and mass spectrometry, Anal Chem. 88 (2016) 12060-12065.
[146] M.M. Liu, W.C. Van Voorhis, R.J. Quinn, Development of a target identification approach using native mass spectrometry, Sci. Rep. 11 (2021) 2387.
[147] D.R. Littler, M. Liu, J.L. McAuley, S.A. Lowery, P.T. Illing, B.S. Gully, et al., A

natural product compound inhibits coronaviral replication in vitro by binding to the conserved Nsp9 SARS-CoV-2 protein, J. Biol. Chem. 297 (2021) 101362.

[148] H. Vu, L. Pedro, T. Mak, B. McCormick, J. Rowley, M. Liu, et al., Fragment-based screening of a natural product library against 62 potential malaria drug targets employing native mass spectrometry, ACS Infect. Dis. 4 (2018) 431-444.

[149] G.T. Nguyen, J.L. Bennett, S. Liu, S.E. Hancock, D.L. Winter, D.J. Glover and W.A. Donald, Multiplexed screening of thousands of natural products for protein-ligand binding in native mass spectrometry, J. Am. Chem. Soc. 143 (2021) 21379-21387.

[150] Q. Meng, Y.L. Song, C. Zhou, H. He, N. Zhang, H. Zhou, A hydrogen-deuterium exchange mass spectrometry-based protocol for protein-small molecule interaction analysis, Biophys Rep. 9 (2023) 99-111.

[151] F. Meissner, J.G. McAlister, M. Mann, M. Bantscheff, The emerging role of mass spectrometry-based proteomics in drug discovery, Nat Rev Drug Discov. 21 (2022) 637-654.

[152] J.G. Van Vranken, J.M. Li, D.C. Mitchell, J. Navarrete-Perea, S.P. Gygi, Assessing target engagement using proteome-wide solvent shift assays, Elife. 10 (2021) e70784.

[153] Chen, F., Li, C., Cao, H., Zhang, H., Lu, C., Li, R., et al. Identification of Adenylate Kinase 5 as a Protein Target of Ginsenosides in Brain Tissues Using Mass Spectrometry-Based Drug Affinity Responsive Target Stability (DARTS) and Cellular Thermal Shift Assay (CETSA) Techniques, J Agric Food Chem. 70 (2022) 2741-2751.

[154] Luo, L., Zhou, J., Liu, X., Chen, Y., Du, X., Gao, L., et al. Development of modern Chinese medicine guided by molecular compatibility theory. J. Adv. Res. (2024).

[155] J.L. Bennett, G.T. HNguyen,W.A. Donald. Protein-Small molecule interactions in native mass spectrometry, Chem Rev. 122 (2022) 7327-7385.

[156] M. Xu, J. Deng, K. Xu, T. Zhu, L. Han,Y. Yan, et al., In-depth serum proteomics reveals biomarkers of psoriasis severity and response to traditional Chinese medicine, Theranostics. 9 (2019) 2475-2488.

Figure 1. Fishing out target proteins from complex cell millieu.

Figure 2. Challenges and opportunities for the discovery of TCM target proteins.

Figure 3.TCM-MPs target fishing strategy combined with bio-layer interferometry reverse fishing technology to screen out GNAS key proteins that directly interact with active ingredients (Formononetin, Gallic acid, Ginsenoside F2 and Catalpol) from complex target protein systems.

Figure 4. Workflow diagram of proteolysis resistance approach for target identification. (A) drug affinity responsive target stability (DARTS). (B) limited proteolysis-mass spectrometry (LiP-MS). (C) peptide-centric local stability assay (PELSA).

Figure 5. Workflow diagram of denaturation resistance approach for target identification. (A) thermal proteome profiling (TPP) and cellular thermal shift assay (CETSA). (B) solvent-induced protein precipitation (SIP). (C) pH-dependent protein precipitation (pHDPP). (D) integrated protein solubility shift assay (IPSSA). (E) protein stability determinations from rates of oxidation (SPROX).

Figure 6.Workflow diagram of chemical probe approach for target identification. (A) activity-based protein profiling (ABPP) and compound-centric chemical proteomics (CCCP). (B) target responsive accessibility profiling (TRAP) and lysine reactivity profiling (LRP).

Figure 7. Workflow diagram of Native-MS for target identification.

Figure 8. Proteomics techniques have emerged as a cornerstone in early-stage drug discovery

Figure 1

Click here to access/download;Figure;figure1.tif

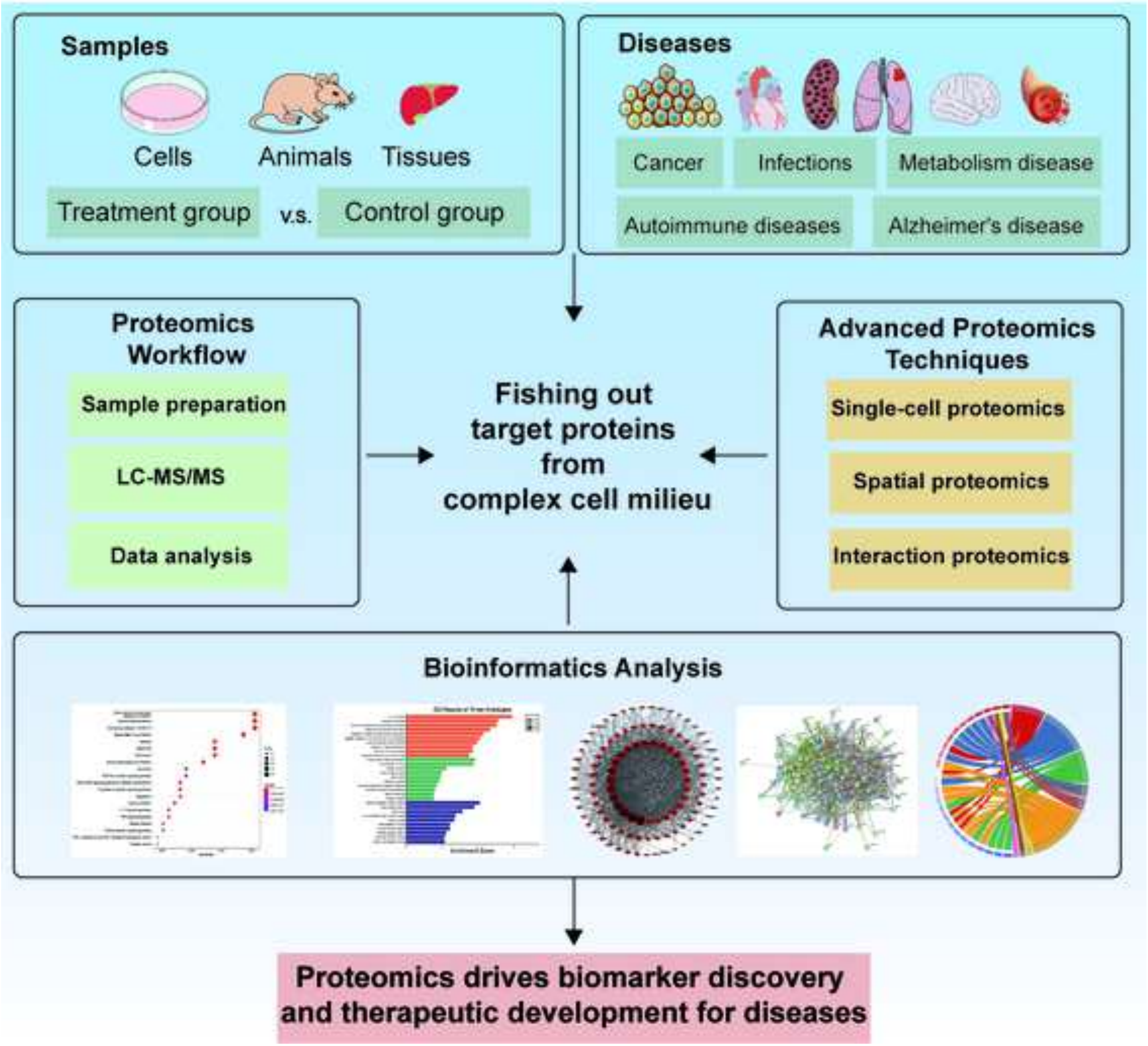

Figure 2

Click here to access/download;Figure;figure2.tif

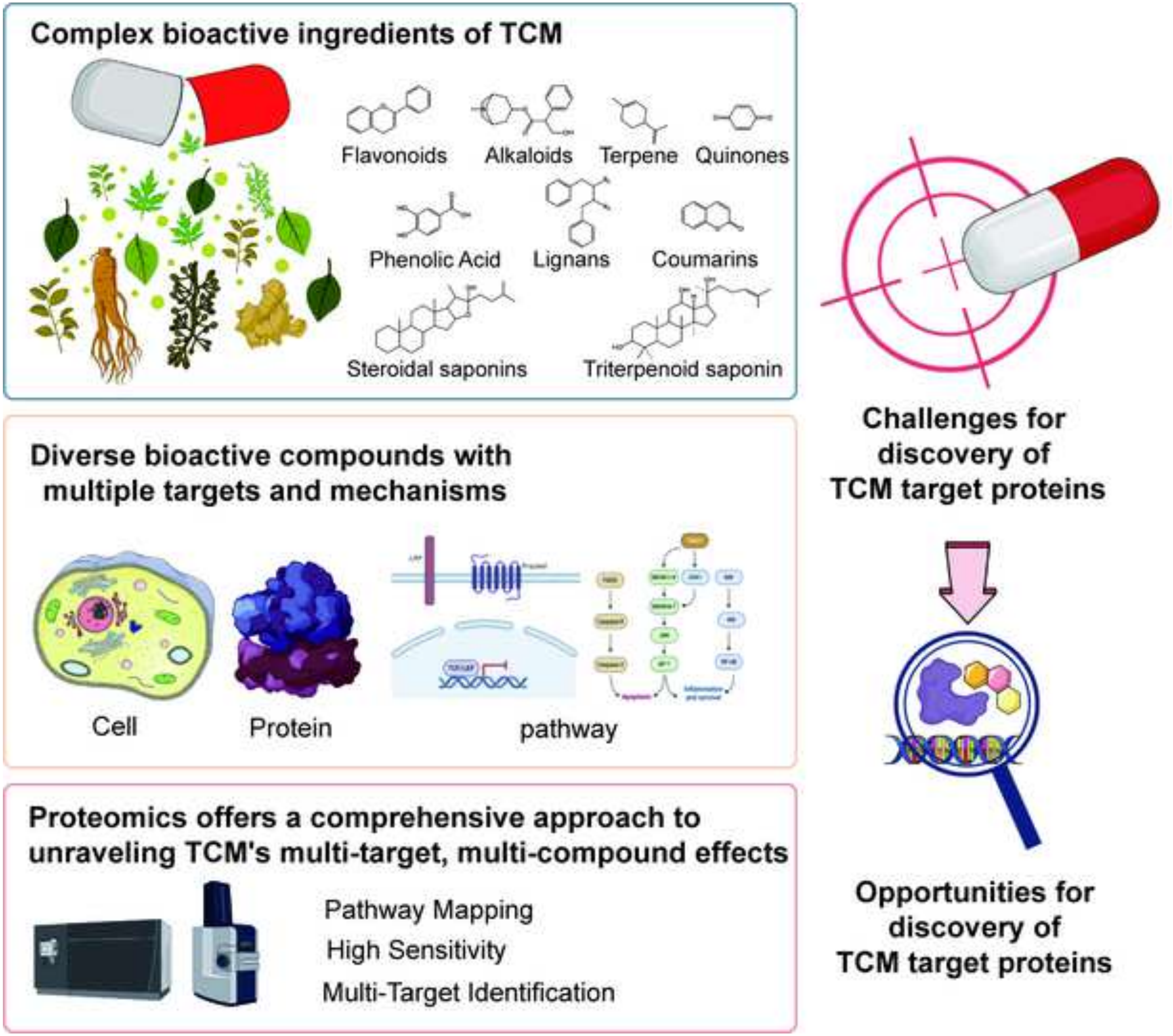

Figure 3-revised

Click here to access/download;Figure;figure3-revised.tif

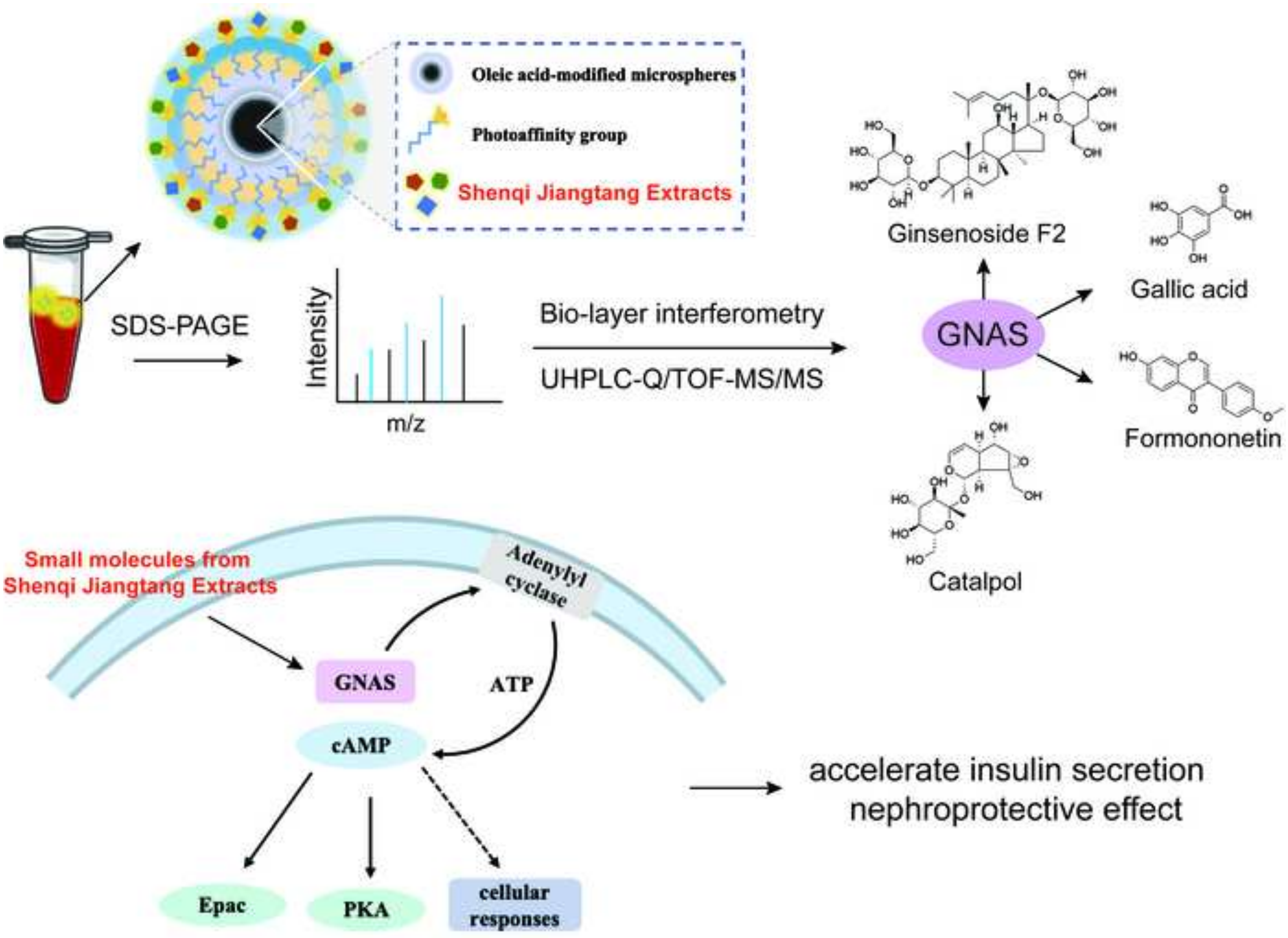

Click here to access/download;Figure;figure4-revised.tif

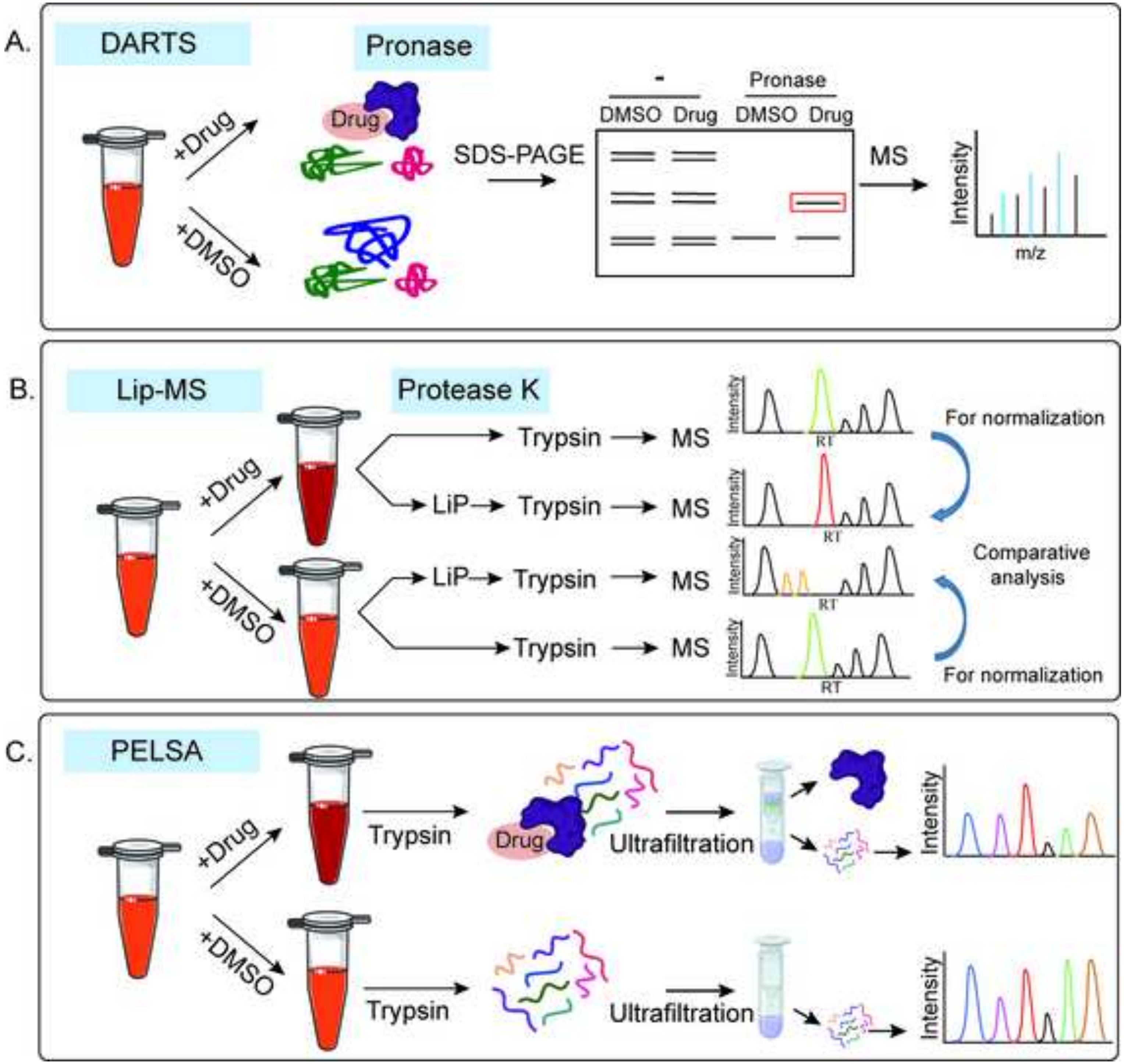

Figure 5-revised

Click here to access/download;Figure;figure5-revised.tif

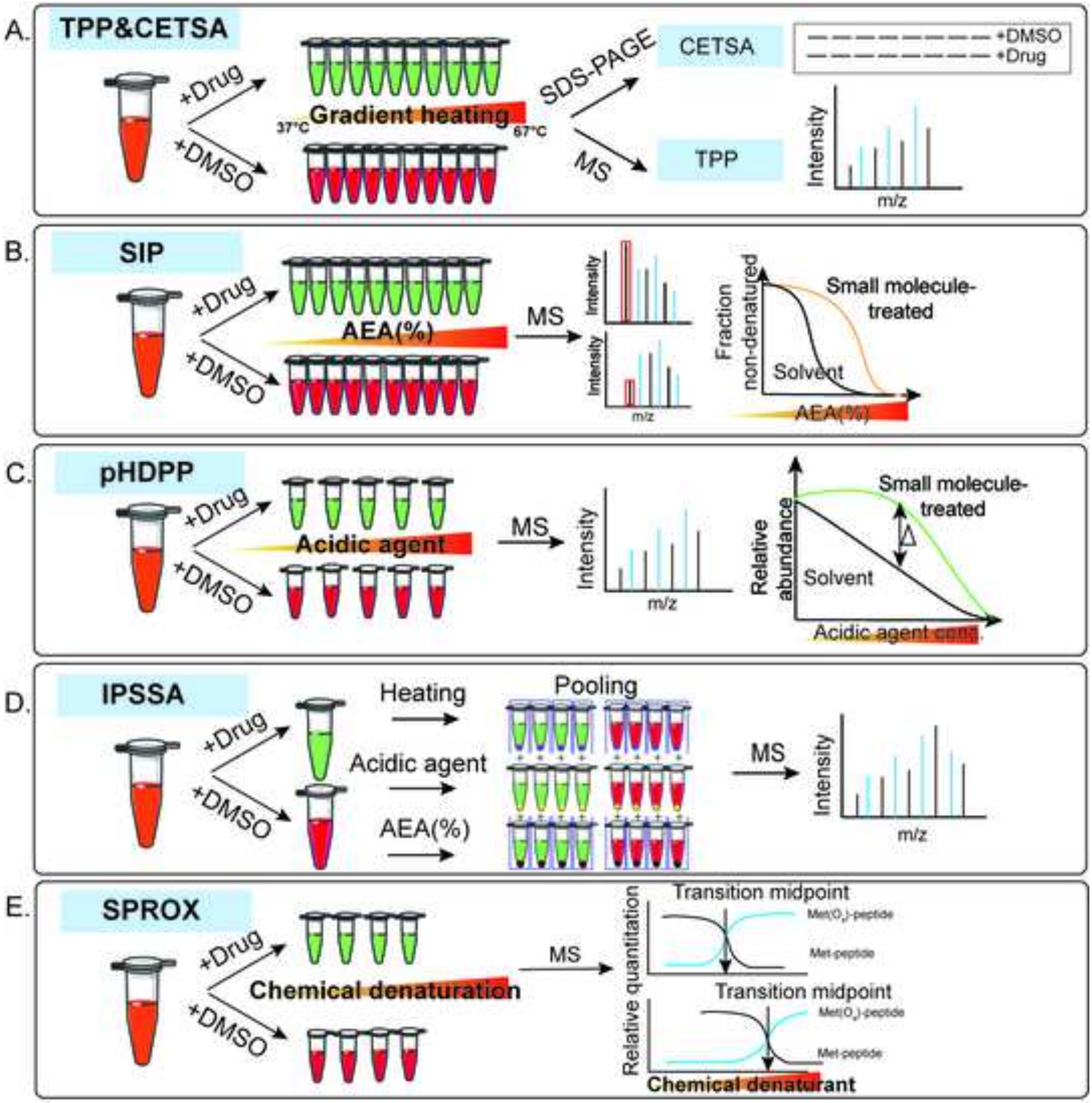

Figure 6-revised
Click here to access/download;Figure;figure6-revised.tif

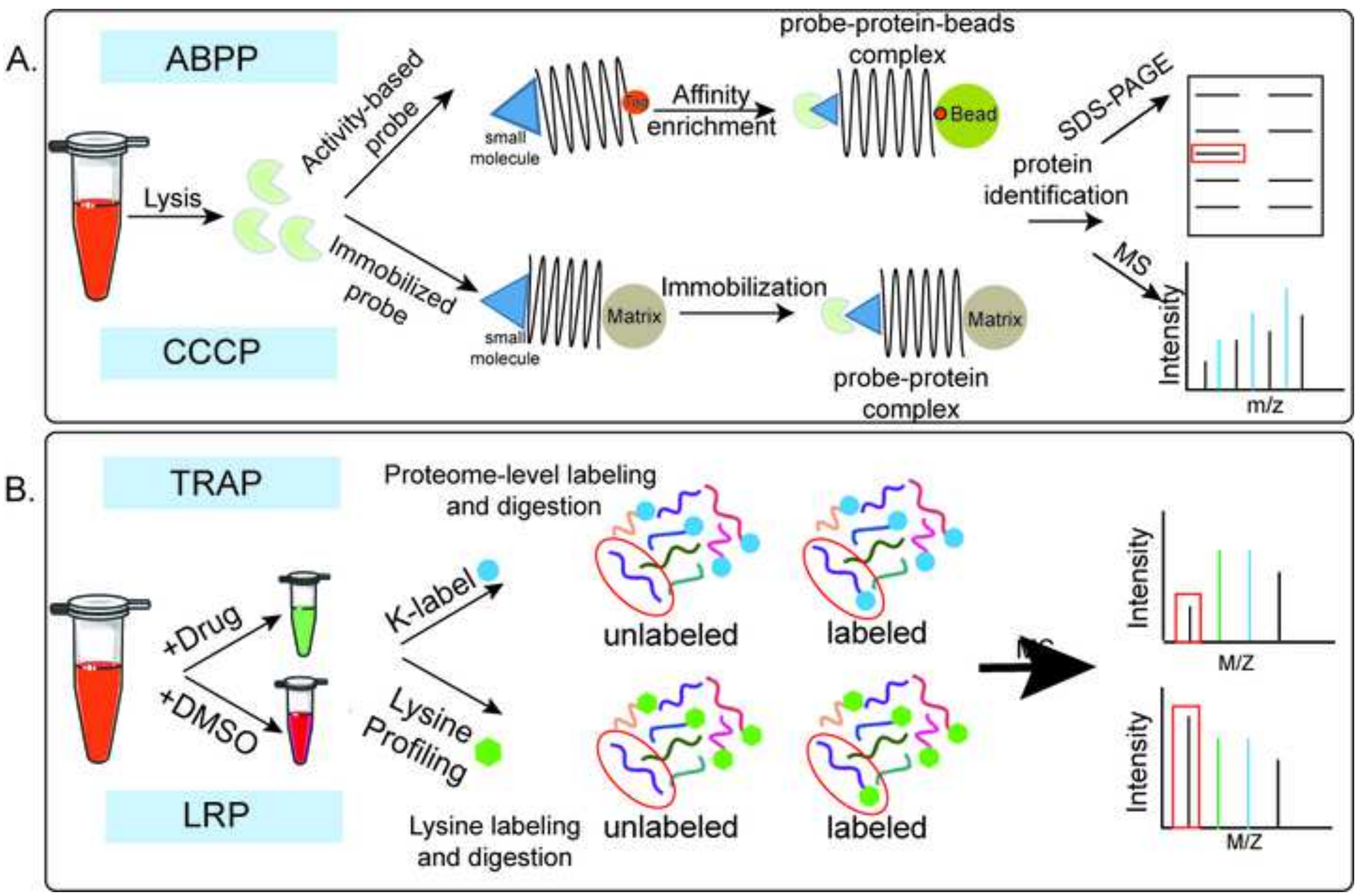

Click here to access/download;Figure;figure7-revised.tif

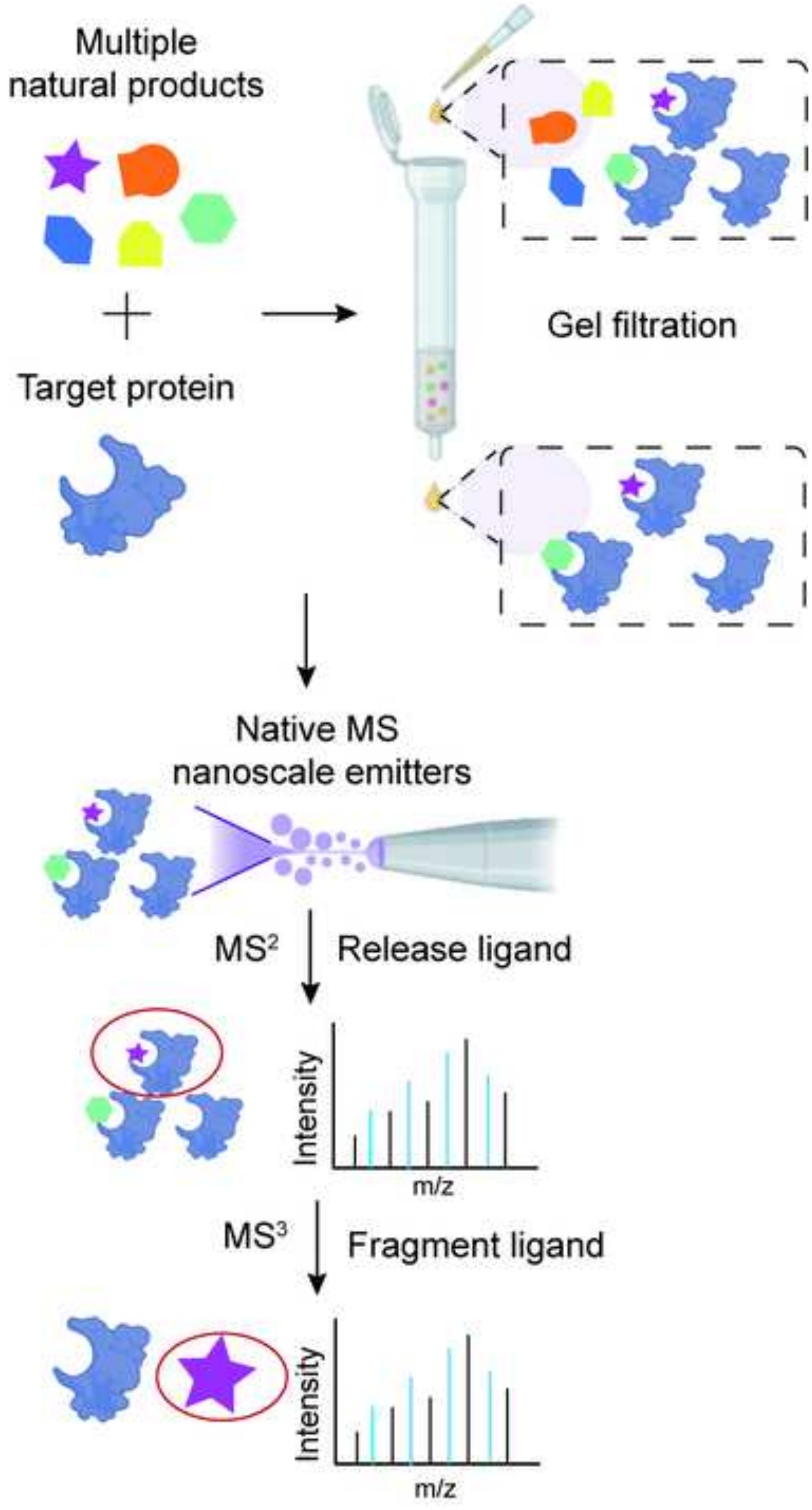

Figure 8-revised

Click here to access/download;Figure;figure8-revised.tif

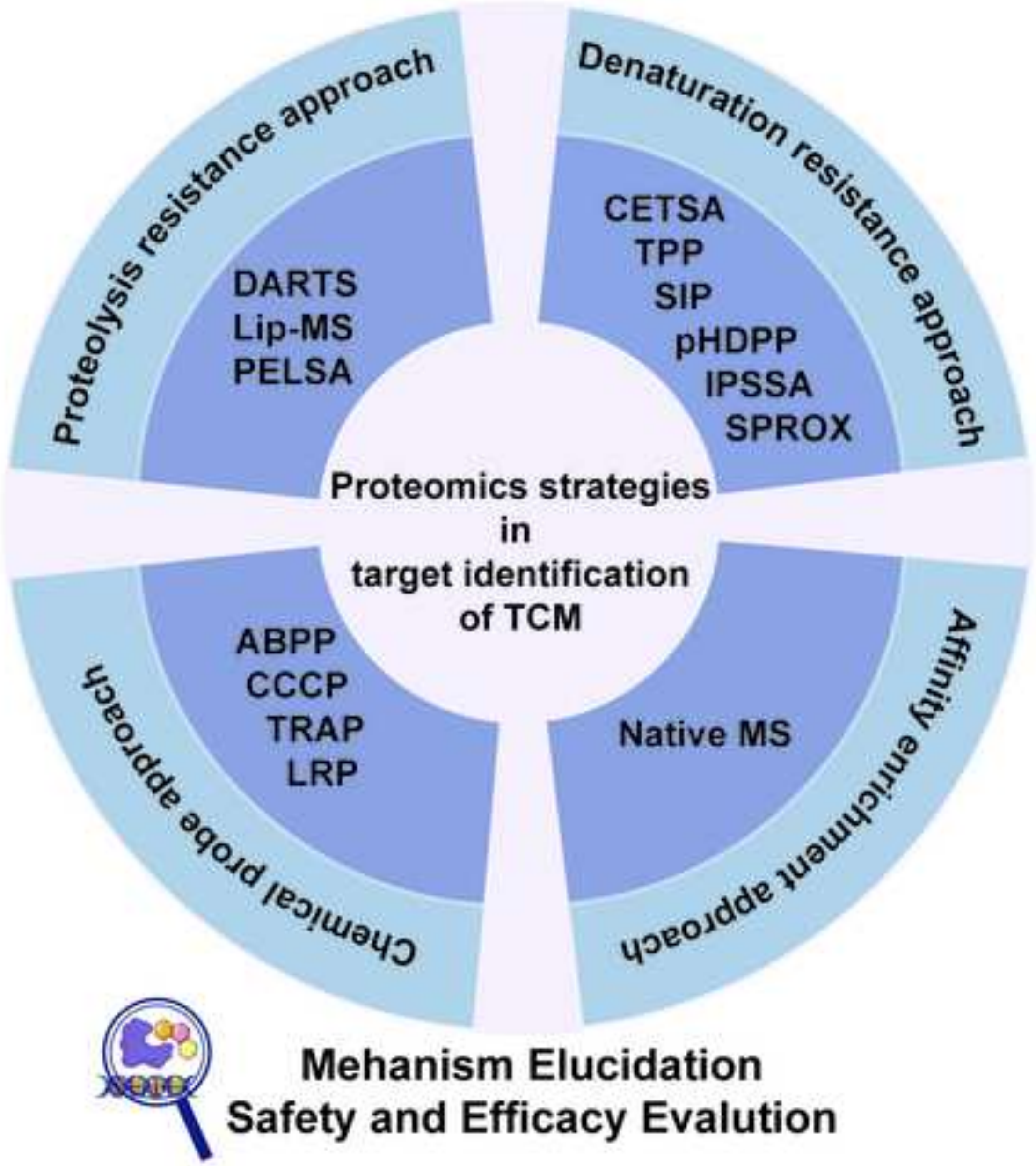

Table Click here to access/download;Table;Table.docx

Table 1. Proteolysis resistance approach for target identification and validation of TCMs.

Proteomic strategies	Compounds	Protein Target	Cell model/ animal model	Ref
DARTS	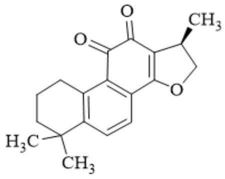 Cryptotanshinone	FKBP1A	HaCaT cells	44
	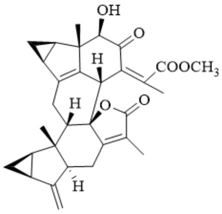 Shizukaol A	HMGB1	RAW 264.7 cells	52
	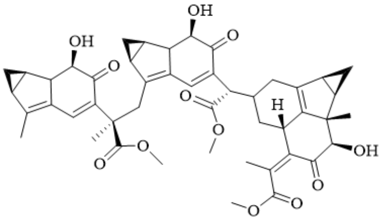 Trishizukaol A	TRAF6	RAW 264.7 cells	53
	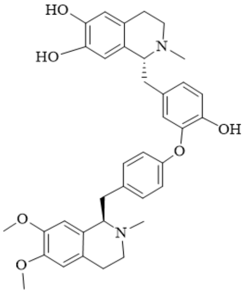 Daurisoline	HSP90	A549 and Hop62 cells	54
	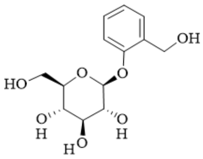 Salicin	IRE1α	Primary rat articular chondrocytes	55
	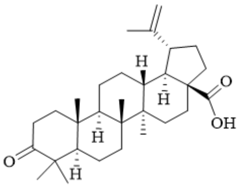 Liquidambaric acid	TRAF2	HCT116 cells	56
	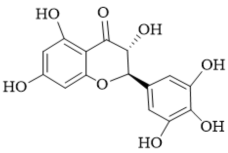 Dihydromyricetin	GRP78	3T3-L1 cells	57

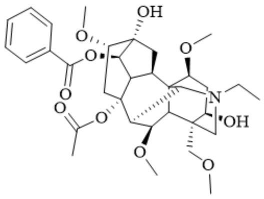 Aconitine	cPLA2	H9c2 cells	58
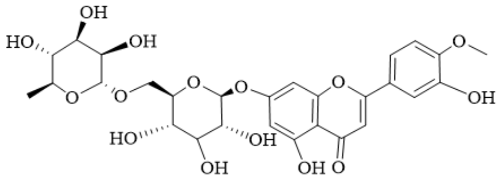 Diosmin	AhR	Normal human epidermal keratinocytes	59
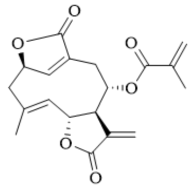 Deoxyelephantopin	Hsp90α	HepG2 and Hep3B cells	60
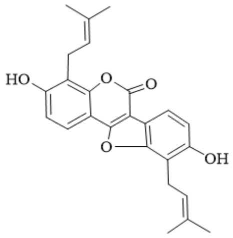 Glytabastan B	ERK2, JNK1 and PI3K catalytic subunit p110 (α and β)	SW982 cells	61
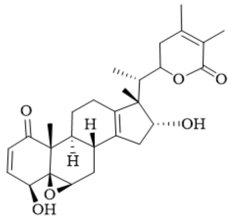 Tubocapsenolide A	SHP-2	U2OS cells	62
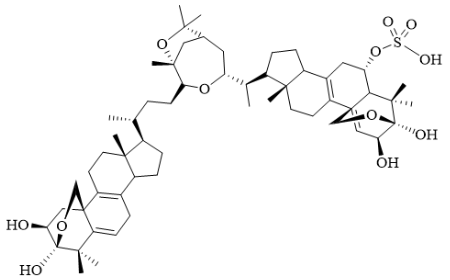 Crellastatin A	PARP-1	HeLa cells	63
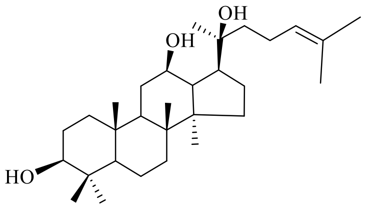 20(S)-protopanaxadiol	AK5	Brain tissues	64

LiP-MS	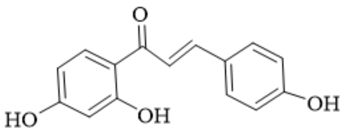 Isoliquiritigenin	IQGAP2	HepG2 cells	70
	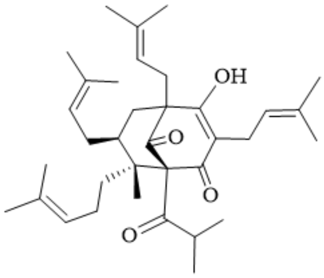 Hyperforin	Dlat	C3H10T1/2 cells	71
	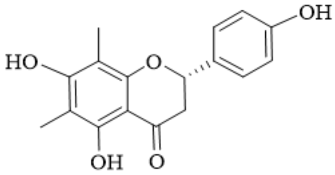 Farrerol	UCHL3	HEK293 cells	72
	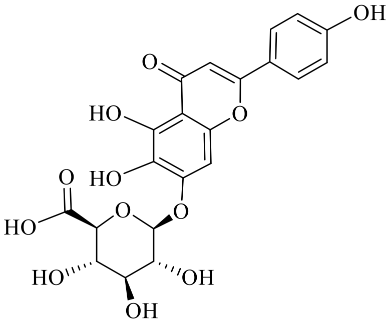 Scutellarin	PDK2	SK-N-SH cells	73

Table 2. Denaturation resistance approach for target identification and validation of TCMs.

Proteomic strategies	Compounds	Protein Target	Cell model/animal model	Ref
CETSA	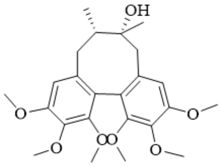 Schisandrol A	ATP6V0D1	PC12 cells	81
	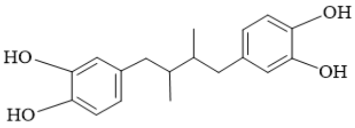 Nordihydroguaiaretic acid	P300	HEK293T cells	82
	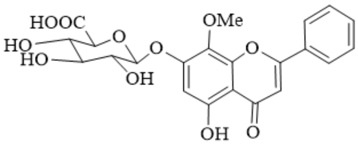 Wogonoside	Gli1	MDA-MB-231 cells	83
	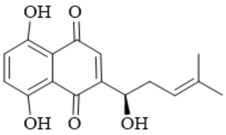 Shikonin	IMPDH2	MDA-MB-231 and 4T1 cells	84
	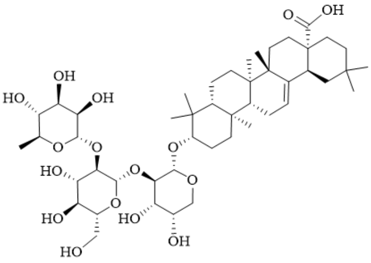 Raddeanin A	TDP-43	MC38 and B16 cells	85
	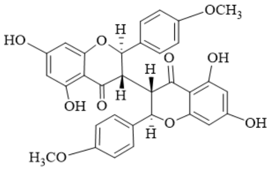 Chamaejasmenin E	receptor tyrosine kinase c-Met	Hep3B cells	86
	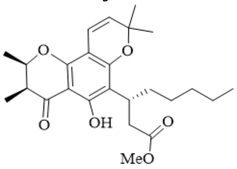 Calomembranone G	TLR4	RAW 264.7 cells	87
	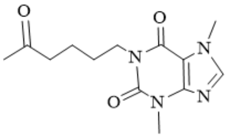 Pentoxifylline	TLR4	RAW 264.7 cells	88

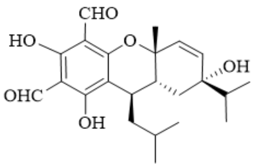 Eucalyptin C	PI3Kγ	Primary spleen cells	89
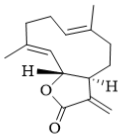 Costunolide	CDK2	BV-2 cells	90
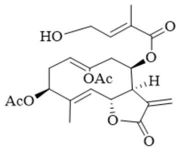 Eupalinolide B	USP7	BV-2 cells	91
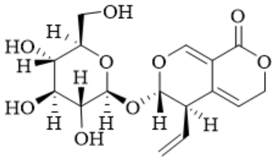 Gentiopicroside	PAQR3	HEK 293T cells	92
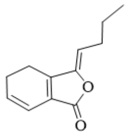 Ligustilide	CaMKII	VSMCs	93
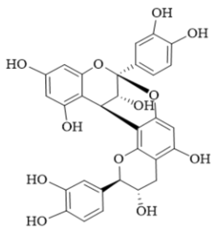 Proanthocyanidin A1	JAK2	Dami cells	94
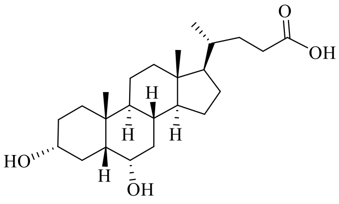 Hyodeoxycholic acid	RAN	AML12 cells	95
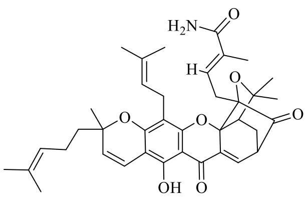 Gambogic amide	WDR1	T2-4 cells and 293 cells	96

TPP	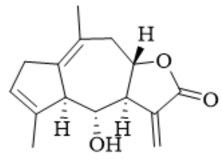<br>Artone	ASF1α	BV-2 cells	101
	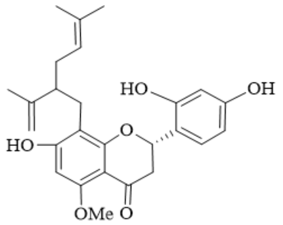<br>Kurarinone	soluble epoxide hydrolase enzyme	MPTP induced PD mice	102
	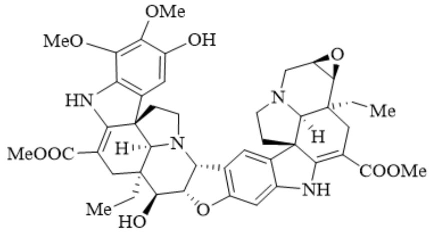<br>Conophylline	GPX4	U-2OS cells	103
	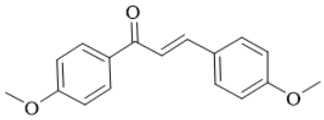<br>4,4′-dimethoxychalcone	ALDH1A3	A549 cells	104
SIP	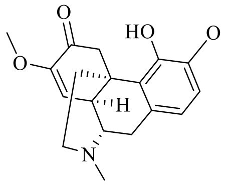<br>Sinomenine	GBP5	RAW264.7 cells	106
	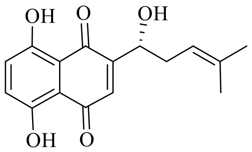<br>Shikonin	NEMO/IKKβ	LoVo cells	107
pHDPP	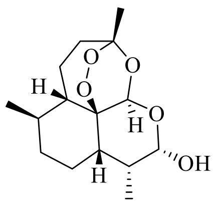<br>Dihydroartemisinin	ALDH7A1, HMGB1	Hela cells	108

Table 3. Chemical reactivity approach for target identification and validation of TCMs.

Proteomic strategies	Compounds	Protein Target	Cell model/ animal model	Ref
ABPP	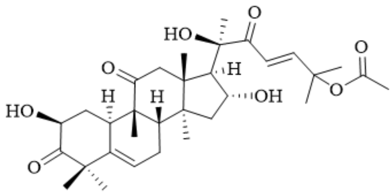 Cucurbitacin B	GRP78	conjunctival melanoma cells	120
	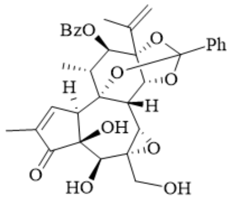 Daphnane diterpenoid DD1	Importin-β1	CRPC cells	121
	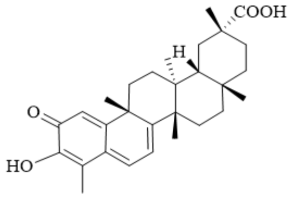 Celastrol	PRDXs	LX-2 cells	122
	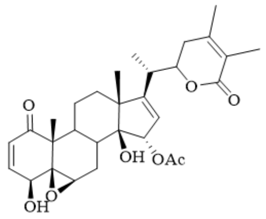 Withangulatin A	PHGDH	HCT-116 cells	123
	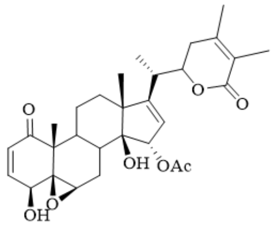 Withangulatin A	PRDX6	H1975 cells	124
	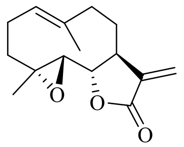 Parthenolide	USP10	MDA-MB-231 cells	125

	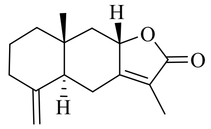<br>Atractylenolide II	DGKQ	HepG2 cells	126
	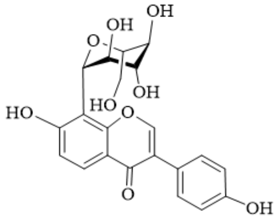<br>Puerarin	CHAF1B	AC16 cell line	127
	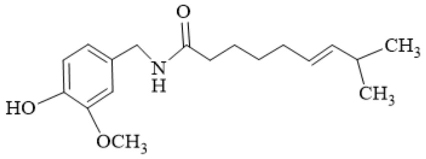<br>Capsaicin	PKM2<br>LDHA	RAW264.7 cells	128
	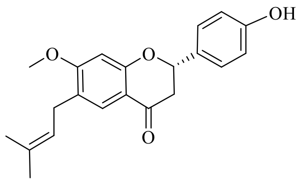<br>Bavachinin	PCNA	HepG2 cells	129
CCCP	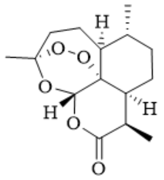<br>Artemisinin	Gephyrin protein	MIN6 cells	166
	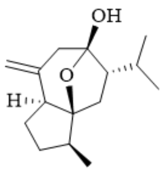<br>Curcumol	Nucleolin	NPC cells	167
TRAP	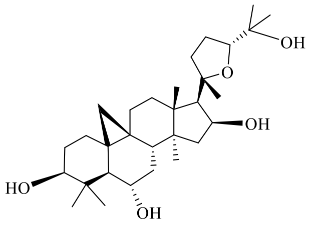<br>Cycloastragenol	CTSB	MC38 cells	137

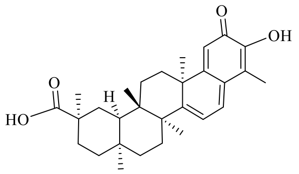<br>Celastrol	CAP1	THP-1 cells	138
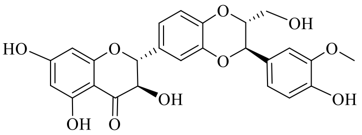<br>Silibinin	ACSL4	HepG2 cells	139
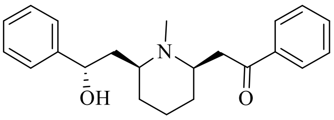<br>Lobeline	MAPK14	MC38 cells	140

Table 4. Advantages and limitations between different approach for target identification.

Approach	Advantages	limitations/Disadvantages	Ref
DARTS	1) Without chemical structural modification of biomolecule;<br>2) Simpler operation and higher throughput;<br>3) Suitable for those drugs causing target protein degradation	1) Not applicable for the drugs with structure change after entering cells;<br>2) Limits on the detection of low abundance target protein due to the low recovery of peptides from gel digestion;<br>3) Limits on those target proteins difficult to digest by protease	50-51, 65
Lip-MS	Without chemical structural modification of the biomolecule	1) Limits on the detection of low abundance target proteins;<br>2) Limits on the target proteins without proper MS detectable peptides in the binding sites;<br>3) Limits on the target proteins with conformational changes that hamper binding site identification	67-69
PELSA	1) Higher sensitivity<br>2) Without chemical structural modification of the biomolecule<br>3) Direct ligand binding-site determination<br>4) Ligand-induced local stability shifts	1) Need amount of trypsin;<br>2) Higher cost	74
CESTA	1) Direct biophysical studies in intact cells;<br>2) Suitable for the validation of target proteins identified by other methods	1) Not applicable for unknown target protein discovery;<br>2) Affect permeability of cell membrane;<br>3) The melting curve cannot be reproduced;<br>4) Not suitable for highly inhomogenous proteins;<br>5) Not recognize the binding site	77-80
TPP	1) Without chemical structural modification of biomolecule;<br>2) Suitable for the drugs with structure change after entering cells;	1) Higher cost;<br>2) Low transmembrane protein extraction	98-100
SIP	Without chemical structural modification of the biomolecule	Not adopt sample multiplexing for quantification of complete melting curves on a proteome-wide scale	105, 174
pHDPP	1) Work for multiple ligands;<br>2) 2) high sensitivity;<br>3) High complementarity to other proteomic approaches	Acidic agent may shift the acid-base equilibrium for some small molecule drugs	108
IPSSA	1) Without chemical structural modification of biomolecule;<br>2) Easy operationation	1) Limits on the detection of low abundance target protein;<br>2) Unable to distinguish direct/indirect effects	109
SPROX	Detect temperature and enzyme insensitive proteins	1) Need higher concentration of compound treatment;<br>2) Detect only the target proteins of highly selective methionine oxidation reaction	111-114

ABPP	1) Identify the activation state of target proteins;<br>2) Achieve the fast, sensitive, and selective identification of enzyme activity and inhibitors	1) Low detection sensitivity for low abundance protein targets;<br>2) Probe synthesis affects pharmacological activities of drugs;<br>3) Require chemical modication of compounds	117-119
CCCP	1) Higher selectivity to reduce the diversity of target protein complex;<br>2) Identify targets with no enzymatic function	1) Nonspecifically binding proteins lead to potential false-positive results;<br>2) Not easy to immobilize bioactive compounds onto the matrix	131-133
TRAP	1) Require no synthesizing photo-affinity probes;<br>2) The metabolite of interest for target deconvolution is feasible	1) Protein lysine needs to be labeled;<br>2) The presence of the false-positive results	136
LRP	Without chemical structural modification of biomolecules	The lysine reactivity based on the native microenvironments of lysine local structures	142-144
Native-MS	1) Non denaturing condition<br>2) Trace sample and fast process	1) Low detection sensitivity for low abundance protein targets or weakly bound complexes<br>2) High sample purity, avoiding interference from salts or detergents on mass spectrometry signals	146-149

17% Overall Similarity

Top sources found in the following databases:

- 11% Internet database
- 16% Publications database
- Crossref database
- Crossref Posted Content database
- 0% Submitted Works database

TOP SOURCES

The sources with the highest number of matches within the submission. Overlapping sources will not be displayed.

1	livrepository.liverpool.ac.uk<br>Internet	<1%
2	nature.com<br>Internet	<1%
3	omicsdi.org<br>Internet	<1%
4	Feiyan Chen, Chu Li, Huiying Cao, Hantao Zhang, Cai Lu, Ruimei Li, Zhu...<br>Crossref	<1%
5	pubmed.ncbi.nlm.nih.gov<br>Internet	<1%
6	frontiersin.org<br>Internet	<1%
7	pesquisa1.bvsalud.org<br>Internet	<1%
8	Hui Zhang, Jiangyu Yao, Guyu Xiao, Jianhui Xie, Shuying Mao, Chengho...<br>Crossref	<1%

9	Ying-Shan Ren, Hui-Lin Li, Xiu-Hong Piao, Zhi-You Yang, Shu-Mei Wang...<br>Crossref	<1%
10	mdpi.com<br>Internet	<1%
11	Kejia Li, Shijie Chen, Keyun Wang, Yan Wang et al. "A peptide-centric lo...<br>Crossref	<1%
12	Meng Cui, Yang Du. "Native mass spectrometry for characterization of ...<br>Crossref	<1%
13	hdl.handle.net<br>Internet	<1%
14	researchgate.net<br>Internet	<1%
15	tandfonline.com<br>Internet	<1%
16	hindawi.com<br>Internet	<1%
17	pmc.ncbi.nlm.nih.gov<br>Internet	<1%
18	science.gov<br>Internet	<1%
19	He Meng, Michael C. Fitzgerald. "Proteome-Wide Characterization of P...<br>Crossref	<1%
20	Ning Sheng, Zhihui Zhang, Hao Zheng, Congyu Ma, Menglin Li, Zhe Wa...<br>Crossref	<1%

21	ncbi.nlm.nih.gov<br>Internet	<1%
22	meeting.bsc.org.cn<br>Internet	<1%
23	scholarbank.nus.edu.sg<br>Internet	<1%
24	Shu-Jie He, Jun Li, Jie-Chun Zhou, Zhi-You Yang, Xi Liu, Yue-Wei Ge. "C...<br>Crossref	<1%
25	Ting-Ting Liu, Ke-Wu Zeng. "Recent advances in target identification te...<br>Crossref	<1%
26	pubs.rsc.org<br>Internet	<1%
27	ghrnet.org<br>Internet	<1%
28	semanticscholar.org<br>Internet	<1%
29	Yanan Li, Jiawen Lyu, Yan Wang, Mingliang Ye, Hailin Wang. "Ligand M...<br>Crossref	<1%
30	coek.info<br>Internet	<1%
31	encyclopedia.pub<br>Internet	<1%
32	Liping You, Tao Wang, Wenxuan Li, Jinghao Zhang et al. "Xiaozhi form...<br>Crossref	<1%

33	new.epo.org<br>Internet	<1%
34	onlinelibrary.wiley.com<br>Internet	<1%
35	oup.silverchair-cdn.com<br>Internet	<1%
36	Chengli Yu, Xiuzhen Chen, Weiwei Xu, Simin Li, Qian Chai, Yinan Zhang....<br>Crossref	<1%
37	Xuan Jiang, Kinyu Shon, Xiaofeng Li, Guoliang Cui, Yuanyuan Wu, Zhon...<br>Crossref	<1%
38	d-nb.info<br>Internet	<1%
39	Yan Liu, Pu Wang, Weina Hu, Da Chen. "New insights into the roles of p...<br>Crossref	<1%
40	Yujie Lu, Jie Ji, Simeng Chu, Fukui Shen, Wen Yang, Wei Lei, Min Jiang,...<br>Crossref	<1%
41	Gajanan Sathe, Gopal P. Sapkota. "Proteomic approaches advancing ta...<br>Crossref	<1%
42	Jangho Lee, Hyo-Kyoung Choi, Hee Soon Shin, Gun-Dong Kim. "Natural...<br>Crossref	<1%
43	Zixuan Zhen, Lina Yin, Tingting Niu, Asma Rehman, Yang Liu, Kewu Zen...<br>Crossref	<1%
44	molpharm.aspetjournals.org<br>Internet	<1%

45	Elva Morretta, Alessandra Tosco, Carmen Festa, Matteo Mozzicafredd...<br>Crossref	<1%
46	Mateusz Wagner, Bingsen Zhang, Arnaud Tauffenberger, Frank C. Schr...<br>Crossref	<1%
47	Simone Schopper, Abdullah Kahraman, Pascal Leuenberger, Yuehan Fe...<br>Crossref	<1%
48	Zijun Geng, Meiqi Chen, Qixuan Yu, Shuoxi Guo, Tianli Chen, Da Liu. "Hi...<br>Crossref	<1%
49	gcris2.iyte.edu.tr<br>Internet	<1%
50	ipfs.io<br>Internet	<1%
51	journal.zums.ac.ir<br>Internet	<1%
52	annualreviews.org<br>Internet	<1%
53	Baptiste Mouysset, Marion Le Grand, Luc Camoin, Eddy Pasquier. "Poly...<br>Crossref	<1%
54	Gnatenko, Dmitri V., Xiao Xu, Wei Zhu, and Valentina A. Schmidt. "Tran...<br>Crossref	<1%
55	Jack L. Bennett, Giang T. H. Nguyen, William A. Donald. "Protein–Small...<br>Crossref	<1%
56	Lu Ma, Menglong Li, Shanshan Gou, Wei Wang, Kangdong Liu, Yueteng ...<br>Crossref	<1%

57	Troy, Cassandra L. C.. "The Potential of Solutions Journalism for Clima...<br>Publication	<1%
58	Zheyi Liu, Ye Zhou, Jing Liu, Jin Chen, Albert J.R. Heck, Fangjun Wang. ...<br>Crossref	<1%
59	air.unimi.it<br>Internet	<1%
60	cdn.elifesciences.org<br>Internet	<1%
61	discovery.researcher.life<br>Internet	<1%
62	esp.org<br>Internet	<1%
63	Ben Barris, Avrohom Karp, Menachem Jacobs, William H. Frishman. "T...<br>Crossref	<1%
64	Federico Riccardi Sirtori, Alessandra Altomare, Marina Carini, Giancarl...<br>Crossref	<1%
65	Fei Feng, Weiyue Zhang, Yifeng Chai, Dandan Guo, Xiaofei Chen. "Label...<br>Crossref	<1%
66	Jia-Luo Huang, Xue-Long Yan, Wei Li, Run-Zhu Fan et al. "Discovery of ...<br>Crossref	<1%
67	Kejia Li, Shijie Chen, Keyun Wang, Yan Wang et al. "A peptide-centric lo...<br>Crossref posted content	<1%
68	Qi-Qi Liang, Zi-Jian Shi, Tao Yuan, Si-Yuan Chen et al. "Celastrol inhibit...<br>Crossref	<1%

69	Yi Li, Xingchao Yang, Fei Zheng, Hanqing Zhang, Chang Shao, Ning Wa...<br>Crossref	<1%
70	Zhihuai Wang, Yinjie Zhang, Yuhang Shen, Chunfu Zhu, Xihu Qin, Yuan ...<br>Crossref	<1%
71	Zhiruo Zhang, Yueying Yang, Yang Xu, Yang Liu, Hua Li, Lixia Chen. "M...<br>Crossref	<1%
72	Ziyi Chen, Wenwen Ding, Xiaoxue Yang, Tiangong Lu, Ying Liu. "Isoliqui...<br>Crossref	<1%
73	medrxiv.org<br>Internet	<1%
74	"Activity-Based Protein Profiling", Springer Nature, 2012<br>Crossref	<1%
75	"Mapping a comprehensive targetome of glycolytic metabolites in can...<br>Crossref	<1%
76	Giang T. H. Nguyen, Jack L. Bennett, Sherrie Liu, Sarah E. Hancock, Da...<br>Crossref	<1%
77	Hong-Wei Zhang, Chao Lv, Li-Jun Zhang, Xin Guo et al. "Application of ...<br>Crossref	<1%
78	Jiawen Lyu, Chengfei Ruan, Xiaolei Zhang, Yan Wang, Kejia Li, Minglian...<br>Crossref	<1%
79	Liu Yang, Chen-Wan Guo, Qi-Ming Luo, Zi-Fan Guo, Ling Chen, Yasushi ...<br>Crossref	<1%
80	Liu, Yuan-Zhen, and Ming-Quan Guo. "Chemical proteomic strategies f...<br>Crossref	<1%

81	Min Huang. "PTM-centered therapy in malignant tumors: A new story o...<br>Crossref	<1%
82	Mozaniel Santana de Oliveira, Leo M.L. Nollet. "Bioactive Compounds -...<br>Publication	<1%
83	Teeratas Kijpornyongpan, Alexa Schwartz, Allison Yaguchi, Davinia Sal...<br>Crossref	<1%
84	Xin Luan, Wei-Dong Zhang, Guang-Bo Ge. "Interdisciplinary strategies f...<br>Crossref	<1%
85	Yi-Han Chang, Hsin-Yi Hung. "Recent advances in natural anti-obesity c...<br>Crossref	<1%
86	Yixin Wang, Fan Wang, Wenxiu Liu, Yifei Geng, Yahong Shi, Yu Tian, Bin...<br>Crossref	<1%
87	discovery.dundee.ac.uk<br>Internet	<1%
88	edoc.hu-berlin.de<br>Internet	<1%
89	link.springer.com<br>Internet	<1%
90	m.earticle.net<br>Internet	<1%
91	pubs.acs.org<br>Internet	<1%
92	pure.rug.nl<br>Internet	<1%

93	biotechsupportgroup.com<br>Internet	<1%
94	Shubham Upadhayay, Sidharth Mehan. "Targeting Nrf2/HO-1 anti-oxid...<br>Crossref	<1%
95	Wei Shen, Xiao-Long Hu, Si-Yuan Li, Lun Li, Xiao-Wei Dong, Hao Liu, Jia...<br>Crossref	<1%
96	Xiaolei Zhang, Chengfei Ruan, Yan Wang, Keyun Wang, Xiaoyan Liu, Jia...<br>Crossref	<1%
97	Yuyu Zhu, Zijun Ouyang, Haojie Du, Meijing Wang, Jiaojiao Wang, Haiya...<br>Crossref	<1%
98	"Top 10 academic progress on traditional Chinese medicine in 2022", S...<br>Crossref	<1%
99	Dalinda Isabel Sánchez-Vidaña, Rahim Rajwani, Man-Sau Wong. "The U...<br>Crossref	<1%
100	Dongyoung Kim, Hui-Yun Hwang, Jin Young Kim, Ju Yeon Lee, Jong Sh...<br>Crossref	<1%
101	Gen Li, Xuling Peng, Yajing Guo, Shaoxuan Gong, Shijie Cao, Feng Qiu. "...<br>Crossref	<1%
102	Haiming Xiao, Xiaohong Sun, Zeyuan Lin, Yan Yang, Meng Zhang, Zhan...<br>Crossref	<1%
103	Hui Yi Liew, Xin Yoong Tan, Hong Hao Chan, Kooi Yeong Khaw, Yong S...<br>Crossref	<1%
104	J. Chang, Y. Kim, H. J. Kwon. "Advances in identification and validation...<br>Crossref	<1%

105	Jichao Sun, Nayana Prabhu, Jun Tang, Fan Yang, Lin Jia, Jinan Guo, Ke...<br>Crossref	<1%
106	Jonathan G Van Vranken, Jiaming Li, Dylan C Mitchell, José Navarrete-...<br>Crossref	<1%
107	Khawaja Husnain Haider. "Handbook of Regenerative Medicine - Stem ...<br>Publication	<1%
108	Miaomiao Liu, Wesley C. Van Voorhis, Ronald J. Quinn. "Development ...<br>Crossref	<1%
109	Weikai Guo, Manjie Wang, Zhengfan Yang, Danyang Liu, Borui Ma, Yan...<br>Crossref	<1%
110	Xin Luan, Li-Jun Zhang, Xiao-Qin Li, Khalid Rahman, Hong Zhang, Hong...<br>Crossref	<1%
111	Yanbei Tu, Lihua Tan, Tao Lu, Kai Wang et al. "Glytabastan B, a coumes...<br>Crossref	<1%
112	Yulong Bai, Shengnan Zhang, Hui Dong, Yu Liu, Cong Liu, Xin Zhang. "A...<br>Crossref	<1%
113	Zhenzhen Yan, Ling Zhong, Wandi Zhu, Sookja Kim Chung, Panpan Hou...<br>Crossref	<1%
