# Supplementary material for: Recent advances in proteomic strategies for target identification of traditional Chinese medicine
Source: J Pharm Anal. 2025 Dec 10;16(7):101516. doi: 10.1016/j.jpha.2025.101516 (PMC13377450; doi:10.1016/j.jpha.2025.101516)
Supplement: Multimedia component 2 [file mmc2.docx]

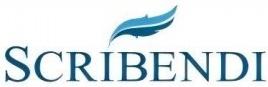


Certificate of Editing and Proofreading


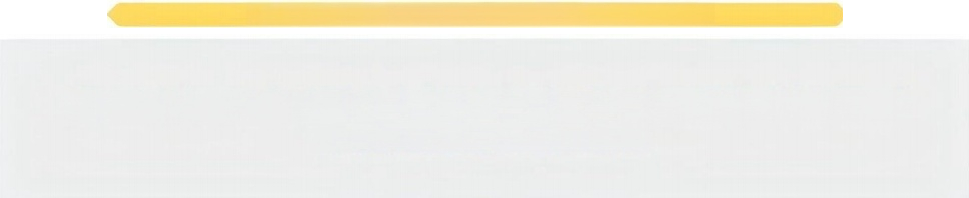


This certifies that a version of the document titled

**Recent Advances in Proteomic Strategies for Drug Target Identification of**

**Traditional Chinese Medicine**

**authored by**

**Ren Yan**

was edited and/or proofread by Scribendi as order number

**993584**


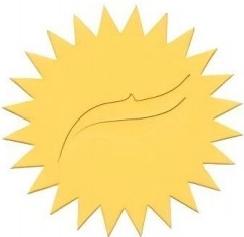
for clarity,consistency,and correctness according to the requirements and guidelines specified by the client.

**Wed,06 Mar 2024**

Scribendi ln

**SCRIBENDI INC.**

**405 RIVERVIEW DRIVE**

**CHATHAM,ON N7M ON3 CANADA**

**+1(519)3511626**

[www.scribendi.com](https://www.scribendi.com)
